# Implementation of Primary Psychological Healthcare Policy to Address the Risk of Depression in Underprivileged Children and Adolescents, in the Entire Lower-Middle-Economic-Status City of China: An Observational, Multicenter, and Single-Arm Cohort Study

**DOI:** 10.1155/da/5572365

**Published:** 2025-09-11

**Authors:** Wei Li, Xue-rong Liu, Qianyu Zhang, Lei Xia, Yanyan Li, Xiaobing Tian, Jie Gong, Jidong Ren, Chang Shen, Yi Wu, Ji Chen, Chuan-Peng Hu, Jing-Xuan Zhang, Ting Xu, Yuanyuan Hu, Bowen Hu, Ni Yan, Tingyong Feng, Zhengzhi Feng, Zhiyi Chen

**Affiliations:** ^1^Experimental Research Center of Medical and Psychological Science (ERC-MPS), School of Psychology, Army Medical University, Chongqing, China; ^2^Department of Public Management, Chongqing University, Chongqing, China; ^3^Department of Epidemiology and Public Health Statistics, North Sichuan Medical College, Nanchong, China; ^4^Department of Clinical Psychology, Nanchong Psychosomatic Hospital (The Sixth People's Hospital of Nanchong), Nanchong, China; ^5^Department of Psychology and Behavioral Sciences, Zhejiang University, Hangzhou, China; ^6^School of Psychology, Nanjing Normal University, Nanjing, China; ^7^The Clinical Hospital of Chengdu Brain Science Institute, MOE Key Laboratory for Neuroinformation, University of Electronic Science and Technology of China, Chengdu, China; ^8^The Institute of Psychology, Chinese Academy of Sciences, Beijing, China; ^9^School of Psychology, Beijing Normal University, Beijing, China; ^10^Key Laboratory of Cognition and Personality, Ministry of Education, Faculty of Psychology, Southwest University, Chongqing, China

**Keywords:** depression, low–middle-income areas, primary psychological health care policy, underprivileged children/adolescents

## Abstract

**Background:** Given the historically high incidence of depressive disorders among children/adolescents, efforts to implement universally accessible primary psychological health care policies have been undertaken globally. However, the practical implementation and its association with depression risk reduction remain uncertain, particularly for underprivileged children/adolescents who are underrepresented in the current system.

**Methods:** A large-scale cohort of underprivileged children/adolescent population aged 6–18 was enrolled (*n* = 290,239). Subgroups with specific underprivileged conditions were identified, including de facto unattended children/adolescents (dfUCA), orphans, and children/adolescents facing especially difficult circumstances, “left-behind” and “single-parent” children/adolescents. A subgroup of matched typically developing individuals was also included. These subgroups underwent longitudinal assessments for the incidence of identifying depression on Oct 30, 2022 (baseline, before implementing primary psychological health care policy), May 21, 2023 (half year follow-up), and Oct 29, 2023 (1-year follow-up), respectively.

**Results:** At baseline, nearly twice as high incidence of depression was found in the underprivileged group (13.9%, 95% confidence interval [CI]: 13.7–14.1) as in the control group (7.5%, 7.2–7.7). After the implementation of the primary psychological policy, at the half year follow-up, a notable decrease in the incidence of depression was observed in both the underprivileged group (5.8%, relative risk reduction (RRR) = 51.6%, 51.5–51.7, *p* < 0.001) and the typically developing group (4.0%, RRR = 34.5%, 27.9–41.0, *p* < 0.001), particularly among orphan girls aged 12–18. The observed changes in depression incidence across all underprivileged populations were statistically noninferior compared to the typically developing group (all *p* < 0.001). At the 1-year follow-up, the observed benefits were consistent across all subgroups when compared to baseline. The average expenditure per child/adolescent was $1.6 in implementing such a health care policy.

**Conclusions:** Implementing the primary psychological health care policy is associated with a reduction in the citywide risk of depression among underprivileged children/adolescents in low–middle-income areas.

## 1. Introduction

The comprehensive prioritization of mental health for children and adolescents has garnered global consensus due to the unprecedented surge in the incidence of depression among these populations [1, 2]. For example, the prevalence of depression in younger, particularly in children and adolescents, has nearly doubled worldwide, with particularly rapid increases in third-world nations over the past two decades [3–6]. In China, more than 30 million children/adolescents (24.5% of this national population) are suffering from depressive disorders, with up to 3.74 deaths per 100,000 children/adolescents by suicide in 2021 [7, 8]. To address the unpredictable surges and consequential impacts of this health menace on children/adolescents, local communities, governments, and health sectors globally are committed to implementing a primary mental health care policy as one promising solution, aiming to provide equitable and accessible psychological services for all children/adolescents in need.

As didactic cases, the World Health Organization (WHO) recently updated the Comprehensive Mental Health Action Plan 2013–2030, which is specialized to underscore the promotion of early (primary) mental health care service for children/adolescents, encouraging countries to integrate such service into national primary health care policies to reduce the risk of depression from a macro perspective [9]. Likewise, the Lancet-World also urged global united actions for alleviating disadvantages of depression by providing primary mental health service/care for adolescents [10, 11]. In China, the National Mental Health Service Guideline is also dedicated to implementing a nationwide supporting policy to decrease depression “epidemics” in adolescent students, especially for underrepresented ones with underprivileged conditions [12]. Despite ambitions, there remain gaps in both real-world data and high-quality evidence to illuminate the benefits of such policy implementation in managing the risk of depression among children/adolescents.

Though the implementation of primary psychological health care policy is understudied, conducting regular and widespread mental health surveillance has been argued as one feasible solution to control the overall level of childhood depression across a region or country and is currently dominant in existing health care policies. By building upon the national digital mental health surveillance system in the United States, all children have undergone annual surveys screening for depressive symptoms to monitor population-based risks of depression [13]. Results demonstrated the significantly increased rates of recognition and ensuing treatment for childhood depression after implementing this surveillance [13, 14]. Having benefited from such regular screenings on children, the United States Preventive Services Task Force (USPSTF) has embarked on stretching this surveillance system to adults as general policy to go against depression “epidemics” [15, 16]. Rather than a conceptual framework of setting up primary psychological health care, such public health surveillance systems are currently well-established in broad developed countries, with prominent health benefits to mitigate depression, such as in Germany [17], Canada [18], Saudi Arabia [19], and France [20]. Despite remarkable contributions, relying on the surveillance systems to address childhood depression alone is intensively critiqued for unexpectedly increasing the risks of childhood depression in practice [21, 22]. Even worse, screening for depression that is embedded in the surveillance system was originally designed to support decision-making on public health policy, and thus remains an open-loop health care framework without any follow-ups or evidence-based treatments [23]. As a result, several national medical authorities or health care providers no longer recommended regular depression screening in the surveillance systems, particularly for children/adolescents [24–26]. Therefore, despite its prevalence, such discrepancies in terms of safety and purpose of the surveillance system should warrant caution when adopting this surveillance system in children/adolescents.

Rather than specific systems or interventions, community-based and comprehensive mental health services are rapidly developing as a promising solution to control children/adolescent depression, particularly in areas under economically disadvantaged conditions. The United Nations International Children's Emergency Fund (UNICEF) and WHO have recently sponsored several low- and middle-income countries (LMICs) to build community-based mental health centers, along with investments in infrastructure supporting community-based psychological services [27–31]. Although many LMICs have heeded the call to introduce such community-based primary psychological health care policies, they have approached it in varied manners, lacking methods and strategies to promote an integrated perspective across policy domains [32–34]. To fulfill the policy vision, the government-sponsored Healthy China 2030 Plan, one of the largest medical reforms in China, has announced a budget of over ¥1.1 billion dedicated to implementing a community-based primary psychological health care policy, integrating multiple strategies, such as increasing mental health education, conducting early depression screenings, enhancing families' support networks, and forming multidisciplinary expert groups (e.g., social carers, psychologists, and psychiatrists) within communities, to promote population-based mental health [35]. Compared to national surveillance systems passively monitoring population-based dynamics of depression, community-based health care policies have shown superior real-world benefits in controlling depression [36, 37]. Despite the policy's intentions, a key challenge is the substantial variation in reducing the risk of depression through community-based implementations across different populations and countries, driven by existing racial disparities and economic inequalities [38, 39]. Thus, reaching universal health care benefits for mental health in underprivileged children/adolescents should still be prioritized when implementing such policies to mitigate depression.

It is worth noticing that the primary psychological health care policy is built on a nonfinancial medical mode that is largely carried out by nonprofit entities [40]. In this case, it may limit the high-quality care due to underpaid medical services and cost-saving incentives for the health care workforce [40]. Furthermore, as one of the most overarching goals of implementing a regional or even national primary health care policy, health equality is paramount in addressing and controlling the overall risk of depression incidence for all children and adolescents. However, certain subgroups of children and adolescents are noticeably underrepresented in such health care policy, potentially undermining the principle of health equality. Compared to adults, children/adolescents, especially girls or transgender populations, have fewer chances of reaching such primary mental health services as a result of more stigma and economic burdens [41]. Supporting this argument, the children/adolescents facing disadvantages in parenting, education and economic status (e.g., orphans, unattended and “left-behind” children), are found to be highly underrepresented in accessing those health care resources and mental health services provided through the implementation of such health care policies, sharpening the risks of such health inequities [42–44]. Thus, the benefits of implementing a primary psychological health care policy, particularly regarding their real-world generalizability and practical benefits, for such underrepresented children/adolescents in the LMIC areas, still remain unclear.

**Figure 1:**
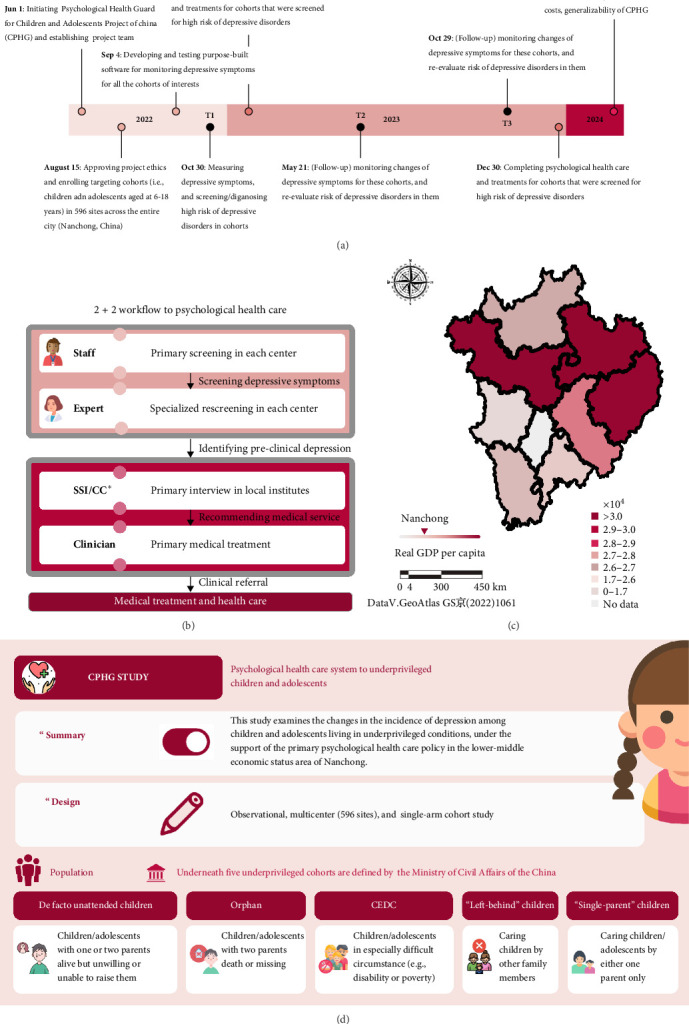
Diagram for research framework and features. (a) Streamlines the research timeline and overview for the Psychological Health care Guard of China for Children and Adolescents Project of China (CPHG) at the current phrase. (b) Illustrates the workflow of the “2 + 2 model” psychological health care that the current study established, with the first two rounds for screening depressive symptoms and later two rounds for psychological cares. As an open-loop workflow, children/adolescents may be recommended for in-patient medical treatment and health care outside of this “2 + 2 model” workflow. *⁣*^*∗*^qualified healtcare specialist in school or insititute (SSI)/community-health care consultant (CC). (c) Plots a geospatial map to describe the geographic distribution of the current sample by using the map dataset from the “Aliyun” (Ali., Inc., Hangzhou, China) with permission (GS(2022)1061). The data for the economic rank of Nanchong was derived from the National Bureau of Statistics (China). (d) Provides an overview of the current study, including the research summary and study design.

In response to these challenges, we formulated this primary psychological health care policy entitled the Psychological Health Guard for Children and Adolescents Project of China (CPHG), targeting underrepresented children/adolescents aged 6–18 years living under underprivileged conditions in the whole city (Nanchong, Sichuan, China). We capitalized on an observational, multicenter, and single-arm cohort design to examine changes in the incidence of depression across baseline (prior to implementing this policy, Oct 30, 2022), half year (May 21, 2023), and 1-year follow-ups (October 29, 2023) (Figure 1a). As a pilot city representing lower-middle-economic-status areas, nearly 60% of the children/adolescent population was identified as “left-behind” in Nanchong, with relatively low urban health care expenditure and gross domestic product (GDP) per capital in China. This primary psychological health care policy has been implemented by creating a psychological service model that integrates medical institutions, schools, families, communities, and charitable organizations, in which the core project was the “2 + 2 model” (Supporting Information 1), including two rounds of screening for risk of depression and two rounds of early psychological interventions (Figure 1b–d).

## 2. Methods

### 2.1. Study Design and Participants

This is an observational, multicenter, and single-arm cohort study of children/adolescents in Nanchong, a city in lower-middle-economic-status areas of China, aiming to examine changes in the incidence of depression under the support of the primary psychological health care policy at half year and 1-year follow-up. Given the observational, single-arm cohort design, we only identify associations between policy implementation and reduced depression incidence. This study is implemented by the government-sponsored CPHG Group. An early version of this paper has been posted as a preprint on SSRN [45].

This group utilized each school and social-care institute (e.g., orphanage, community-care site, and children's hospital) as a primary health care center for enrolling eligible children/adolescents covering almost all the targeted populations in this city. A total of 596 centers were established, nearly evenly recruiting these children/adolescents from each borough (including almost all rural and urban areas). To guarantee gender equality, this group screened all eligible girls in this city by matching the unique identifier number (UID) in the educational registration system. To achieve equality for the underprivileged ones, children/adolescents outside family, school, or social-care systems were manually checked with aid from local civil affairs authorities.

313,201 children/adolescents aged 6–18 years living in the administrative areas of Nanchong were initially covered under this health care policy. Informed consents were digitally signed by either their parents or legal caregivers. Children/adolescents were excluded from this policy if they or their legal caregivers refused to be involved. The sampled distribution for included children/adolescents was perceived to be roughly consistent with the population-based geospatial pattern, with intensive population in the middle and eastern areas of Nanchong (Figure 1c).

By self-reported classifications, the included children/adolescents were further categorized into five underprivileged conditions that legally required social care or into typically developing ones (i.e., under normal parenting and family care), on the basis of benchmarks formulated by the Ministry of Civil Affairs in China, including de facto unattended children/adolescents (dfUCA), orphans, children/adolescents in especially difficult circumstance (CEDC), “left-behind”, and “single-parent” children/adolescents. dfUCA refer to individuals whose parents both meet the criteria of severe disability, severe illness, imprisonment, mandatory isolation for drug rehabilitation, other restrictions on personal freedom, missing status, revoked guardianship, or deportation; or with one parent deceased or missing and the other under any of these criteria. Orphans refers to unmarried adolescents under 18 years old who lost both parents or cannot locate either parent. CEDC refers to children/adolescents in families with a primary member who is elderly, physically weak, alone without care, or incapable of working, or families earning less than $8206 per year, requiring legally mandated social care. “Left-behind” children/adolescents refer to those without parental care due to parents (or one of them) working away from home or long hours. “Single-parent” children/adolescents refer to those raised by only one parent due to the other's death, divorce, or abandonment. All individuals would be labeled under only one underprivileged condition. Children/adolescents who were identified as overlapping across these conditions were excluded from this analysis. Structural and detailed criteria for each unprivileged condition can be found in the Supporting Information 2 (Methods 1). A portion of children/adolescents who failed to be identified under any of the above conditions were also covered under this psychological health care policy but were excluded from the current cohort study.

This study was approved by the Institutional Review Board (IRB) of The Sixth People's Hospital of Nanchong (IRB ID: 2022002). The research proposals and hypotheses were preregistered at AsPredicted (#142898). The CHPG project was wholly overseen by the Department of Civil Affairs of Sichuan Province.

### 2.2. Procedure

This psychological health care policy was implemented by integrating medical institutions, schools, families, communities, and charitable organizations and aiming at enhancing the mental health of children/adolescents. The policy covered one key project—“2 + 2” psychological health care services for children/adolescents and three supportive components, including role-specific mental health knowledge training for administrative leaders, head teachers, subject teachers, and full-time or part-time mental health teachers, psychological service facilities development, and mental health science popularization for parents and youth-related social volunteers (Supporting Information 1 and 3). The “2 + 2” psychological health care services were conducted semiannually and included two sequentially structured rounds of psychological screening for depression and two sequentially structured rounds of psychological intervention for “at-risk” children/adolescents with depression (Figure 1b). In the first round of psychological screening, we utilized a purpose-built app to measure children/adolescents' depressive symptoms using the Center for Epidemiological Studies-Depression Scale (CES-D), where individuals with scores of 16 or higher were identified as having depressive experiences, according to the CES-D scoring criteria [46]. This initially psychological screening was guided at each center by staff who were trained beforehand in the CPHG group (Supporting Information 2: Table S1). In the second round of screening the risk of depression, these children/adolescents who were identified as having depressive experiences (in the last round) underwent rescreenings using Zung's self-report depression scale (SDS), to identify the presence of depressive symptoms, in conjunction with subclinical evaluations from experts, who are qualified in psychological health care at each center (Supporting Information 2: Table S2). According to the Chinese norms of the SDS, scores ranging from 63 to 72 indicate moderate depression, and scores above 72 indicate severe depression, both of which are considered “at-risk” for developing adverse outcomes (e.g., bullying, obesity, or suicide). Therefore, these children/adolescents with moderate to severe depression were identified as having “at-risk” depression and were screened out to proceed with two-round early psychological intervention.

In the first round of early psychological interventions, one psychological health care specialist was assigned to provide primary psychological interview/intervention (i.e., measurement validation, mood status evaluation, social support evaluation, and extreme risk evaluation) to each child/adolescent with identified “at-risk” depression, matching their school/institute (center) (Supporting Information 1). All these specialists were professionally qualified and received standardized retraining before the CPHG was implemented, covering basic psychological counseling skills, psychological crisis intervention, and identification and management of severe mental disorders, and so on (Supporting Information 3). In addition, one qualified community health care consultant was designated to provide such primary psychological interventions for those children/adolescents with depression who were outside schools and institutes. Individuals without positive evaluation results received psychological intervention on a voluntary basis. Once individuals reported “yes” in either one of the above evaluations, they were recommended for further medical service in the second round of early psychological interventions. In the second round, a portion of children/adolescents equally received primary clinical medical treatment from clinical psychiatrists in government-sponsored mental health centers (hospital) after clinical assessment and diagnosis (Supporting Information 2: Table S3). As part of the open loop primary psychological health care, children/adolescents were recommended for further inpatient treatment upon receiving medical referral advice.

These two-round psychological screenings were conducted from October 30, 2022, to November 14, 2022. The screening results were used as a baseline (T1) for estimating the incidence of depression in these children/adolescents in the entire city. On January 19, 2023, the two-round early psychological interventions were implemented for all those identified with depression in the screenings. On May 21, 2023 (T2), the half year follow-up screenings for incidence of depression in these children/adolescents were conducted, without the two-round early psychological interventions. On October 29, 2023 (T3), the 1-year follow-up screenings for the same cohort were completed.

### 2.3. Statistical Analysis

Given that the main outcome of this study was the changes in the incidence of identified depression, the frequency (rate) of each survey was calculated, with a 95% confidence interval (CI) estimated by the bootstrapping method (*n* = 5000) and with corresponding inferential statistics estimated by a one-sample ratio Z test for proportions. Independent between-group contrasts for frequency (rate), such as girls vs. boys, were estimated for inferential statistical outcomes using a two-sample *Z* test for proportions.

For the observational cohort study, we used the log-binomial regression model to estimate relative risk (RR) of developing depression for children/adolescents with each underprivileged condition, in contrast to typically developing ones (Supporting Information 2). As sensitivity analyses, we further calculated RR by adjusting for sex, age, centers, number of offspring, and living areas.

To examine changes in the incidence of depression following the implementation of the primary psychological health care policy in these underprivileged cohorts, the paired McNemar change test was used. Minimum frequencies for each cell in all the tests were greater than 25, and the asymptotic *p* values were estimated. Furthermore, RR reduction (RRR), absolute risk reduction (ARR) and number needed to treat (NNT) were calculated to quantitatively estimate the change in the incidence rate of depression before and after the implementation of this health care policy (Supporting Information 2, Methods 4). To perform sensitivity analyses, we conducted 1:1 propensity score matching between the typically developing cohort and underprivileged cohort based on the following covariates: age, sex, number of offspring, center, and living area. Then we recalculated RRR, ARR, and NNT for each matched cohort.

We further conducted noninferiority tests to determine whether the change in the incidence/risk of depression among children and adolescents in underprivileged conditions was noninferior to that observed in typically developing peers. Estimates for sample size have been detailed in the Supporting Information 2. Given no evidence for a benchmark of such effect, the noninferiority difference boundary was iterated from 5% to 10% in pilot tests.

By including an independent 14-year longitudinal dataset drawn from the China Family Panel Studies (CFPS, 2010–2024; www.isss.pku.edu.cn/cfps/), we established the observational control group by recruiting 730 Chinese children/adolescents who were successfully followed to measure depressive symptoms in 2016, 2018, and 2020 (Supporting Information 2, Methods 4), which was outside the implementation of the primary psychological health care policy. The Cochran-Armitage test was used to statistically examine the incidence trends of identifying depression for this observational control group, in contrast to the samples in the current study.

Data were gathered and pre-processed using the purpose-built app, including outlier detection, missing data removal, and lie-reaction detection. The Bonferroni–Holm correction was used to adjust *p*-values for all the multiple comparisons. Statistics were calculated using the lme package and MatchIt package in R (version 4.2.1) and SPSS v24.

### 2.4. Role of the Funding Source

The funder of the study had no role in the study design, data analysis, data interpretation, writing of the report, or the decision to submit for publication.

## 3. Results

At the baseline (T1), 290,239 children/adolescents aged 6–18 years were finally included from 596 centers covering almost all the targeted populations of Nanchong, China. Five cohorts were categorized for posing underprivileged conditions that required legally mandated social care, including dfUCA (*n* = 2444, 0.8%), orphan children/adolescents (*n* = 762, 0.2%), CEDC (*n* = 18,419, 6.2%), “left-behind” children/adolescents (*n* = 179,877, 60.5%), and “single-parent” children/adolescents (*n* = 48,270, 16.2%). Further, 40,467 children/adolescents (13.60%) who were free from the above underprivileged conditions were included to be a comparable typically developing cohort. Demographic characteristics for included cohorts have been detailed in Table 1.

**Figure 2:**
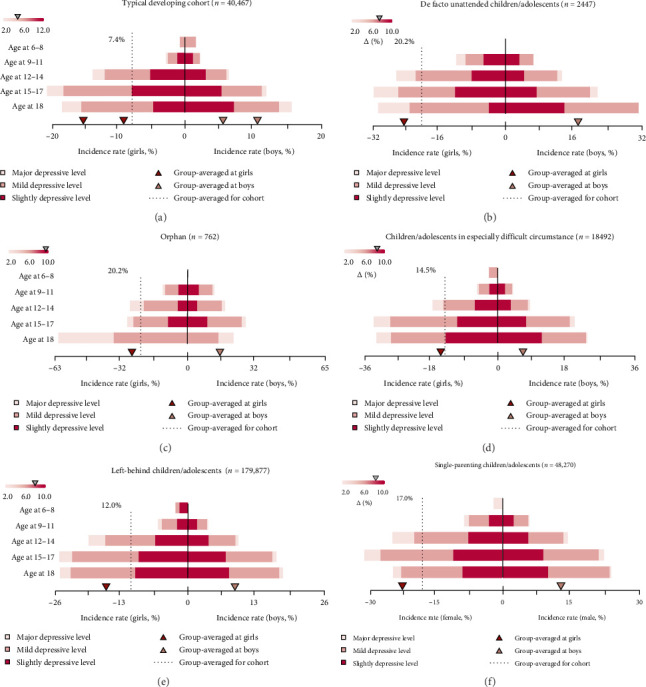
Incidence rates of identifying depression for each included cohort at the baseline. The presence of depressive symptoms was estimated by Zung's Self-reported Depression Scale (SDS). Criterion for ranking slight, mild, and major depressive levels could be found in the Supporting Information 2. The bar embedded in the left-upper corner indicates the relative increases in incidence rates of identify depression for girls compared ones in the boys. (a) Typical developing cohort (*n* = 40,467). (b) De facto unattended children/adolescents (2447). (c) Orphan (*n* = 762) (d) Children/adolescents in especially difficult circumstance (*n* = 18,492). (e) Left-behind children/adolescents (*n* = 179,877), (f) Single-parenting children/adolescents (*n* = 48,270).

**Figure 3:**
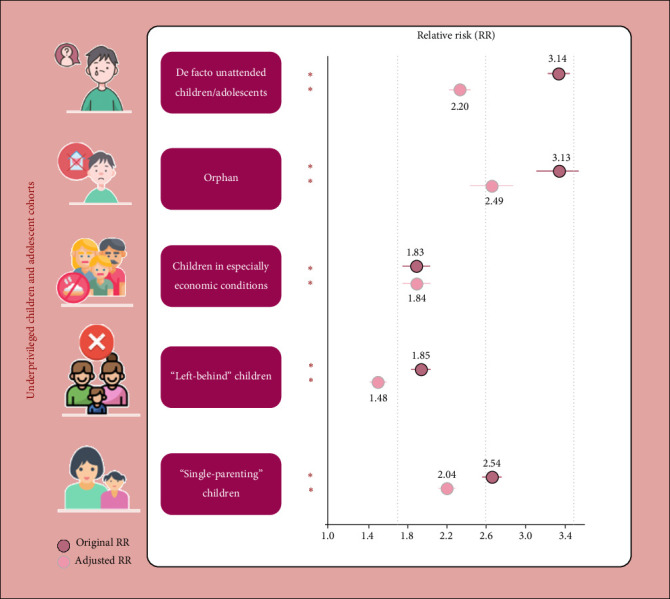
Relative risk (RR) of identifying depressive symptoms for these underrepresented, underprivileged children/adolescents compared to the typically developing ones. The dots with dark color indicate the point estimate for the original RR, with the line behind a given dot showing the 95% confidence interval (CI). The dots with light color (along with lines) indicate the same statistics by adjusting for sex, age, data collector, living areas, and the number of offspring within the family *⁣*^*∗*^*p* < 0.001. This figure has been redrawn for cartoon style for strengthening readability.

In Nanchong at T1, the overall incidence of identifying depression in the whole population was 13.0% (95% CI: 12.9–13.1). The incidence of depression in the whole underprivileged children/adolescents (13.9%, 95% CI: 13.7–14.1) nearly doubled that of typically developing ones (7.5%, 95% CI: 7.2–7.7; Supporting Information 2: Table S5). Sex-specific incidences at different ages for cohorts with specific underprivileged conditions were presented (Figure 2 and Supporting Information 2: Table S6). An apparent uptrend in the incidence of depression was found across ages for all the above cohorts, especially with higher rates for underprivileged girls aged 12–18 years (Supporting Information 2: Table S7−S12). Further, nearly double the RR of suffering from depression was also found in all the underprivileged cohorts compared to typically developing one, adjusting for sex, age, centers, number of offspring, and living areas (Figure 3 and Supporting Information 2: Table S13–S17). Thus, compared to typically developing children/adolescents, double incidence, and RRs of depression were found in the underprivileged ones in lower–middle-economic-status areas, especially in girls aged 12–18 years.

**Figure 4:**
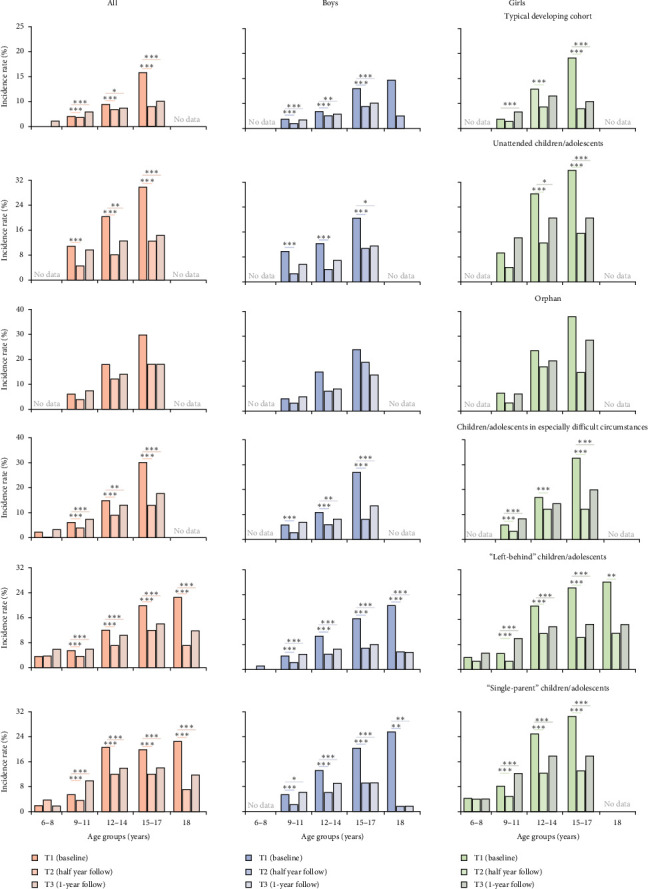
Sex-specific and age-specific changes of incidence rates of identifying depressive symptoms after implementing the primary psychological health care policy. To ensure the robustness of these findings, the data would be shown for “no data” if the frequency of participants in a given subgroup is less than 30. Age groups have been categorized at intervals of three years old (i.e., 6–8, 9–11, 12–14, 15–17, 18). Evaluations for these children/adolescents had been conducted on October 30, 2022 (T1, baseline), May 21, 2023 (T2, half year follow-up), and October 30, 2023 (T3, 1-year follow-up). *⁣*^*∗*^*p* < 0.25, *⁣*^*∗∗*^*p* < 0.01, *⁣*^*∗∗∗*^*p* < 0.001 (Bonferroni correction: *α*/2 = 0.05/2 = 0.025).

After implementing this primary psychological health care policy, 148,652 children/adolescents (51.2% from the whole population) were successfully followed up at both half year (T2) and 1-year (T3). At T2, the overall incidence of depression significantly decreased to 5.5% (95% CI: 5.4–5.6, RRR = 50.4%, ARR = 5.6%, NNT = 18, *p*_corrected_ < 0.001, McNemar's test), with the incidence decreasing to 4.0% for the typically developing cohort and to 5.8% for the underprivileged population (Supporting Information 2: Table S18). Compared to the downtrend in the incidence of depression in the typically developing cohort (RRR = 34.5%, ARR = 2.1%, NNT = 47, *p*_corrected_ < 0.001), the overall incidence of depression decreased more sharply in the underprivileged population (RRR = 51.6%, ARR = 6.1%, NNT = 16, *p*_corrected_ < 0.001) and in each specific underprivileged cohort (Supporting Information 2: Table S19–S21). Sex-specific changes for these incidences at different ages for these five underprivileged cohorts were separately presented (Figure 4 and Supporting Information 2: Table S22−S40). Specifically, following the implementation of the primary psychological health care policy, a greater reduction in depression risk was observed in the underprivileged cohort compared to the typically developing cohort (ARR = 16.7%, *p* < 0.001; Supporting Information 2: Table S20−S21). As a sensitivity validation, we have redone these analyses for those children/adolescents who were successfully followed up at half year only (*n* = 215,441, 72.4% from the whole population), showing a full replication of these findings (Supporting Information 2: Table S41−S47). As hypothesized, the observed reductions in the risk of depression among underprivileged populations were found to be significantly noninferior to those in typically developing children/adolescents (Supporting Information 2: Figure S1). In sum, in the lower-middle-economic-status areas, the risks of depression were limited after the implementation a of primary psychological health care policy, as evidenced by a significantly decreased incidence rate of depression among children and adolescents, particularly in underprivileged cohorts.

**Figure 5:**
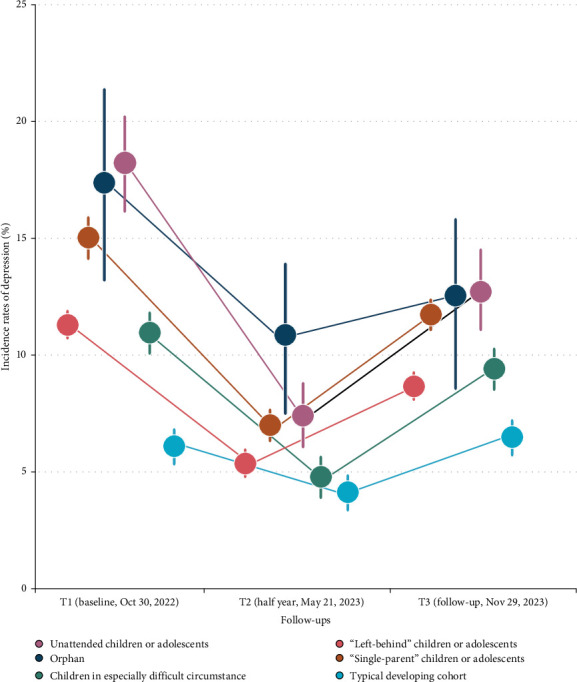
“V-shape” changes of incidence rates of identifying depressive symptoms after implementing the primary psychological health care policy at half year and 1-year follow-ups. The dots with dark color indicate the point estimate for the original RR, with the line behind a given dot showing the 95% confidence interval (CI).

At T3, these children/adolescents underwent the 1-year follow-up reevaluation. Compared to T1, the overall incidence of depression was still significantly decreased to 9.0% (95% CI: 8.9–9.2; RRR = 18.9%, ARR = 2.0%, NNT = 50, *p*_corrected_ < 0.001). For these underprivileged children/adolescents, such incidences were still found to be lower than at baseline (9.5%, 95% CI: 9.3–9.6; RRR = 20.8%, ARR = 2.4%, NNT = 42, *p*_corrected_ < 0.001) at T3, though relatively increased compared to T2 (Figure 5). Nevertheless, the incidence of depression was found to be slightly (but significantly) increased in the typically developing cohort compared to that at T1 (6.1% vs. 6.7%, RRR = −9.8%, ARR = 0.6%, NNT = 167, p _corrected_ = 0.003). Full results mentioned above could be found at Supporting Information 2: Results 4–6. In conclusion, following the implementation of this primary psychological health care policy, the downward trend in the incidence rate of depression among underprivileged children/adolescents may partially persist for 1 year.

In the sensitivity analyses, given the sample size imbalance between the typically developing cohort (*n* = 21,659) and the underprivileged cohort (*n* = 126,993), we performed 1:1 propensity score matching with the smaller cohort (i.e., typically developing cohort) as the reference group, thereby identified 21,659 individuals from the underprivileged cohort. At T2, the incidence of depression in the typically developing cohort was found significantly decreased from 6.1% (95% CI: 5.8%–6.4%) to 4.0% (95% CI: 3.7%–4.3%; RRR = 34.5%, ARR = 2.1%, NNT = 47, *p*_corrected_ < 0.001). For the underprivileged cohort, the incidence decreased significantly from 17.2% (95% CI: 16.7%–17.7%) to 7.3% (95% CI: 7.0%–7.7%; RRR = 57.4%, ARR = 9.9%, NNT = 10, *p*_corrected_ < 0.001). At T3, the incidence in the typically developing cohort slightly decreased to 6.7% (6.1% vs., 6.7%; RRR = 1.3%, ARR = 0.1%, NNT = 1000, *p*_corrected_ = 0.622), while the incidence in the underprivileged cohort was still lower than baseline (17.2% vs. 11.8%; RRR = 31.6%, ARR = 5.4%, NNT = 18, *p*_corrected_ < 0.001).

By further estimating the trends of incidence rates of identifying depression across three follow-ups, we found a statistically significant one-sided downtrend after implementing this primary psychological health care policy (*z* = −19.7, *p*_for trend_ < 0.0001), and demonstrated a statistically significant uptrend in the observational control group (i.e., CFPS sample) that was uncovered under the current health care policy (*z* = 5.0, *p*_for trend_ < 0.0001) (Supporting Information 2: Results 7). Thus, it provided secondary evidence indicating that the implementation of primary psychological health care policy coincided with a reduced risk of depression among children/adolescents covered under this policy, in contrast to children/adolescents who were uncovered.

Direct costs of implementing this primary psychological health care policy were independently estimated by accounting groups (Supporting Information 2: Results 9). A total of ¥2.8 million ($0.39 million) was directly spent for this health care, whilst ¥1.1 million ($0.15 million) were costed for supportive services (e.g., payments for staff/experts, policy promotion, psychological health care education, and data management), with (minimum) averaged ¥11.7 ($1.6) for per child/adolescent. Thus, implementing a primary low-cost psychological health care policy among these underrepresented children/adolescents, regardless of their conditions, in the lower-middle-economic-status areas is suggested as a potentially economically affordable and feasible solution to manage the regional risks of depression.

## 4. Discussion

This study contributes real-world observational evidence suggesting that the implementation of a low-cost primary psychological health care policy may be a potential way in managing the regional risks of depression in children/adolescents. We found an incidence of 13.0% for depression among children/adolescents in the entire lower-middle-economic-status city, and further revealed nearly twofold incidence and RRs of depression in those with underprivileged conditions compared to typically developing ones. Following the implementation of such a low-cost ($1.6 per one) primary psychological health care policy, we observed that the incidence and RR of depression in this cohort significantly decreased by nearly 50%. This improvement was observed irrespective of whether children/adolescents were exposed to underprivileged conditions or not, with some reductions in the risk of depression for underprivileged individuals lasting at least 1 year. In summary, implementing the primary psychological health care system may serve as an equal, affordable, and feasible solution for LMICs or lower-middle-economic-status areas in managing the regional risks of depression for such vulnerable populations.

In contrast with existing reports [7, 47], we found a slightly lower incidence of depression in children/adolescents (13.0%) in this city. While higher incidence rates of depression were reported frequently, the lack of transparent information regarding working pipelines derived from multifarious measurements may be the main source of such heterogeneous results [48]. To address such disparities, it has been increasingly argued to build a unified, standardized, and reliable measure [49–51]. The WHO, which is jointly supported by UN Women and the Children's Fund, has constituted Global Action for Measurement of Adolescent health (GAMA) Advisory Group to develop reliable adolescent-specific measures on mental health [27, 52], whilst such initiatives have stalled in LMICs. Thus, the conclusion of this study may be worthy to be refined by benefits from better measures aligned to these children/adolescents in the future.

As far as we know, this study may provide the first empirical observations supporting the potential real-world advantages of a low-cost primary psychological health care policy in managing the risks of depression, showing an approximate 50% RRR among underprivileged children/adolescents. While integrating mental health into primary health care policies has been widely believed to improve accessibility to psychological services for those in need and subsequently reduce the risk of depression [53, 54], there remains limited understanding of how these substantial financial investments and ambitious policies influence real-world depression risk reduction among vulnerable populations. Recent empirical evidence suggests that incorporating depression screening into the pediatric primary health care policy improves recognition and treatment rates for children/adolescents but also highlights the generalizability limits and variability of such policies, particularly in rural and low-income areas, because such policies do not encompass more comprehensive and accessible psychological services following the initial screening [55, 56]. In contrast to simpler integration into pediatric care, higher-income areas (e.g., Australia) have formulated more sophisticated and domain-specific primary mental health policies with varying effectiveness by geography and ethnic subgroups [57, 58]. To address these variations in the primary care policies, a new health care model titled “collaborative care intervention”, has been established, and is further manifested to effectively and robustly mitigate children depression, which includes one-round in person engagement and multiround follow-ups (akin to “1 + n” model) [59, 60]. However, such benefits are still questioned due to troubling public health economic burdens, with limited performance in underprivileged populations [61, 62]. Thus, rather than addressing contexts in developed countries or wealthier cohorts, our study reveals the practical feasibility of a low-cost health care policy in managing regional depression risks among children and adolescents in lower-middle-income areas, contributing insights on the challenges of generalizing this primary psychological health care model to broader “third-world” contexts with economic limitations.

Another major challenge to implementing such a policy globally is the intangible public economic burdens. Despite the existence of nonprofit medical activities, these mental health care practices included in the primary psychological health care policy are highly dependent on numerous prerequisites, such as medical infrastructure, family education, cultural development, and technology-enabled integrated systems, all of which are underdeveloped or unaffordable in lower-middle-income areas [63, 64]. To strengthen the applicability and feasibility of implementing such health care policy in developing countries, the “2 + 2” model in the CPHG practice was tailored to optimize expenditures by encouraging early health care access for “at-risk” children/adolescents. Evidence from real-world data suggests that emphasizing early intervention in policy design can reduce financial costs and alleviate public health burdens [65–67]. Taken together, this policy, incorporating the “2 + 2” model for low-cost primary psychological health care, provides a potentially valuable framework for managing depression in children/adolescents, with distinct advantages in lowering public health burdens in these lower-middle-income areas.

Despite apparent strengths, some challenges are not addressed well. The generalization of this conclusion to other lower-middle-economic-status LMICs or areas is still unclear, because it draws from this single pilot site (Nanchong). Given the policy changes due to the COVID-19 pandemic in China, almost half of the participants in the original sample were lost in follow-ups. Though we found no statistically significant sociodemographic heterogeneity between this follow-up sample and the original one, this may unavoidably raise risks of bias in the sampling. Relating to this factor, we cannot accurately model the unpredictable impacts of the COVID-19 pandemic when estimating the “pure” effectiveness of implementing this health care policy in reducing the risks of depression, especially in the context of policy changes, such as lifting lockdowns during follow-up with these children and adolescents. Moreover, to protect children/adolescents from mental health problems during the pandemic, online mental health education had been undertaken for their parents by local public health authorities. Although these are not psychological interventions, it is unclear whether the effects of implementing this primary psychological health care are somewhat confounded by these makeshift public health measures. Despite the multicenter nature of the study, the school-based health care centers significantly dominated this sample. The homogeneity of these health care centers should be carefully considered when extending our conclusions elsewhere. Given the ethical restrictions, we were unable to utilize a randomized controlled trial (RCT) design or establish a control group, resulting in a single-arm study that cannot establish causality and only identifies associations between policy implementation and reduced depression incidence. Therefore, we do not purport to isolate the specific contribution of this policy in reducing the high incidence of depression among children and adolescents. Rather, we present a low-cost, scalable primary mental health care strategy as a feasible and practical intervention for resource-limited settings, with the potential to mitigate rising youth mental health burdens. Moreover, while we anticipate the affordability and feasibility of this low-cost (averaged $1.6 per child/adolescent) health care in benefiting those in LMIC, this model is still oversimplified given the unpredictable expenditure on infrastructure, health education, and expert resources.

In conclusion, this may be the first large-scale observational cohort study to show the twofold incidence/risks of depression in underrepresented children/adolescents with underprivileged conditions compared to typically developing cohorts, peaking at girls aged 12–18 years. More importantly, we observed a 50% decrease in the incidence/risks of depression for these underprivileged children/adolescents after implementing a primary psychological health care policy incorporated “2 + 2” model over a 1-year period. Thus, we provide primary real-world evidence suggesting that implementing a low-cost primary psychological health care policy may yield significant benefits and serve as a practical, affordable, and broadly applicable approach for addressing the regional risks of depression in underprivileged children and adolescents, particularly in areas with lower-middle-economic status.

## Figures and Tables

**Table 1:** Sociodemographic characteristics of the population that enrolled at the baseline in this study.

Sociodemographic characteristics	Typically developing cohort (*n* = 40,467)	De facto unattended cohort (*n* = 2444)	Orphan cohort (*n* = 762)	Children in especially difficult circumstance cohort (*n* = 18,419)	“Left-behind” cohort (*n* = 179,877)	“Single-parent” cohort (*n* = 48,270)
Age (years)	11 (10–13)	13 (11–16)	13 (11–15)	12 (10–14)	13 (11–15)	13 (11–15)
Sex						
Female	19,682 (48.6%)	1209 (49.3%)	339 (44.4%)	8467 (45.9%)	87,625 (48.6%)	24,354 (50.4%)
Male	20,785 (51.4%)	1235 (50.7%)	423 (55.6%)	9952 (54.1%)	92,252 (51.4%)	23,916 (49.6%)
Living areas						
Urban	25,231 (62.3%)	804 (32.8%)	193 (24.3%)	5660 (30.6%)	68,559 (38.0%)	19,752 (40.8%)
Rural	15,236 (37.7%)	1640 (67.2%)	569 (75.7%)	12,759 (69.4%)	111,318 (62.0%)	28,518 (59.2%)
Number of offspring						
One	5891 (14.5%)	630 (25.8%)	330 (44.3%)	3361 (18.2%)	33,716 (18.7%)	16,783 (34.7%)
More than one	34,576 (85.5%)	1814 (74.2%)	432 (55.7%)	15,058 (81.8%)	146,161 (81.3%)	31,487 (65.3%)
Family economic status						
Poor	0 (0%)	—	—	18,419 (100%)	12,270 (6.8%)	4574 (9.4%)
Middle	30,673 (75.8%)	—	—	0 (0%)	56,114 (31.1%)	13,569 (28.0%)
Upper–middle	9586 (23.6%)	—	—	0 (0%)	12,858 (7.2%)	3005 (6.2%)
Rich	208 (0.5%)	—	—	0 (0%)	181 (0.1%)	59 (0.1%)
Unwilling to answer	0 (0%)	—	-·	0 (0%)	99,454 (54.8%)	27,063 (56.3%)
Family subjective ES						
Always satisfied	3899 (9.7%)	—	—	2028 (10.9%)	98,698 (54.9%)	27,440 (56.9%)
Sometime satisfied	318 (0.7%)	—	—	29 (0.1%)	6086 (3.3%)	1262 (2.6%)
Unsatisfied	85 (0.3%)	—	—	4 (<0.1%)	1038 (0.6%)	311 (0.6%)
Unwilling to answer	36,165 (89.3%)	—	—	16,358 (88.8%)	74,055 (41.2%)	19,257 (39.9%)

*Note:* Data are *n/N* or median (IQR). Data were extracted from CPHG group across 596 sites, which covered the almost all the areas of Nanchong city (Sichuan, China). A total of 298,623 children/adolescents were included in the current study at the baseline (December 30, 2022), whereas 7249 children/adolescents were excluded because they are unable to be categorized into neither one. Questions relating to family status were omitted for all the children/adolescents living outside family (i.e., de facto unattended and orphan cohorts). Family economic statuses are categorized by national criterion that made by National Bureau of Statistics in the China, with family income per year (FIY) <60,000 CNY for poor, and with 60,000 ≤ FIY < 150,000 for middle economic level, and with 150,000 ≤ FIY < 30,0000 for upper–middle economic level, and with FIY ≥ 300,000 for rich income level.

Abbreviation: ES, economic status.

## Data Availability

All the follow-up data had been deposited into the Science Data Bank (ScienceDB, https://doi.org/10.57760/sciencedb.12150) for full accesses once being approved by the Data Regulation Office (CPHG-DRO). Form for applying the case-by-case approval could be found at the FigShare (https://doi.org/10.6084/m9.figshare.24297532.v1). All the analyses were implemented by the commercial software (i.e., SPSS, IBM.Inc.) and open-source R packages (e.g., MICE). No customized codes or scripts were used in the present study.

## Supporting Information

Supporting Information 1 The workflow diagram for the “2 + 2 model” primary psychological health care.

Supporting Information 2 The supplemental methods and results of the current study.

Supporting Information 3 The technical book of the Psychological Health Guard for Children and Adolescents Project of China (CPHG).
