# Supplementary material for: Implementation of Primary Psychological Healthcare Policy to Address the Risk of Depression in Underprivileged Children and Adolescents, in the Entire Lower-Middle-Economic-Status City of China: An Observational, Multicenter, and Single-Arm Cohort Study
Source: Depress Anxiety. 2025 Sep 11;2025:5572365. doi: 10.1155/da/5572365 (PMC12446603; doi:10.1155/da/5572365)
Supplement: Supporting Information 2 — The supplemental methods and results of the current study. [file 5572365.f2.docx]

**Supplemental Information 2**

**Title: Implementation of primary psychological healthcare policy to address the risk of depression in underprivileged children and adolescents, in the entire lower-middle-economic-status city of China: An observational, multicenter, and single-arm cohort study**

**Running title:** Primary psychological healthcare in underprivileged child/adolescent

**Authors:** Wei Li ^1*^
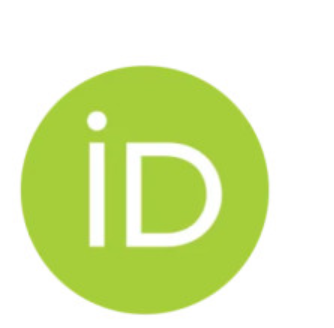
, MBBS, Xuerong Liu ^1*^
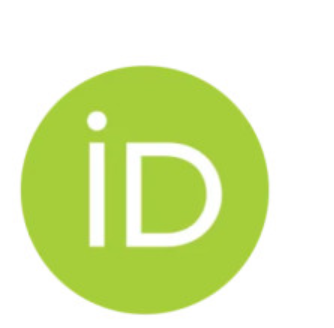
, Msc, Qianyu Zhang ^1,2^
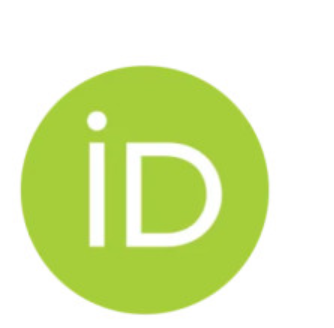
, Msc, Lei Xia ^1^
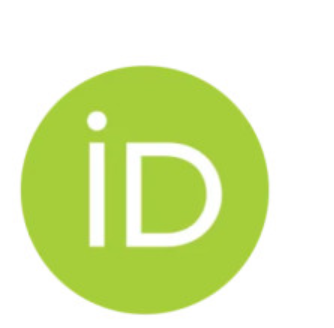
, Msc, Yanyan Li ^1^, Msc, Xiaobing Tian ^3,4^, MD, Jie Gong ^4^, Msc, Jidong Ren ^4^**✉**
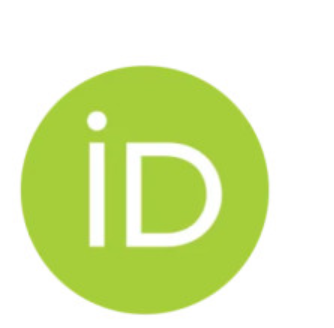
, MD, Chang Shen ^1,2^, BS, Yi Wu ^1^, BS, Ji Chen ^5^
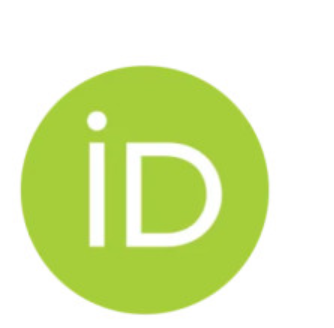
, PhD, Hu Chuan-Peng ^6^
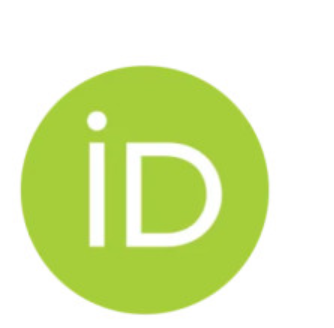
, PhD, Jing-Xuan Zhang ^1^ PhD, Ting Xu ^7^
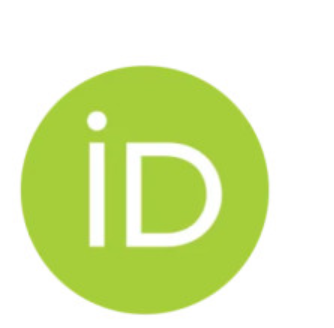
, Msc, Yuanyuan Hu ^8^
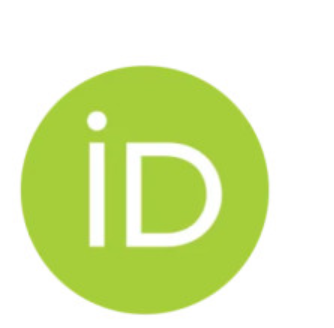
Msc, Bowen Hu ^9^
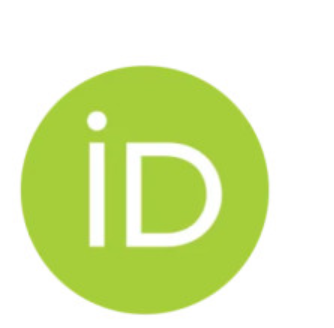
Msc, Ni Yan ^10^
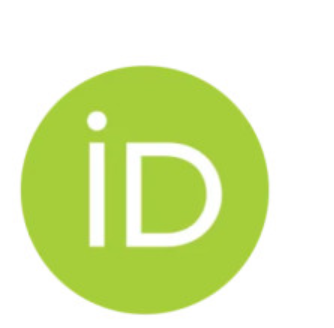
PhD, Tingyong Feng ^10^**✉**
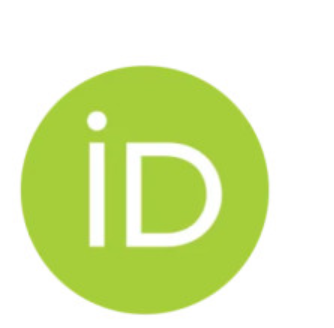
PhD, Zhengzhi Feng ^1^**✉**
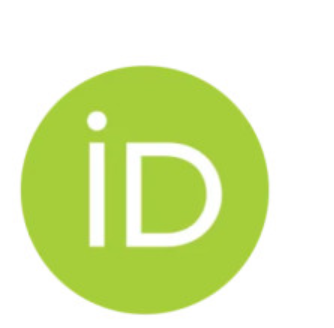
, MD, Zhiyi Chen ^1,10*^**✉**
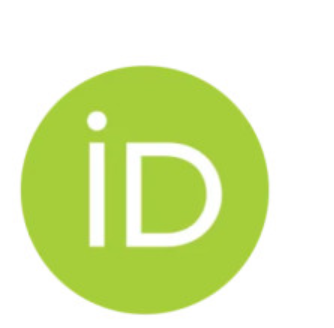
, PhD, on behalf of the Psychological Health Guard for Children and Adolescents Project of China (CPHG) Group

**Affiliation:**

^1^ Experimental Research Center of Medical and Psychological Science (ERC-MPS), School of Psychology, Third Military Medical University, Chongqing, China 400038

^2^ Department of Public Management, Chongqing University, Chongqing, China 400044

^3^ Department of Epidemiology and Public Health Statistics, North Sichuan Medical College, Nanchong, Sichuan, China 637000

^4^ Nanchong Psychosomatic Hospital (The Sixth People's Hospital of Nanchong), Sichuan, China 637000

^5^ Department of Psychology and Behavioral Sciences, Zhejiang University, Hangzhou, Zhejiang, China 310058

^6^ School of Psychology, Nanjing Normal University, Nanjing, China 518872

^7^ The Clinical Hospital of Chengdu Brain Science Institute, MOE Key Laboratory for Neuroinformation, University of Electronic Science and Technology of China, Chengdu, Sichuan China 310018

^8^ The Institute of Psychology, Chinese Academy of Sciences, Beijing, China 100864

^9^ School of Psychology, Beijing Normal University, Beijing, China 100091

^10^ Key Laboratory of Cognition and Personality, Ministry of Education, Faculty of Psychology, Southwest University, Chongqing, China 400715

✉ Corresponding at: Zhiyi Chen [(chenzhiyi@tmmu.edu.cn](mailto:(chenzhiyi@tmmu.edu.cn); TEL: +86 0 68771767) or Zhengzhi Feng [(fzz@tmmu.edu.cn; TEL: +86 0 68771480),](mailto:(fzz@tmmu.edu.cn),) Experimental Research Center for Medical and Psychological Science, School of Psychology, Third Military Medical University, Chongqing, P.R. China; Tingyong Feng [(fengty0@swu.edu.cn](mailto:(fengty0@swu.edu.cn); TEL: +86 23 68367572), School of Psychology, Southwest University, Chongqing, P.R. China; Jidong Ren [(rjd1226@163.com](mailto:(rjd1226@163.com)), Nanchong Psychosomatic Hospital (The Sixth People's Hospital of Nanchong), Sichuan, P.R. China

* contributed equally

**Summary:**

4 of 4 Supplementary Methods

10 of 10 Supplementary Results

2 of 2 Supplementary Color Figures

48 of 48 Supplementary Tables

**CONTENT**

**Supplemental Methods .............................................................................................. 1**

1. **Benchmarks of legally mandated underprivileged conditions ................................. 1**
2. **Measures for screening depressive symptoms .............................................................. 2**

**Center for Epidemiological Studies-Depression Scale (CES-D) .............................................. 2**

**Self-rating Depression Scale (SDS) ...................................................................... 2**

1. **Psychological health-care centers ............................................................................. 3**
2. **Statistics ................................................................................................................. 4**

**Log-binomial regression model ............................................................................ 4**

**McNemar paired two-sample test .................................................................................. 4**

**Relative Risk Reduction (RRR) and Absolute Risk Reduction ............................................... 4**

**Noninferiority tests .................................................................................................... 5**

**Dataset of China Family Panel Studies (CFPS) .................................................................... 5**

**Supplemental Results .............................................................................................................. 6**

1. **Overall incidence rates at the baseline (T1) .............................................................. 7**
2. **Sex-specific and age-specific incidence rates at the baseline (T1) ....................................... 8**
3. **Relative risk (RR) of suffering from depression in the underprivileged conditions (T1) ........................................................................................................................................ 14**
4. **Results of follow-up investigations for primary psychological healthcare ........................... 16**

**Demographic information ........................................................................................... 16**

**Decreases in the incidence of depression after primary psychologica healthcare .............. 17**

**Sex-specific and age-specific changes of incidence rates after primary psychological healthcare .............................................................................................................................. 19**

1. **Robustness analysis at the half-year follow-up .......................................................... 38**
2. **Results of noninferiority tests .................................................................................. 45**
3. **Results of Cochran-Armitage tests.................................................................................... 45**
4. **The difference on depression trajectory between children/adolescents with and without the two-round early psychological interventions…………………………………………………………………………..46**
5. **Public budget statement .................................................................................... 48**
6. **STROBE statement .................................................................................................. 50**

**Supplemental Methods**

1. **Benchmarks of legally mandated underprivileged conditions**

According to benchmarks that made up by Ministry of Civil Affairs of the People’s Republic of China, there are five categories to be required for legally mandated social care, including de facto unattended children/adolescents (dfUCA), orphan, children/adolescents in especially difficult circumstance (CEDC), “left-behind” and “single-parent” children/adolescents. We shall introduce these benchmarks to identify specific underprivileged conditions by restructuring into point-by-point framework. Besides to “left-behind” children/adolescents, these cohorts that requiring legally mandated social care have been registered into the local social care digital system (see following box).

| **De facto unattended children/adolescents^1^** |
| --- |
| Both parents meet the criteria of severe disability, severe illness, serving a prison sentence, mandatory isolation for drug rehabilitation, subject to other measures restricting personal freedom, missing, revoked guardianship qualifications, or being deported (expelled) from the country; Or one parent has passed away or gone missing, while the other parent meets the criteria of severe disability, severe illness, serving a prison sentence, mandatory isolation for drug rehabilitation, subject to other measures restricting personal freedom, missing, revoked guardianship qualifications, or being deported (expelled) from the country. |
| **Orphan^2^** |
| Unmarried adolescents aged less than 18 years who lost both parents;  Or Unmarried adolescents aged less than 18 years who cannot reach out/locate to biological parents. |
| **Children/adolescents in especially difficult circumstance (CEDC)^3^** |
| Child/adolescent who living in family containing a main member suffering from old age, physical weakness, living alone without care, or loss of working capacity;  Or child/adolescent who living in family with less than $ 8206 income per year (gathered from all the family members). They are entitled by the term of “Wubao”, requiring a legally mandated social care for five aspects, including essentially material supports, medical care, housing security, mental care, and funeral expenditure. |
| **The “left-behind” children/adolescents^4^** |
| Child/Adolescent who is left in the home without parenting care because parents (or either one) working away from home;  Or child/adolescent who lacked parents’ care in the home because they work too long to be unable for guardianship. |
| **The “single-parent” children/adolescents^5^** |
| Child/Adolescent who is parented by either (biological) father or (biological) mother solely;  Or Child/Adolescent who is parented by only one parent once her/his (biological) parent is unable for bringing up, as results of death, divorce or abandon. |

These children/adolescents with underprivileged conditions have been categorized into one condition that defined above, at least. This process has been done by being aided from local social-care authorities. A portion of children/adolescents who are unclear for which condition should be identified are removed from this study. Furthermore, children/adolescents who are identified to overlap across these conditions are excluded from this study as well. Despite exclusive in data analysis, all the children/adolescents have undergone primary psychological healthcare.

1. **Measures for screening depressive symptoms**

**Center for Epidemiological Studies-Depression Scale (CES-D)**

The CES-D is one of the widely-used self-reported scale for screening depressive experiences in the large-scale epidemiological investigation, showing well reliability and validity in the Chinese population.^6^ This is scaled by four-point style to describe the intensity of experiencing depressive feelings within 1 week, with “0” for “no experience”, “1” for “rare”, “2” for “frequent” and “3” for “almost all the time”. To strengthen readability and understandings for children/adolescents, the descriptions for some items have been slightly modified, whilst all the items with reverse descriptions have been rephrased to be kept consistent with other items **(Tab. S1)**.

| **Item** |
| --- |
| My appetite was poor |
| I could not shark of the blues despite much supports from family and friends |
| I had trouble keeping my mind on what I was doing |
| I felt depressed |
| My sleep was restless |
| I felt sad |
| I could not get going |
| Nothing made me happy |
| I felt like a bad person than other ones |
| I lost interests in my life |
| I talk much less than usual |
| I had ever cried |
| I felt fear |
| I felt bad evaluations to me from other persons |
| I felt alone |
| I was tired to do anything all the time |
| I feel hopeless |
| I fail to go well for my life |
| I had a lot of trouble in many non-sense things |
| I felt no one like me |

**Table S1.** Items for the Center for Epidemiological Studies-Depression Scale (CES-D)

**Self-rating Depression Scale (SDS)**

The SDS included 20 items to identify the depressive symptom for these children/adolescents who were found depressive experiences in the last round of screening by 4-point Likert-style scoring. Likewise, a total of 10 items with reverse descriptions have been rephrased to favor understandings of these items for children/adolescents **(Tab. S2)**.^7^

| **Item** |
| --- |
| I feel down-hearted and blue |
| I have crying spells, or feel like it |
| Morning is when I feel the worst |
| I have trouble sleeping at night |
| I eat as not much as I used to |
| I notice that I am losing weight |
| I cannot enjoy when I talk with ones of the opposite sex |
| I have trouble with constipation |
| My heart beats faster than usual |
| I get tired for no reason |
| I find myself restless and can’t keep still |
| I find it hard to do the things I used to |
| My mind is as not clear as it used to be |
| My life is pretty bad |
| I feel hopeful about the future |
| I find it hard to make decisions |
| I am more irritable than usual |
| I lost to enjoy the things I used to |
| I feel that I am not useful and needed |
| I feel that others would be better off if I were dead |

**Table S2.** Items for the self-rating depression scale (SDS)

1. **Psychological health-care center (s)**

In the current study, these children/adolescents who were recommended to transfer for clinical diagnosis and treatments have undergone such cares in government-sponsored mental health centers (hospitals). Details for these centers have been detailed in **Tab S3**.

| **Regions** | **Centers (hospitals)** | **Class** | **Category** |
| --- | --- | --- | --- |
| Pengan | Nanchong City Physical and Mental Hospital | Grade III Level B hospital | public |
|  | Affiliated Hospital of North Sichuan Medical College | Grade III Level A hospital | public |
| Yilong | Yilong County People's Hospital | Grade III Level B hospital | public |
|  | Nanchong Central Hospital | Grade III Level A hospital | public |
| Langzhong | Langzhong People's Hospital | Grade III Level A hospital | public |
|  | Nanchong Central Hospital | Grade III Level A hospital | public |
| Gaoping | Nanchong City Physical and Mental Hospital | Grade III Level B hospital | public |
| Jialing | Nanchong City Physical and Mental Hospital | Grade III Level B hospital | public |
|  | Nanchong Second People's Hospital | Grade III Level A hospital | public |
| Nanbu | Nanbu County People's Hospital | Grade III Level A hospital | public |
| Yingshan | Nanchong City Physical and Mental Hospital | Grade III Level B hospital | public |
| Xichong | Affiliated Hospital of North Sichuan Medical College | Grade III Level A hospital | public |
|  | Nanchong Central Hospital | Grade III Level A hospital | public |

**Table S3.** Details for center (hospital) to provide medical treatment/service for these children/adolescents

1. **Statistics**

**Log-binomial regression model**

Given the > 10% incidence of depression in these children/adolescents, we built upon the log-binomial regression model to minimize standard error in predicting the roles of exposing to underprivileged conditions on the relative risk (RR) of depression, compared to logistic model^8,9^:

$$ln(P) = \beta_{0}+\beta_{1}\mathrm{Expose}_{1}+\beta_{2}\mathrm{Age}_{2}+\beta_{3}\mathrm{Sex}_{3}+\beta_{4}\mathrm{Centers}_{4}+\beta_{5}{Living areas}_{5}+\beta_{6}{No. of offspring}_{6}$$

Here, the dependent variable (DV) was estimated to the RR of exposing to underprivileged conditions or not (independent variable, IV). Age, sex, centers, living areas and the total number of offspring have been modeled as confounders for adjustments. These estimates have been done by “lme”package of R (4.2.1).

**McNemar paired two-sample test**

We built up the paired 2 x 2 crosstables to count the frequency of depression for all the children/adolescents before and after implementing the primary psychological healthcare policy, and further estimated statistical significance by McNemar test, with continuing corrections in a *X*^2^ distribution.^10,11^ The Bonferroni-Holm correction was used to adjust p values for controlling the potential inflation of false-positive error.

**Relative Risk Reduction (RRR), Absolute Risk Reduction (ARR) and Number Needed to Treat (NTT)**

To estimate the extent to which the primary psychological healthcare policy in decreasing the risk of depression for these children/adolescents, we used RRR for each cohort as scalar. The RRR was calculated by the following equation:

$$RRR= \frac{incidence of depression (before) - incidence of depression (after)}{(incidence of depression (before)}\times100\%$$

Here, the incidence of depression before the implementation of primary psychological healthcare policy was calculated as benchmark to estimate “relative” risk reduction by decreased incidence of depression after this policy. It should bear in mind that the RRR is not specialized for such within-subject design. Thus, the RRR may be inflated as the within-subject autocorrelation.^12,13^ To obviate risks of inflating effect size, we also calculated the Absolute Risk Reduction (ARR) and Number Needed to Treat (NTT). ARR was quantified by the absolute difference between before-healthcare and after-healthcare incidence of depression. NNT was calculated as the reciprocal of ARR, representing the number of children and adolescents who would need to receive the psychological healthcare services.

**Noninferiority tests**

To determine whether the change in the incidence/risk of depression among children and adolescents in underprivileged conditions was noninferior to that observed in typically developing peers, the noninferiority tests were carried on. We estimated the sample size to provide 95% statistical power for these noninferiory tests, with the assumption of a RRR of 40%. Given no adequate information for the noninferiority margins, an interval ranged from 5% to 20% was used, with iterations for each percentage point. The final significance level of 0.025 was set on two-side assumption. By using normal approximation method, minimum sample size was found to be 1146 in the noninferiority tests, with considerations for 10% dropouts of the whole sample.^14^ The two-sample ratio Z tests were used for statistical inferences on these noninferiority models.

**Dataset of China Family Panel Studies (CFPS)**

As an observational control group, we included a large-scale longitudinal dataset drawn from China Family Panel Studies (CFPS), with over 14-year follow-ups for 42, 590 Chinese peoples from 14, 961 families since 2010, at the 2-year interval for follow-up investigations (more details can be found at www.isss.pku.edu.cn/cfps/). Children/adolescents who were successfully followed for measuring depressive symptoms had been enrolled as an observational control group (*n* = 730), at T1 (2016), T2 (2018), T3 (2020). No data have been collected for depressive symptoms in the follow-ups at 2010, 2012 and 2014 in the CFPS project. Depressive symptoms were measured by the short-version Center for Epidemiological Studies-Depression Scale (CES-D), and were categorized into “identified depression group” and “healthy group” by the cut-off point at total scores of ≥ 7. More details for the data collection and measurements can be found from the technical handbook of the CFPS (https://doi.org/10.18170/DVN/45LCSO). Here, this CFPS sample was manually redrawn from a local publication (i.e., 10.16835/j.cnki.1000-9817.2024005). The Cochran-Armitage test with equal-distance weights (addressing imbalances of time intervals, e.g., 2-year for CFPS dataset and 0.5-year for the current sample), was used to examine the monotonically-assumption trends for incidence rates of identifying depression for these children/adolescents across three follow-ups.

**SUPPLEMENTAL RESULTS**

1. **Overall incidence rates**

We estimated the overall incidence rates of depression in these underprivileged cohorts, plus the typically developing cohort. Details have been shown in the **Tab S5**.

| **Cohort** | ***No.* depressed** | ***N*, total** | **Incidence, %** | **95% CI, %** |
| --- | --- | --- | --- | --- |
| Typically developing cohort | 3024 | 40467 | 7.5 | 7.2-7.7 |
| dfUCA | 495 | 2444 | 20.3 | 18.7-21.8 |
| Orphan | 154 | 762 | 20.2 | 17.4-23.1 |
| CEDC | 2367 | 18419 | 12.8 | 12.4-13.3 |
| “left-behind” | 23450 | 179877 | 13.0 | 12.9-13.2 |
| “single-parents” | 8233 | 48270 | 17.1 | 16.7-17.4 |
| **Entire underprivileged cohort** | 34699 | 249772 | 13.9 | 13.7-14.1 |
| **Overall** | 37723 | 290239 | 13.0 | 12.9-13.1 |

**Table S5.** Incidence rates of identifying depression in each cohort, full names for abbreviations can be found at the supplemental results 1

1. **Sex-specific and age-specific incidence rates**

We further clarified the specific incidence rates of depression in these underprivileged conditions in gender-specific and age-specific statistics. Results for sex-specific incidence rates in detecting depression have been detailed at the **Tab. S6**. The age ranges were partitioned into five groups, including 6-8 years, 9-11 years, 12-14 years, 15-17 years and 18 year. Age-specific estimates for each cohort have been sorted into **Tab S7-S12**.

| **Cohorts** | **Boys, *n*** | **Girls, *n*** | **Mild Symptom** | | | | **Moderate Symptom** | | | | **Severe Symptom** | | | |
| --- | --- | --- | --- | --- | --- | --- | --- | --- | --- | --- | --- | --- | --- | --- |
|  |  |  | **Boy, *n* of identifying depression** | **Incidence rates, % (95% CI)** | **Girl, *n* of identifying depression** | **Incidence rates, % (95% CI)** | **Boy, *n* of identifying depression** | **Incidence rates, % (95% CI)** | **Girl, *n* of identifying depression** | **Incidence rates, % (95% CI)** | **Boy, *n* of identifying depression** | **Incidence rates, % (95% CI)** | **Girl, *n* of identifying depression** | **Incidence rates, % (95% CI)** |
| Typically developing cohort | 20785 | 19682 | 534 | 2.6  (2.4-2.8) | 710 | 3.6  (3.3-3.9) | 543 | 2.6  (2.4-2.8) | 926 | 4.7  (4.4-5.0) | 77 | 0.4  (0.3-0.5) | 234 | 1.2(1.0-1.3) |
| dfUCA | 1235 | 1209 | 72 | 5.8  (4.5-7.1) | 103 | 8.5  (6.9-10.1) | 118 | 9.6  (7.9-11.2) | 134 | 11.1  (9.3-12.9) | 13 | 1.1  (0.5-1.6) | 55 | 4.5  (3.4-5.7) |
| Orphan | 423 | 339 | 23 | 5.4  (3.3-7.6) | 24 | 7.1  (4.3-9.8) | 39 | 9.2  (6.5-12.0) | 49 | 14.5  (10.7-18.2) | 5 | 1.2  (0.2-2.2) | 14 | 4.1  (2.0-6.2) |
| CEDC | 9952 | 8467 | 417 | 4.2  (3.8-4.6) | 466 | 5.5  (5.0-6.0) | 549 | 5.5  (5.1-6.0) | 676 | 8.0  (7.4-8.6) | 71 | 0.7  (0.5-0.9) | 188 | 2.2  (1.9-2.5) |
| “left-  Behind” | 92252 | 87625 | 3678 | 4.0  (3.9-4.1) | 5222 | 6.0  (5.8-6.1) | 4659 | 5.1  (4.9-5.2) | 7339 | 8.4  (8.2-8.6) | 549 | 0.6  (0.5-0.6) | 2003 | 2.3  (2.2-2.4) |
| “single-  Parents” | 23916 | 24354 | 1210 | 5.1  (4.8-5.3) | 1728 | 7.1  (6.8-7.4) | 1659 | 6.9  (6.6-7.3) | 2629 | 10.8  (10.4-11.2) | 193 | 0.8  (0.7-0.9) | 814 | 3.3  (3.1-3.6) |
| **Entire underprivileged cohort** | 127752 | 122020 | 5400 | 4.2  (4.1-4.3) | 7543 | 6.2  (6.0-6.3) | 7024 | 5.5  (5.4-5.6) | 10827 | 8.9  (8.7-9.0) | 831 | 0.6  (0.6-0.7) | 3074 | 2.5  (2.4-2.6) |
| **Overall** | 148537 | 141702 | 5934 | 4.0  (3.9-4.1) | 8253 | 5.8  (5.7-5.9) | 7567 | 5.1  (5.0-5.2) | 11753 | 8.3  (8.1-8.4) | 908 | 0.6  (0.5-0.6) | 3308 | 2.3  (2.3-2.4) |

**Table S6.** Sex-specific Incidence rates of identifying depression symptom (at different level) in each cohort, full names for abbreviations can be found at the supplemental results 1

|  | **Mild Symptom** | | | | **Moderate Symptom** | | | | **Severe Symptom** | | | |
| --- | --- | --- | --- | --- | --- | --- | --- | --- | --- | --- | --- | --- |
|  | **Boy, *n* of identifying depression** | **Incidence rates, % (95% CI)** | **Girl, *n* of identifying depression** | **Incidence rates, % (95% CI)** | **Boy, *n* of identifying depression** | **Incidence rates, % (95% CI)** | **Girl, *n* of identifying depression** | **Incidence rates, % (95% CI)** | **Boy, *n* of identifying depression** | **Incidence rates, % (95% CI)** | **Girl, *n* of identifying depression** | **Incidence rates, % (95% CI)** |
| **6-8** | — | — | — | — | 1 | 1.8 (-1.7-5.3) | 1 | 0.9 (-0.8-2.6) | — | — | — | — |
| **9-11** | 137 | 1.2 (1.0 - 1.4) | 131 | 1.3 (1.0 - 1.5) | 124 | 1.1 (0.9 - 1.3) | 161 | 1.5 (1.3 - 1.8) | 19 | 0.2 (0.1 - 0.2) | 35 | 0.3(0.2 - 0.4) |
| **12-14** | 218 | 3.3 (2.9 - 3.7) | 335 | 5.4 (4.9 - 6.0) | 221 | 3.3 (2.9 - 3.8) | 443 | 7.2 (6.5 - 7.8) | 33 | 0.5 (0.3 - 0.7) | 120 | 1.9(1.6 -2.3) |
| **15-17** | 164 | 5.8 (5.0 - 6.7) | 237 | 8.3 (7.3 - 9.3) | 183 | 6.5 (5.6 - 7.4) | 306 | 10.8 (9.6 - 11.9) | 21 | 0.7 (0.4 - 1.1) | 75 | 2.6(2.0 - 3.2) |
| **18** | 15 | 7.8 (4.0 - 1.5) | 7 | 5.3 (1.5 - 9.1) | 14 | 7.3 (3.6 - 10.9) | 15 | 11.3 (5.9 - 16.7) | 4 | 2.1 (0.1 - 4.1) | 4 | 3.0 (0.1 - 5.9) |

**Table S7.** Sex-specific Incidence rates of identifying depression symptom (at different level) in typically developing cohort, full names for abbreviations can be found at the supplemental results 1, “-” indicates no applicable data are to be shown here

|  | **Mild Symptom** | | | | **Moderate Symptom** | | | | **Severe Symptom** | | | |
| --- | --- | --- | --- | --- | --- | --- | --- | --- | --- | --- | --- | --- |
|  | **Boy, *n* of identifying depression** | **Incidence rates, % (95% CI)** | **Girl, *n* of identifying depression** | **Incidence rates, % (95% CI)** | **Boy, *n* of identifying depression** | **Incidence rates, % (95% CI)** | **Girl, *n* of identifying depression** | **Incidence rates, % (95% CI)** | **Boy, *n* of identifying depression** | **Incidence rates, % (95% CI)** | **Girl, *n* of identifying depression** | **Incidence rates, % (95% CI)** |
| **6-8** | — | — | — | — | — | — | — | — | — | — | — | — |
| **9-11** | 13 | 3.5 (1.6 - 5.4) | 18 | 5.3 (2.9 - 7.6) | 20 | 5.4 (3.1 - 7.7) | 15 | 4.4 (2.2 - 6.6) | — | — | 8 | 2.3 (0.7 - 3.9) |
| **12-14** | 19 | 4.8 (2.7 - 6.9) | 34 | 8.1 (5.5 - 10.7) | 33 | 8.3 (5.6 - 11.1) | 57 | 13.5 (10.3 - 16.8) | 5 | 1.3 (0.2 - 2.4) | 20 | 4.8 (2.7 - 6.8) |
| **15-17** | 32 | 7.8 (5.2 - 10.4) | 50 | 12.0 (8.9 - 15.2) | 55 | 13.4 (10.1 - 16.7) | 57 | 13.7 (10.4 - 17.0) | 8 | 2.0 (0.6 - 3.3) | 25 | 6.0 (3.7 - 8.3) |
| **18** | 8 | 14.8 (5.3 - 24.3) | 1 | 3.8 (-3.5 - 11.2) | 10 | 18.5 (8.2 - 28.9) | 5 | 19.2 (4.1 - 34.4) | — | — | 2 | 7.7 (-2.6 - 17.9) |

**Table S8.** Sex-specific Incidence rates of identifying depression symptom (at different level) in the de facto unattended children/adolescents cohort, full names for abbreviations can be found at the supplemental results 1, “-” indicates no applicable data are to be shown here

|  | **Mild Symptom** | | | | **Moderate Symptom** | | | | **Severe Symptom** | | | |
| --- | --- | --- | --- | --- | --- | --- | --- | --- | --- | --- | --- | --- |
|  | **Boy, *n* of identifying depression** | **Incidence rates, % (95% CI)** | **Girl, *n* of identifying depression** | **Incidence rates, % (95% CI)** | **Boy, *n* of identifying depression** | **Incidence rates, % (95% CI)** | **Girl, *n* of identifying depression** | **Incidence rates, % (95% CI)** | **Boy, *n* of identifying depression** | **Incidence rates, % (95% CI)** | **Girl, *n* of identifying depression** | **Incidence rates, % (95% CI)** |
| **6-8** | — | — | — | — | — | — | — | — | — | — | — | — |
| **9-11** | 5 | 4.4 (0.6 - 8.1) | 4 | 4.4 ( 0.2 - 8.7) | 6 | 5.3 (1.2 - 9.4) | 6 | 6.7 (1.5 - 1.8) | 1 | 0.9 (-0.8 - 2.6) | 1 | 1.1 (-1.1 - 3.3) |
| **12-14** | 6 | 3.7 (0.8 - 6.7) | 7 | 4.9 (1.3 - 8.4) | 15 | 9.3 (4.8 - 13.8) | 24 | 16.7 (10.6 - 22.8) | 2 | 1.2 (-0.5 - 3.0) | 10 | 6.9 (2.8 - 11.1) |
| **15-17** | 10 | 7.7 (3.1 - 12.3) | 9 | 9.6 (3.6 - 15.5) | 17 | 13.1 (7.3 - 18.9) | 16 | 17.0 (9.4 - 24.6) | 2 | 1.5 (-0.6 - 3.7) | 3 | 3.2 (-0.4 - 6.7) |
| **18** | 2 | 11.8 (-3.6 - 27.1) | 4 | 36.4 (7.9 - 64.8) | 1 | 5.9 (-5.3 - 17.1) | 3 | 27.3 (1.0 - 53.6) | — | — | — | — |

**Table S9.** Sex-specific Incidence rates of identifying depression symptom (at different level) in the orphan cohort, full names for abbreviations can be found at the supplemental results 1, “-” indicates no applicable data are to be shown here

|  | **Mild Symptom** | | | | **Moderate Symptom** | | | | **Severe Symptom** | | | |
| --- | --- | --- | --- | --- | --- | --- | --- | --- | --- | --- | --- | --- |
|  | **Boy, *n* of identifying depression** | **Incidence rates, % (95% CI)** | **Girl, *n* of identifying depression** | **Incidence rates, % (95% CI)** | **Boy, *n* of identifying depression** | **Incidence rates, % (95% CI)** | **Girl, *n* of identifying depression** | **Incidence rates, % (95% CI)** | **Boy, *n* of identifying depression** | **Incidence rates, % (95% CI)** | **Girl, *n* of identifying depression** | **Incidence rates, % (95% CI)** |
| **6-8** | — | — | — | — | — | — | 1 | 2.4 (-2.2 - 7.0) | — | — | — | — |
| **9-11** | 111 | 2.3 (1.9 - 2.7) | 95 | 2.4 (1.9 - 2.8) | 114 | 2.4 (1.9 - 2.8) | 120 | 3.0 (2.4 - 3.5) | 17 | 0.4 (0.2 - 05) | 28 | 0.7 (0.4 - 0.9) |
| **12-14** | 137 | 4.1 (3.4 - 4.8) | 170 | 6.4 (5.5 - 7.3) | 185 | 5.5 (4.8 - 6.3) | 234 | 8.8 (7.7 - 9.9) | 27 | 0.8 (0.5 - 1.1) | 81 | 3.1 (2.4 - 3.7) |
| **15-17** | 145 | 9.0 (7.6 - 10.4) | 183 | 11.4 (9.8 - 12.9) | 226 | 14.1 (12.4 - 15.8) | 302 | 18.8 (16.9 - 20.7) | 26 | 1.6 (1.0 - 2.2) | 74 | 4.6 (3.6 - 5.6) |
| **18** | 24 | 14.1 (8.9 - 19.4) | 18 | 14.5 (8.3 - 20.7) | 24 | 14.1 (8.9 - 19.4) | 19 | 15.3 (9.0 - 21.7) | 1 | 0.6 (-0.6 - 1.7) | 5 | 4.0 (0.6 - 7.5) |

**Table S10.** Sex-specific Incidence rates of identifying depression symptom (at different level) in the children/adolescents in especially difficult circumstance (CEDC) cohort, full names for abbreviations can be found at the supplemental results 1, “-” indicates no applicable data are to be shown here

|  | **Mild Symptom** | | | | **Moderate Symptom** | | | | **Severe Symptom** | | | |
| --- | --- | --- | --- | --- | --- | --- | --- | --- | --- | --- | --- | --- |
|  | **Boy, *n* of identifying depression** | **Incidence rates, % (95% CI)** | **Girl, *n* of identifying depression** | **Incidence rates, % (95% CI)** | **Boy, *n* of identifying depression** | **Incidence rates, % (95% CI)** | **Girl, *n* of identifying depression** | **Incidence rates, % (95% CI)** | **Boy, *n* of identifying depression** | **Incidence rates, % (95% CI)** | **Girl, *n* of identifying depression** | **Incidence rates, % (95% CI)** |
| **6-8** | — | — | 4 | 1.7 (0.0 - 3.4) | — | — | 2 | 0.9 (-0.3 - 2.1) | — | — | — | — |
| **9-11** | 525 | 1.7 (1.5 - 1.8) | 677 | 2.2 (2.1 - 2.4) | 614 | 1.9 (1.8 - 2.1) | 907 | 3.0 (2.8 - 3.2) | 90 | 0.3 (0.2 - 0.4) | 258 | 0.9 (0.8 - 1.0) |
| **12-14** | 1302 | 3.8 (3.6 - 4.0) | 2120 | 6.5 (6.2 - 6.8) | 1735 | 5.1 (4.9 - 5.3) | 3197 | 9.8 (9.5 - 10.1) | 236 | 0.7 (0.6 - 0.8) | 1098 | 3.4 (3.2 - 3.6) |
| **15-17** | 1678 | 7.0 (6.7 - 7.3) | 2244 | 9.8 (9.4 - 10.2) | 2097 | 8.8 (8.4 - 9.1) | 3017 | 13.1 (12.7 - 13.6) | 214 | 0.9 (0.8 - 1.0) | 610 | 2.7 (2.4 - 2.9) |
| **18** | 173 | 7.7 (6.6 - 8.8) | 177 | 10.5 (9.0 - 11.9) | 213 | 9.5 (8.3 - 10.7) | 216 | 12.8 (11.2 - 14.3) | 9 | 0.4 (0.1 - 0.7) | 37 | 2.2 (1.5 - 2.9) |

**Table S11.** Sex-specific Incidence rates of identifying depression symptom (at different level) in the “left-behind” children/adolescent cohort, full names for abbreviations can be found at the supplemental results 1, “-” indicates no applicable data are to be shown here

|  | **Mild Symptom** | | | | **Moderate Symptom** | | | | **Severe Symptom** | | | |
| --- | --- | --- | --- | --- | --- | --- | --- | --- | --- | --- | --- | --- |
|  | **Boy, *n* of identifying depression** | **Incidence rates, % (95% CI)** | **Girl, *n* of identifying depression** | **Incidence rates, % (95% CI)** | **Boy, *n* of identifying depression** | **Incidence rates, % (95% CI)** | **Girl, *n* of identifying depression** | **Incidence rates, % (95% CI)** | **Boy, *n* of identifying depression** | **Incidence rates, % (95% CI)** | **Girl, *n* of identifying depression** | **Incidence rates, % (95% CI)** |
| **6-8** | — | — | — | — | — | — | 1 | 2.0 (-1.8 - 5.8) | — | — | — | — |
| **9-11** | 183 | 2.3 (2.0 - 2.6) | 240 | 3.0 (2.7 - 3.4) | 236 | 3.0 (2.6 - 3.3) | 360 | 4.6 (4.1 - 5.0) | 37 | 0.5 (0.3 - 0.6) | 99 | 1.3 (1.0 - 1.5) |
| **12-14** | 477 | 5.2 (4.7 - 5.6) | 747 | 7.7 (7.2 - 8.2) | 662 | 7.2 (6.6 - 7.7) | 1183 | 12.2 (11.5 - 12.8) | 84 | 0.9 (0.7 - 1.1) | 476 | 4.9 (4.5 - 5.3) |
| **15-17** | 497 | 8.1 (7.5 - 8.8) | 699 | 11.2 (10.4 - 12.0) | 689 | 11.3 (10.5 - 12.1) | 1021 | 16.3 (15.4 - 17.2) | 70 | 1.1 (0.9 - 1.4) | 230 | 3.7 (3.2 - 4.1) |
| **18** | 53 | 9.1 (6.8 - 11.5) | 42 | 9.0 (6.4 - 11.6) | 72 | 12.4 (9.7 - 15.1) | 64 | 13.8 (10.6 - 16.9) | 2 | 0.3 (-0.1 - 0.8) | 9 | 1.9 (0.7 - 3.2) |

**Table S12.** Sex-specific Incidence rates of identifying depression symptom (at different level) in the “single-parenting” children/adolescent cohort, full names for abbreviations can be found at the supplemental results 1, “-” indicates no applicable data are to be shown here

1. **Relative risk (RR) of suffering from depression in the underprivileged conditions**

We capitalized on log-binomial regression model to estimate the relative risk (RR) of identifying depression in these underprivileged cohorts compared to typically developing one, respectively. Results were provided for not only original estimates but also corrected ones that were adjusted by center, sex, living areas, the number of offspring within family and age **(Tab S13-17)**.

|  | **Estimate** | **Std.Error** | **z value** | **Pr(>\|z\|)** | **RR (95% CI)** | **Adjusted RR (95% CI)** |
| --- | --- | --- | --- | --- | --- | --- |
| Intercept | -2.5 | 0.0 | -133.1 | < 2e-16 *** | 2.9-3.4 | 2.0-2.4 |
| **dfUCA** | 1.1 | 0.1 | 21.3 | < 2e-16 *** | 3.1 (3.0-3.3) | 2.2 (2.1-2.3) |

**Table S13** Model results for the relative risk (RR) of suffering from depression in de facto unattended children/adolescents (dfUCA) than typically developing cohort

|  | **Estimate** | **Std.Error** | **z value** | **Pr(>\|z\|)** | **RR (95% CI)** | **Adjusted RR (95% CI)** |
| --- | --- | --- | --- | --- | --- | --- |
| Intercept | -2.5 | 0.0 | -133.1 | < 2e-16 *** | 2.9-3.4 | 2.0-2.4 |
| **Orphan** | 1.1 | 0.1 | 12.4 | < 2e-16 *** | 3.1(2.9-3.3) | 2.5 (2.3-2.7) |

**Table S14.** Model results for the relative risk (RR) of suffering from depression in orphan than typically developing cohort

|  | **Estimate** | **Std.Error** | **z value** | **Pr(>\|z\|)** | **RR (95% CI)** | **Adjusted RR (95% CI)** |
| --- | --- | --- | --- | --- | --- | --- |
| Intercept | -2.5 | 0.0 | -133.1 | < 2e-16 *** | 2.9-3.4 | 2.0-2.4 |
| **CEDC** | 0.6 | 0.0 | 20.8 | < 2e-16 *** | 1.8 (1.8-1.9) | 1.8 (1.8-1.9) |

**Table S15.** Model results for the relative risk (RR) of suffering from depression in children/adolescents in especially difficult circumstance (CEDC) than typically developing cohort

|  | **Estimate** | **Std.Error** | **z value** | **Pr(>\|z\|)** | **RR (95% CI)** | **Adjusted RR (95% CI)** |
| --- | --- | --- | --- | --- | --- | --- |
| Intercept | -2.5 | 0.0 | -133.1 | < 2e-16 *** | 2.9-3.4 | 2.0-2.4 |
| **left-behind** | 0.6 | 0.0 | 30.7 | < 2e-16 *** | 1.9(1.9-2.0) | 1.5 (1.4-1.5) |

**Table S16.** Model results for the relative risk (RR) of suffering from depression in “left-behind” children/adolescents than typically developing cohort

|  | **Estimate** | **Std.Error** | **z value** | **Pr(>\|z\|)** | **RR (95% CI)** | **Adjusted RR (95% CI)** |
| --- | --- | --- | --- | --- | --- | --- |
| Intercept | -2.5 | 0.0 | -133.1 | < 2e-16 *** | 2.9-3.4 | 2.0-2.4 |
| **single-parenting** | 0.9 | 0.0 | 41.6 | < 2e-16 *** | 2.5 (2.5-2.6) | 2.0 (2.0-2.1) |

**Table S17.** Model results for the relative risk (RR) of suffering from depression in “single-parent” children/adolescents than typically developing cohort

1. **Results of follow-up investigations for primary psychological healthcare policy**

**Demographic information**

A total of 148 652 children/adolescents (51.2% from the whole population) were successfully followed up at both half-year (T2) and 1-year (T3) reevaluations. Demographic information has been sorted into the **Tab S18.**

**Decreases in the incidence of depression after primary psychological healthcare policy**

To examine changes in the incidence of depression following the implementation of the primary psychological healthcare policy in these underprivileged cohorts, the paired McNemar Change test was used. In this vein, it was noteworthy that these statistics were reported by using the followed-up sample (n = 148 652), rather the whole sample in the baseline. Details for these examinations have been documented in the **Tab S19**. The relative risk reduction (RRR) was estimated for each cohort. Results have been sorted into the **Tab S20-21**.

**Sex-specific and age-specific changes of incidence rates after primary psychological healthcare**

We also provided sex-specific and age-specific incidence of depression in these children/adolescents after the primary psychological health policy into the **Tab S22-27 (Tab S28-34)** for the half-year (1-year) follow-ups. To achieve the exact estimates, the “-” was labeled to indicate “not applicable” when the total number of participants in this sex-specific and age-specific group was less than 30.

1. **Robustness analysis at the half-year follow-up**

In the half-year follow-up investigation, 72.4% children/adolescents (n = 215 441) have been followed for re-estimating their depressive symptoms. Thus, we have replicated these analysis to this follow-up sample to examine the robustness of these findings mentioned above Demographic information for this sample has been sorted into the **Tab. S35**. Details to the sex-specific and age-specific changes of incidence rates after primary psychological healthcare policy at the T2 have been tabulated into the **Tab S36-41**.

|  | **Typically developing cohort (n = 21,659)** | **De facto unattended cohort (n = 1,165)** | **Orphan cohort(n = 342)** | **Children in especially difficult circumstance cohort(n = 9,351)** | **“Left-behind” cohort(n = 92,291)** | **“Single-parent” cohort(n = 23,844)** |
| --- | --- | --- | --- | --- | --- | --- |
| **Age, years** | 11 (10 - 13) | 13 (11 - 15) | 13 (11 - 14) | 11 (10 - 13) | 12 (10 - 14) | 12 (10 - 14) |
| **Sex** |  |  |  |  |  |  |
| Female | 10,472 (48.35%) | 576 (49.44%) | 150 (43.86%) | 4,308 (46.07%) | 45,257 (49.04%) | 12,058 (50.57%) |
| Male | 11,187 (51.65%) | 589 (50.56%) | 192 (56.14%) | 5,043 (53.93%) | 47,034 (50.96%) | 11,786 (49.43%) |
| **Living Areas** |  |  |  |  |  |  |
| Urban | 13,328 (61.54%) | 337 (28.93%) | 84 (24.56%) | 2,620 (28.02%) | 34,217 (37.08%) | 9,461 (39.68%) |
| Rural | 8,331 (38.46%) | 828 (71.07%) | 258 (75.44%) | 6,731 (71.98%) | 58,074 (62.92%) | 14,383 (60.32%) |
| **No. of offspring** |  |  |  |  |  |  |
| One | 2,900 (13.39%) | 270 (23.18%) | 147 (42.98%) | 1,634 (17.47%) | 16,953 (18.37%) | 8,291 (34.77%) |
| More than one | 18,759 (86.61%) | 895 (76.82%) | 195 (57.02%) | 7,717 (82.53%) | 75,338 (81.63%) | 15,553 (65.23%) |
| **Family economic status** |  |  |  |  |  |  |
| Poor | 1 (0%) | - | - | 9,349 (99.98%) | 6,239 (6.76%) | 2,315 (9.71%) |
| Middle | 16,322 (75.36%) | - | - | 0 (0%) | 29,955 (32.46%) | 6,860 (28.77%) |
| Upper-middle | 5,218 (24.09%) | - | - | 0 (0%) | 7,119 (7.71%) | 1,618 (6.79%) |
| Rich | 118 (0.55%) | - | - | 0 (0%) | 106 (0.11%) | 31 (0.13%) |
| Unwilling to answer | 0 (0%) | - | - | 2 (0.02%) | 48,872 (52.95%) | 13,020 (54.60%) |
| **Family subjective ES** |  |  |  |  |  |  |
| Always satisfied | 18,796 (86.78%) | - | - | 6,332 (67.71%) | 71,808 (77.81%) | 15,785 (66.20%) |
| Sometime satisfied | 2,442 (11.28%) | - | - | 2,336 (24.98%) | 16,052 (17.39%) | 6,152 (25.80%) |
| Unsatisfied | 406 (1.87%) | - | - | 675 (7.22%) | 4,092 (4.43%) | 1,887 (7.92%) |
| Unwilling to answer | 15 (0.07%) | - | - | 8 (0.09%) | 339 (0.37%) | 20 (0.08%) |

**Table S18.** Sociodemographic characteristics of the population that was successfully followed at the May 21, 2023. Data are n/N or median (IQR). Data were extracted from CPHG group across 596 sites, which covered the almost all the areas of Nanchong city (Sichuan, China). Questions relating to family status were omitted for all the children/adolescents living outside family (i.e., de facto unattended and orphan cohorts). Family economic statuses are categorized by national criterion that made by National Bureau of Statistics in the China, with family income per year (FIY) < 60, 000 CNY for poor, and with 60, 000 ≤ FIY < 150, 000 for middle economic level, and with 150, 000 ≤ FIY < 30, 0000 for upper-middle economic level, and with FIY ≥ 300, 000 for rich income level. ES = economic status. Both parametric and non-parametric estimates have been conducted, and showed no statistically significant differences between this follow-up sample and the baseline sample for these sociodemographic characteristics

| **Cohort** | **Number of identifying depression at baseline (Oct 30, 2022, T1)** | **Number of identifying depression at this follow-up (May 21, 2023, T2)** | **Number of identifying depression at this follow-up (Oct 29, 2023, T3)** | **Total sample size of this cohort** | **Incidence rate at baseline (T1), %** | **Incidence rate at follow-up (T2), %** | **Incidence rate at follow-up (T3), %** | **Δ Incidence(T1-T2), %** | **Δ Incidence(T1-T3), %** | **McNemar(T1-T2), α** | **P value (two-side,T1-T2)** | **McNemar(T1-T3), α** | **P value (two-side,T1-T3)** |
| --- | --- | --- | --- | --- | --- | --- | --- | --- | --- | --- | --- | --- | --- |
| Typically developing cohort | 1321 | 865 | 1456 | 21659 | 6.1 | 4.0 | 6.7 | 2.1 | -0.6 | 129.9 | .000*** | 9.0 | .003** |
| dfUCA | 214 | 86 | 149 | 1165 | 18.4 | 7.4 | 12.8 | 11 | 5.6 | 76.8 | .000*** | 17.0 | .000*** |
| Orphan | 59 | 37 | 43 | 342 | 17.3 | 10.8 | 12.6 | 6.5 | 4.7 | 6.9 | .009** | 4.0 | .045* |
| CEDC | 982 | 447 | 900 | 9351 | 10.5 | 4.8 | 9.6 | 5.7 | 0.9 | 270.8 | .000*** | 5.0 | .025* |
| “left-behind” | 10347 | 5082 | 8101 | 92291 | 11.2 | 5.5 | 8.8 | 5.7 | 2.4 | 2531.3 | .000*** | 405.4 | .000*** |
| “single-parents” | 3550 | 1679 | 2824 | 23844 | 14.9 | 7.0 | 11.8 | 7.9 | 3.1 | 973.8 | .000*** | 128.5 | .000*** |
| **Entire underprivileged cohort** | 15152 | 7331 | 12017 | 126993 | 11.9 | 5.8 | 9.5 | 6.1 | 2.4 | 3923.0 | .000*** | 447.2 | .000*** |
| **Overall** | 16473 | 8196 | 13473 | 148652 | 11.1 | 5.5 | 9.1 | 5.6 | 2 | 3854.5 | .000*** | 542.0 | .000*** |

**Table S19.** Changes of incidence rates of identifying depression in each cohort after primary psychological healthcare policy, full names for abbreviations can be found at the supplemental results 1, * < .05, ** <.01, *** <.001, *** p < .0001

|  | **Overall** | **Entire underprivileged cohort** | **Typically developing cohort** | **dfUCA** | **Orphan** | **CEDC** | **“left-behind”** | **“single-parents”** |
| --- | --- | --- | --- | --- | --- | --- | --- | --- |
| RRR, % | 50.5 | 51.3 | 34.4 | 59.8 | 37.6 | 54.3 | 50.9 | 53.0 |
| P values of X^2^ test | - | < .0001 | - | <.0001 | < .0001 | <.0001 | <.0001 | <.0001 |

**Table S20.** Relative risk reduction (RRR) of primary psychological healthcare policy to incidence of depression for each cohort in the T2 v.s. T1 (baseline), the unit of each cell in this table is the percentage (%). These Chi-Square (X^2^) tests examined between-group differences (i.e., typical developing cohort and each underprivileged cohort) on incidence rate reduction, and “-” indicated “not applicable”.

|  | **Overall** | **Entire underprivileged cohort** | **Typically developing cohort** | **dfUCA** | **Orphan** | **CEDC** | **“left-behind”** | **“single-parents”** |
| --- | --- | --- | --- | --- | --- | --- | --- | --- |
| RRR, % | 18.0 | 20.2 | -9.8 | 30.4 | 27.2 | 8.6 | 21.4 | 20.8 |
| P values of X^2^ test | - | < .0001 | - | < .0001 | < .0001 | = .007 | < .0001 | < .0001 |

**Table S21.** Relative risk reduction (RRR) of primary psychological healthcare policy to incidence of depression for each cohort in the T3 v.s. T1 (baseline), the unit of each cell in this table is the percentage (%). These Chi-Square (X^2^) tests examined between-group differences (i.e., typically developing cohort and each underprivileged cohort) on incidence rate reduction, and “-” indicated “not applicable”.

|  | **Mild Symptom** | | | | **Moderate Symptom** | | | | **Severe Symptom** | | | |
| --- | --- | --- | --- | --- | --- | --- | --- | --- | --- | --- | --- | --- |
|  | **Boy, *n* of identifying depression** | **Incidence rates, % (95% CI)** | **Girl, *n* of identifying depression** | **Incidence rates, % (95% CI)** | **Boy, *n* of identifying depression** | **Incidence rates, % (95% CI)** | **Girl, *n* of identifying depression** | **Incidence rates, % (95% CI)** | **Boy, *n* of identifying depression** | **Incidence rates, % (95% CI)** | **Girl, *n* of identifying depression** | **Incidence rates, % (95% CI)** |
| **6-8** | 1 | 2.4 (-0.2 - 0.7) | — | — | — | — | — | — | 1 | 2.4 (-0.2 - 0.7) | — | — |
| **9-11** | 68 | 1.0 (0.8 - 1.3) | 104 | 1.7 (1.4 - 2.0 ) | 25 | 0.4 (0.2 – 0.5) | 41 | 0.7 (0.5 – 0.9) | 12 | 0.2 (0.1 – 0.3) | 10 | 0.2 (0.1 – 0.3) |
| **12-14** | 116 | 3.3 (2.7 - 3.8) | 236 | 7.2 (6.3 - 8.1) | 30 | 0.8 (0.5 - 1.1) | 55 | 1.7 (1.2 - 2.1) | 13 | 0.4 (0.2 - 0.6) | 3 | 0.1 (0.0 - 0.2) |
| **15-17** | 57 | 5.8 (4.4 - 7.3) | 65 | 7.1 (5.4 - 8.7) | 9 | 0.9 (0.3 - 1.5) | 11 | 1.2 (0.5 - 1.9) | 5 | 0.5 (0.1 - 1.0) | 2 | 0.2 (-0.1-0.5) |
| **18** | 1 | 5.0 (-4.6 - 14.6) | — | — | — | — | — | — | — | — | — | — |

**Table S22.** Sex-specific Incidence rates of identifying depression symptom (at different level) in typically developing cohort at the T2, full names for abbreviations can be found at the supplemental results 1, “-” indicates no applicable data are to be shown here.

|  | **Mild Symptom** | | | | **Moderate Symptom** | | | | **Severe Symptom** | | | |
| --- | --- | --- | --- | --- | --- | --- | --- | --- | --- | --- | --- | --- |
|  | **Boy, *n* of identifying depression** | **Incidence rates, % (95% CI)** | **Girl, *n* of identifying depression** | **Incidence rates, % (95% CI)** | **Boy, *n* of identifying depression** | **Incidence rates, % (95% CI)** | **Girl, *n* of identifying depression** | **Incidence rates, % (95% CI)** | **Boy, *n* of identifying depression** | **Incidence rates, % (95% CI)** | **Girl, *n* of identifying depression** | **Incidence rates, % (95% CI)** |
| **6-8** | — | — | — | — | — | — | — | — | — | — | — | — |
| **9-11** | 3 | 1.4 (-0.2 - 3.0) | 2 | 1.0 (-0.4 - 2.4) | 1 | 0.5 (-0.5 - 1.4) | 6 | 3.0 (0.6 - 5.4) | — | — | — | — |
| **12-14** | 4 | 1.8 (0.1 - 3.6) | 22 | 9.8 (5.9 - 13.7) | 2 | 0.9 (-0.3 - 2.2) | 3 | 1.3 (-0.2 - 2.8) | 3 | 1.4 (-0.2-2.9) | 1 | 0.4 (-0.4 - 1.3) |
| **15-17** | 10 | 6.6 (2.7 - 10.6) | 20 | 13.3 (7.9 - 18.8) | 3 | 2.0 (-0.2 - 4.2) | 3 | 2.0 (-0.2 - 4.2) | 2 | 1.3 (-0.5 - 3.1) | 1 | 0.7 (-0.6 - 2.0) |
| **18** | — | — | — | — | — | — | — | — | — | — | — | — |

**Table S23.** Sex-specific Incidence rates of identifying depression symptom (at different level) in the de facto unattended children/adolescents cohort at the T2, full names for abbreviations can be found at the supplemental results 1, “-” indicates no applicable data are to be shown here.

|  | **Mild Symptom** | | | | **Moderate Symptom** | | | | **Severe Symptom** | | | |
| --- | --- | --- | --- | --- | --- | --- | --- | --- | --- | --- | --- | --- |
|  | **Boy, *n* of identifying depression** | **Incidence rates, % (95% CI)** | **Girl, *n* of identifying depression** | **Incidence rates, % (95% CI)** | **Boy, *n* of identifying depression** | **Incidence rates, % (95% CI)** | **Girl, *n* of identifying depression** | **Incidence rates, % (95% CI)** | **Boy, *n* of identifying depression** | **Incidence rates, % (95% CI)** | **Girl, *n* of identifying depression** | **Incidence rates, % (95% CI)** |
| **6-8** | — | — | — | — | — | — | — | — | — | — | — | — |
| **9-11** | 2 | 3.3 (-1.2 - 7.9) | 1 | 2.0 (-1.9 - 5.9) | — | — | 1 | 2.0 (-1.9 - 5.9) | — | — | — | — |
| **12-14** | 4 | 4.5 (0.2 - 8.9) | 8 | 12.3 (4.3 - 20.3) | 1 | 1.1 (-1.1 - 3.4) | 2 | 3.1 (-1.1 - 7.3) | 1 | 1.1 (-1.1 - 3.4) | 2 | 3.1 (-1.1 - 7.3) |
| **15-17** | 6 | 13.6 (3.5 - 23.8) | 5 | 14.3 (2.7 - 25.9) | 1 | 2.3 (-2.1 - 6.7) | 1 | 2.9 (-2.7 - 8.4) | 2 | 4.5 (-1.6 - 10.7) | — | — |
| **18** | — | — | — | — | — | — | — | — | — | — | — | — |

**Table S24.** Sex-specific Incidence rates of identifying depression symptom (at different level) in the orphan cohort at the T2, full names for abbreviations can be found at the supplemental results 1, “-” indicates no applicable data are to be shown here.

|  | **Mild Symptom** | | | | **Moderate Symptom** | | | | **Severe Symptom** | | | |
| --- | --- | --- | --- | --- | --- | --- | --- | --- | --- | --- | --- | --- |
|  | **Boy, *n* of identifying depression** | **Incidence rates, % (95% CI)** | **Girl, *n* of identifying depression** | **Incidence rates, % (95% CI)** | **Boy, *n* of identifying depression** | **Incidence rates, % (95% CI)** | **Girl, *n* of identifying depression** | **Incidence rates, % (95% CI)** | **Boy, *n* of identifying depression** | **Incidence rates, % (95% CI)** | **Girl, *n* of identifying depression** | **Incidence rates, % (95% CI)** |
| **6-8** | — | — | — | — | — | — | — | — | — | — | — | — |
| **9-11** | 37 | 1.3 (0.9 - 1.8) | 35 | 1.5 (1.0 - 2.0) | 10 | 0.4 (0.1- 0.6) | 17 | 0.7 (0.4 - 1.1) | 6 | 0.2 (0.0 - 0.4) | 2 | 0.1 (0.0 - 0.2) |
| **12-14** | 66 | 3.8 (2.9 - 4.7) | 132 | 9.1 (7.6 - 10.6) | 15 | 0.9 (0.4 - 1.3) | 23 | 1.6 (0.9 - 2.2) | 4 | 0.2 (0.0 - 0.5) | 5 | 0.3 (0.0 - 0.6) |
| **15-17** | 34 | 6.6 (4.4 - 8.7) | 44 | 9.0 (6.5 - 11.6) | 5 | 1.0 (0.1 - 1.8) | 5 | 1.0 (0.1 - 1.9) | 6 | 1.2 (0.2 - 2.1) | 1 | 0.2 (-0.2 - 0.6) |
| **18** | — | — | — | — | — | — | — | — | — | — | — | — |

**Table S25.** Sex-specific Incidence rates of identifying depression symptom (at different level) in the children/adolescents in especially difficult circumstance (CEDC) at the T2, full names for abbreviations can be found at the supplemental results 1, “-” indicates no applicable data are to be shown here.

|  | **Mild Symptom** | | | | **Moderate Symptom** | | | | **Severe Symptom** | | | |
| --- | --- | --- | --- | --- | --- | --- | --- | --- | --- | --- | --- | --- |
|  | **Boy, *n* of identifying depression** | **Incidence rates, % (95% CI)** | **Girl, *n* of identifying depression** | **Incidence rates, % (95% CI)** | **Boy, *n* of identifying depression** | **Incidence rates, % (95% CI)** | **Girl, *n* of identifying depression** | **Incidence rates, % (95% CI)** | **Boy, *n* of identifying depression** | **Incidence rates, % (95% CI)** | **Girl, *n* of identifying depression** | **Incidence rates, % (95% CI)** |
| **6-8** | — | — | 3 | 2.3 (-0.3 - 4.8) | — | — | 1 | 0.8 (-0.7 - 2.2) | 1 | 1.2 (-1.1 - 3.5) | — | — |
| **9-11** | 192 | 1.0 (0.9 - 1.1) | 415 | 2.2 (2.0 - 2.4) | 70 | 0.4 (0.3 - 0.4) | 155 | 0.8 (0.7 - 1.0) | 24 | 0.1 (0.1 - 0.2) | 30 | 0.2 (0.1 - 0.2) |
| **12-14** | 658 | 3.6 (3.3 - 3.8) | 1418 | 7.9 (7.5 - 8.3) | 160 | 0.9 (0.7 - 1.0) | 276 | 1.5 (1.4 - 1.7) | 59 | 0.3 (0.2 - 0.4) | 63 | 0.4 (0.3 - 0.4) |
| **15-17** | 522 | 5.8 (5.3 - 6.3) | 773 | 9.2 (8.5 - 9.8) | 88 | 1.0 (0.8 - 1.2) | 93 | 1.1 (0.9 - 1.3) | 38 | 0.4 (0.3 - 0.6) | 21 | 0.2 (0.1 - 0.4) |
| **18** | 9 | 4.3 (1.5 - 7.0) | 12 | 10.3 (4.8 - 15.9) | 1 | 0.5 (-0.5 - 1.4) | — | — | — | — | — | — |

**Table S26.** Sex-specific Incidence rates of identifying depression symptom (at different level) in the left-behind children/adolescents at the T2, full names for abbreviations can be found at the supplemental results 1, “-” indicates no applicable data are to be shown here.

|  | **Mild Symptom** | | | | **Moderate Symptom** | | | | **Severe Symptom** | | | |
| --- | --- | --- | --- | --- | --- | --- | --- | --- | --- | --- | --- | --- |
|  | **Boy, *n* of identifying depression** | **Incidence rates, % (95% CI)** | **Girl, *n* of identifying depression** | **Incidence rates, % (95% CI)** | **Boy, *n* of identifying depression** | **Incidence rates, % (95% CI)** | **Girl, *n* of identifying depression** | **Incidence rates, % (95% CI)** | **Boy, *n* of identifying depression** | **Incidence rates, % (95% CI)** | **Girl, *n* of identifying depression** | **Incidence rates, % (95% CI)** |
| **6-8** | — | — | — | — | 1 | 3.3 (-3.1 - 9.8) | — | — | 1 | 6.3 (-5.6 -18.1) | — | — |
| **9-11** | 58 | 1.2 (0.9 - 1.6) | 140 | 3.0 (2.5 - 3.5) | 26 | 0.6 (0.3 - 0.8) | 61 | 1.3 (1.0 - 1.7) | 9 | 0.2 (0.1 - 0.3) | 12 | 0.3 (0.1 - 0.4) |
| **12-14** | 208 | 4.4 (3.8 - 5.0) | 489 | 9.4 (8.6 - 10.2) | 55 | 1.2 (0.9 - 1.5) | 100 | 1.9 (1.6 - 2.3) | 23 | 0.5 (0.3 - 0.7) | 29 | 0.6 (0.4 - 0.8) |
| **15-17** | 149 | 6.5 (5.5 - 7.5) | 231 | 10.5 (9.2 -11.8) | 26 | 1.1 (0.7 - 1.6) | 36 | 1.6 (1.1 - 2.2) | 13 | 0.6 (0.3 - 0.9) | 8 | 0.4 (0.1 - 0.6) |
| **18** | 1 | 2.1 (-2.0 - 6.3) | 3 | 10.3 (-0.7 -21.4) | — | — | — | — | — | — | — | — |

**Table S27.** Sex-specific Incidence rates of identifying depression symptom (at different level) in the single-parent children/adolescents at the T2, full names for abbreviations can be found at the supplemental results 1, “-” indicates no applicable data are to be shown here.

| **Cohorts** | **Boys, *n*** | **Girls, *n*** | **Mild Symptom** | | | | **Moderate Symptom** | | | | **Severe Symptom** | | | |
| --- | --- | --- | --- | --- | --- | --- | --- | --- | --- | --- | --- | --- | --- | --- |
|  |  |  | **Boy, *n* of identifying depression** | **Incidence rates, % (95% CI)** | **Girl, *n* of identifying depression** | **Incidence rates, % (95% CI)** | **Boy, *n* of identifying depression** | **Incidence rates, % (95% CI)** | **Girl, *n* of identifying depression** | **Incidence rates, % (95% CI)** | **Boy, *n* of identifying depression** | **Incidence rates, % (95% CI)** | **Girl, *n* of identifying depression** | **Incidence rates, % (95% CI)** |
| Typically developing cohort | 11187 | 10472 | 453 | 4.0 (3.7 – 4.4) | 816 | 7.8 (7.3 – 8.3) | 42 | 0.4 (0.3 – 0.5) | 124 | 1.2 (1.0 – 1.4) | 6 | 0.1 (0.0 – 0.1) | 15 | 0.1 (0.1 – 0.2) |
| dfUCA | 589 | 576 | 40 | 6.8 (4.8 – 8.8) | 79 | 13.7 (10.9 – 16.5) | 8 | 1.4 (0.4 – 2.3) | 22 | 3.8 (2.3 – 5.4) | — | — | — | — |
| Orphan | 192 | 150 | 16 | 8.3 (4.4 – 12.2) | 21 | 14.0 (8.4 – 19.6) | — | — | 5 | 3.3 (0.5 – 6.2) | — | — | 1 | 0.7 (-0.6 – 2.0) |
| CEDC | 5043 | 4308 | 318 | 6.3 (5.6 – 7.0) | 451 | 10.5 (9.6 – 11.4) | 39 | 0.8 (0.5 – 1.0) | 78 | 1.8 (1.4 – 2.2) | 5 | 0.1 (0.0 – 0.2) | 9 | 0.2 (0.1 – 0.3) |
| “left-Behind” | 47034 | 45257 | 2469 | 5.2 (5.0 – 5.5) | 4442 | 9.8 (9.5 – 10.1) | 263 | 0.6 (0.5 – 0.6) | 832 | 1.8 (1.7 – 2.0) | 19 | 0.0 (0.0 – 0.1) | 76 | 0.2 (0.1 – 0.2) |
| “single-Parents” | 11786 | 12058 | 813 | 6.9 (6.4 – 7.4) | 1536 | 12.7 (12.1 – 13.3) | 98 | 0.8 (0.7 – 1.0) | 333 | 2.8 (2.5 – 3.1) | 11 | 0.1 (0.0 – 0.1) | 33 | 0.3 (0.2 – 0.4) |
| Entire underprivileged cohort | 64644 | 62349 | 3656 | 5.7 (5.5 – 5.8) | 6529 | 10.5 (10.2 – 10.7) | 408 | 0.6 (0.6 – 0.7) | 1270 | 2.0 (1.9 – 2.1) | 35 | 0.1 (0.0 – 0.1) | 119 | 0.2 (0.2 – 0.2) |
| **Overall** | 75831 | 72821 | 4109 | 5.4 (5.3 – 5.6) | 7345 | 10.1 (9.9 – 10.3) | 450 | 0.6 (0.5 – 0.6) | 1394 | 1.9 (1.8 – 2.0) | 41 | 0.1 (0.0 – 0.1) | 134 | 0.2 (0.2 – 0.2) |

**Table S28.** Sex-specific Incidence rates of identifying depression symptom (at different level) in each cohort at the T3, full names for abbreviations can be found at the supplemental results 1, these categories for defining depression levels are formulated by criterion of Zung’s SDS.

|  | **Mild Symptom** | | | | **Moderate Symptom** | | | | **Severe Symptom** | | | |
| --- | --- | --- | --- | --- | --- | --- | --- | --- | --- | --- | --- | --- |
|  | **Boy, *n* of identifying depression** | **Incidence rates, % (95% CI)** | **Girl, *n* of identifying depression** | **Incidence rates, % (95% CI)** | **Boy, *n* of identifying depression** | **Incidence rates, % (95% CI)** | **Girl, *n* of identifying depression** | **Incidence rates, % (95% CI)** | **Boy, *n* of identifying depression** | **Incidence rates, % (95% CI)** | **Girl, *n* of identifying depression** | **Incidence rates, % (95% CI)** |
| **6-8** | — | — | 3 | 3.6 (-0.4 - 7.5) | — | — | — | — | — | — | — | — |
| **9-11** | 227 | 3.4 (3.0 – 3.9) | 368 | 6.0 (5.4 – 6.5) | 20 | 0.3 (0.2 – 0.4) | 52 | 0.8 (0.6 – 1.1) | 3 | 0.0 (0.0 – 0.1) | 4 | 0.1 (0.0 – 0.1) |
| **12-14** | 153 | 4.3 (3.6 - 5.0) | 346 | 10.5 (9.5 – 11.6) | 16 | 0.4 (0.2 – 0.7) | 64 | 1.9 (1.5 – 2.4) | 2 | 0.1 (0.0 - 0.1) | 8 | 0.2 (0.1 – 0.4) |
| **15-17** | 73 | 7.5 (5.8 – 9.1) | 98 | 10.7 (8.7 – 12.7) | 6 | 0.6 (0.1 - 1.1) | 8 | 0.9 (0.3 – 1.5) | 1 | 0.1 (-0.1 - 0.3) | 3 | 0.3 (0.0 – 0.7) |
| **18** | — | — | 1 | 16.7 (-13.2 – 46.5) | — | — | — | — | — | — | — | — |

**Table S29.** Sex-specific Incidence rates of identifying depression symptom (at different level) in typically developing cohort at the T3, full names for abbreviations can be found at the supplemental results 1, “-” indicates no applicable data are to be shown here.

|  | **Mild Symptom** | | | | **Moderate Symptom** | | | | **Severe Symptom** | | | |
| --- | --- | --- | --- | --- | --- | --- | --- | --- | --- | --- | --- | --- |
|  | **Boy, *n* of identifying depression** | **Incidence rates, % (95% CI)** | **Girl, *n* of identifying depression** | **Incidence rates, % (95% CI)** | **Boy, *n* of identifying depression** | **Incidence rates, % (95% CI)** | **Girl, *n* of identifying depression** | **Incidence rates, % (95% CI)** | **Boy, *n* of identifying depression** | **Incidence rates, % (95% CI)** | **Girl, *n* of identifying depression** | **Incidence rates, % (95% CI)** |
| **6-8** | — | — | — | — | — | — | — | — | — | — | — | — |
| **9-11** | 14 | 6.6 (3.3 – 9.9) | 25 | 12.7 (8.0 – 17.3) | — | — | 4 | 2.0 (0.1 – 4.0) | — | — | — | — |
| **12-14** | 15 | 6.9 (3.5 – 10.2) | 31 | 13.8 (9.3 – 18.3) | 2 | 0.9 (-0.3 – 2.2) | 12 | 5.3 (2.4 – 8.3) | — | — | — | — |
| **15-17** | 11 | 7.3 (3.1 – 11.4) | 23 | 15.3 (9.6 – 21.1) | 6 | 4.0 (0.9 – 7.1) | 6 | 4.0 (0.9 – 7.1) | — | — | — | — |
| **18** | — | — | — | — | — | — | — | — | — | — | — | — |

**Table S30.** Sex-specific Incidence rates of identifying depression symptom (at different level) in the de facto unattended children/adolescents cohort at the T3, full names for abbreviations can be found at the supplemental results 1, “-” indicates no applicable data are to be shown here.

|  | **Mild Symptom** | | | | **Moderate Symptom** | | | | **Severe Symptom** | | | |
| --- | --- | --- | --- | --- | --- | --- | --- | --- | --- | --- | --- | --- |
|  | **Boy, *n* of identifying depression** | **Incidence rates, % (95% CI)** | **Girl, *n* of identifying depression** | **Incidence rates, % (95% CI)** | **Boy, *n* of identifying depression** | **Incidence rates, % (95% CI)** | **Girl, *n* of identifying depression** | **Incidence rates, % (95% CI)** | **Boy, *n* of identifying depression** | **Incidence rates, % (95% CI)** | **Girl, *n* of identifying depression** | **Incidence rates, % (95% CI)** |
| **6-8** | — | — | — | — | — | — | — | — | — | — | — | — |
| **9-11** | 4 | 6.7 (0.4 – 13.0) | 3 | 6.0 (-0.6 – 12.6) | — | — | 1 | 2.0 (-1.9 – 5.9) | — | — | — | — |
| **12-14** | 7 | 8.0 (2.3 – 13.6) | 11 | 16.9 (7.8 – 26.0) | — | — | 2 | 3.1 (-1.1 – 7.3) | — | — | — | — |
| **15-17** | 5 | 11.4 (2.0 – 20.7) | 7 | 20.0 (6.7 – 33.3) | — | — | 2 | 5.7 (-2.0 – 13.4) | — | — | 1 | 2.9 (-2.7 – 8.4) |
| **18** | — | — | — | — | — | — | — | — | — | — | — | — |

**Table S31.** Sex-specific Incidence rates of identifying depression symptom (at different level) in the orphan cohort at the T3, full names for abbreviations can be found at the supplemental results 1, “-” indicates no applicable data are to be shown here.

|  | **Mild Symptom** | | | | **Moderate Symptom** | | | | **Severe Symptom** | | | |
| --- | --- | --- | --- | --- | --- | --- | --- | --- | --- | --- | --- | --- |
|  | **Boy, *n* of identifying depression** | **Incidence rates, % (95% CI)** | **Girl, *n* of identifying depression** | **Incidence rates, % (95% CI)** | **Boy, *n* of identifying depression** | **Incidence rates, % (95% CI)** | **Girl, *n* of identifying depression** | **Incidence rates, % (95% CI)** | **Boy, *n* of identifying depression** | **Incidence rates, % (95% CI)** | **Girl, *n* of identifying depression** | **Incidence rates, % (95% CI)** |
| **6-8** | — | — | 2 | 8.0 (-2.6 – 18.6) | — | — | — | — | — | — | — | — |
| **9-11** | 142 | 5.1 (4.3 – 5.9) | 174 | 7.4 (6.4 – 8.5) | 18 | 0.6 (0.4 – 0.9) | 33 | 1.4 (0.9- 1.9) | 3 | 0.1 (0.0 – 0.2) | 2 | 0.1 (0.0 – 0.2) |
| **12-14** | 114 | 6.6 (5.4 – 7.7) | 190 | 13.1 (11.4 – 14.9) | 15 | 0.9 (0.4 – 1.3) | 30 | 2.1 (1.3 – 2.8) | 2 | 0.1 (0.0 – 0.3) | 4 | 0.3 (0.0 – 0.5) |
| **15-17** | 61 | 11.8 (9.0 – 14.6) | 84 | 17.2 (13.9 – 20.6) | 6 | 1.2 (0.2 – 2.1) | 15 | 3.1 (1.5 – 4.6) | — | — | 3 | 0.6 (-0.1 – 1.3) |
| **18** | 1 | 10.0 (-8.6 – 28.6) | 1 | 16.7 (-13.2 – 46.5) | — | — | — | — | — | — | — | — |

**Table S32.** Sex-specific Incidence rates of identifying depression symptom (at different level) in the children/adolescents in especially difficult circumstance (CEDC) cohort at the T3, full names for abbreviations can be found at the supplemental results 1, “-” indicates no applicable data are to be shown here.

|  | **Mild Symptom** | | | | **Moderate Symptom** | | | | **Severe Symptom** | | | |
| --- | --- | --- | --- | --- | --- | --- | --- | --- | --- | --- | --- | --- |
|  | **Boy, *n* of identifying depression** | **Incidence rates, % (95% CI)** | **Girl, *n* of identifying depression** | **Incidence rates, % (95% CI)** | **Boy, *n* of identifying depression** | **Incidence rates, % (95% CI)** | **Girl, *n* of identifying depression** | **Incidence rates, % (95% CI)** | **Boy, *n* of identifying depression** | **Incidence rates, % (95% CI)** | **Girl, *n* of identifying depression** | **Incidence rates, % (95% CI)** |
| **6-8** | — | — | 8 | 6.0 (2.0 – 10.1) | — | — | — | — | — | — | — | — |
| **9-11** | 819 | 4.3 (4.0 – 4.5) | 1334 | 7.2 (6.8 – 7.5) | 68 | 0.4 (0.3 – 0.4) | 227 | 1.2 (1.1 – 1.4) | 8 | 0.0 (0.0 - 0.1) | 22 | 0.1 (0.1 – 0.2) |
| **12-14** | 1040 | 5.6 (5.3 – 6.0) | 2065 | 11.5 (11.0 – 12.0) | 130 | 0.7 (0.6 – 0.8) | 451 | 2.5 (2.3 – 2.7) | 6 | 0.0 (0.0 - 0.1) | 43 | 0.2 (0.2 – 0.3) |
| **15-17** | 603 | 6.7 (6.2 – 7.2) | 1012 | 12.0 (11.3 – 12.7) | 61 | 0.7 (0.5 – 0.8) | 152 | 1.8 (1.5 – 2.1) | 5 | 0.1 (0.0 – 0.1) | 11 | 0.1 (0.1 – 0.2) |
| **18** | 7 | 3.3 (0.9 – 5.7) | 23 | 19.8 (12.6 – 27.1) | 4 | 1.9 (0.1 – 3.7) | 2 | 1.7 (-0.6 – 4.1) | — | — | — | — |

**Table S33.** Sex-specific Incidence rates of identifying depression symptom (at different level) in the “left-behind” children/adolescent cohort at the T3, full names for abbreviations can be found at the supplemental results 1, “-” indicates no applicable data are to be shown here.

|  | **Mild Symptom** | | | | **Moderate Symptom** | | | | **Severe Symptom** | | | |
| --- | --- | --- | --- | --- | --- | --- | --- | --- | --- | --- | --- | --- |
|  | **Boy, *n* of identifying depression** | **Incidence rates, % (95% CI)** | **Girl, *n* of identifying depression** | **Incidence rates, % (95% CI)** | **Boy, *n* of identifying depression** | **Incidence rates, % (95% CI)** | **Girl, *n* of identifying depression** | **Incidence rates, % (95% CI)** | **Boy, *n* of identifying depression** | **Incidence rates, % (95% CI)** | **Girl, *n* of identifying depression** | **Incidence rates, % (95% CI)** |
| **6-8** | — | — | 1 | 3.3 (-3.1 – 9.8) | — | — | — | — | — | — | — | — |
| **9-11** | 263 | 5.6 (5.0 – 6.3) | 451 | 9.8 (8.9 – 10.6) | 27 | 0.6 (0.4 – 0.8) | 92 | 2.0 (1.6 – 2.4) | 4 | 0.1 (0.0 – 0.2) | 11 | 0.2 (0.1 – 0.4) |
| **12-14** | 373 | 7.8 (7.1 – 8.6) | 759 | 14.6 (13.7 – 15.6) | 48 | 1.0 (0.7 – 1.3) | 188 | 3.6 (3.1 – 4.1) | 5 | 0.1 (0.0 – 0.2) | 19 | 0.4 (0.2 – 0.5) |
| **15-17** | 176 | 7.7 (6.6 – 8.8) | 319 | 14.5 (13.0 – 16.0) | 23 | 1.0 (0.6 – 1.4) | 52 | 2.4 (1.7 – 3.0) | 2 | 0.1 (0.0 – 0.2) | 3 | 0.1 (0.0 – 0.3) |
| **18** | 1 | 2.1 (2.0 – 6.3) | 6 | 20.7 (5.9 – 35.4) | — | — | 1 | 3.4 (-3.2 – 10.1) | — | — | — | — |

**Table S34.** Sex-specific Incidence rates of identifying depression symptom (at different level) in the “single-parenting” children/adolescent cohort at the T3, full names for abbreviations can be found at the supplemental results 1, “-” indicates no applicable data are to be shown here

| Sex | Age (years) | Total sample size of this cohort | Number of identifying depression at this follow-up (Oct 30, 2022, T1) | Incidence rates, % | Number of identifying depression at this follow-up (May 21, 2023, T2) | Incidence rates, % | Number of identifying depression at this follow-up (Oct 29, 2023, T3) | Incidence rates, % | McNemar(T1-T2), α | P value (two-side,T1-T2) | McNemar(T1-T3), α | P value (two-side,T1-T3) |
| --- | --- | --- | --- | --- | --- | --- | --- | --- | --- | --- | --- | --- |
| Total | 6-8 | 126 | 1 | 0.8 | 2 | 1.6 | 3 | 2.4 | 0.0 | 1.000 | 0.3 | 0.625 |
| Total | 9-11 | 12757 | 355 | 2.8 | 260 | 2.0 | 674 | 5.3 | 18.6 | <0.001^***^ | 122.3 | <0.001^***^ |
| Total | 12-14 | 6855 | 662 | 9.7 | 453 | 6.6 | 589 | 8.6 | 56.3 | <0.001^***^ | 6.1 | 0.013^*^ |
| Total | 15-17 | 1895 | 299 | 15.8 | 149 | 7.9 | 189 | 10.0 | 64.9 | <0.001^***^ | 38.6 | <0.001^***^ |
| Total | 18 | 26 | 4 | 15.4 | 1 | 3.8 | 1 | 3.8 | 0.8 | 0.375 | 0.8 | 0.375 |
| Boy | 6-8 | 42 | 0 | 0.0 | 2 | 4.8 | 0 | 0.0 | 0.5 | 0.500 | - | - |
| Boy | 9-11 | 6581 | 171 | 2.6 | 105 | 1.6 | 250 | 3.8 | 19.2 | <0.001^***^ | 17.2 | <0.001^***^ |
| Boy | 12-14 | 3567 | 221 | 6.2 | 159 | 4.5 | 171 | 4.8 | 13.5 | <0.001^***^ | 8.2 | 0.004^**^ |
| Boy | 15-17 | 977 | 123 | 12.6 | 71 | 7.3 | 80 | 8.2 | 17.6 | <0.001^***^ | 12.9 | <0.001^***^ |
| Boy | 18 | 20 | 3 | 15.0 | 1 | 5.0 | 0 | 0.0 | 0.3 | 0.625 | 1.3 | 0.250 |
| Girl | 6-8 | 84 | 1 | 1.2 | 0 | 0.0 | 3 | 3.6 | 0.0 | 1.000 | 0.3 | 0.625 |
| Girl | 9-11 | 6176 | 184 | 3.0 | 155 | 2.5 | 424 | 6.9 | 3.1 | 0.080 | 120.5 | <0.001^***^ |
| Girl | 12-14 | 3288 | 441 | 13.4 | 294 | 8.9 | 418 | 12.7 | 43.2 | <0.001^***^ | 0.9 | 0.350 |
| Girl | 15-17 | 918 | 176 | 19.2 | 78 | 8.5 | 109 | 11.9 | 48.5 | <0.001^***^ | 25.5 | <0.001^***^ |
| Girl | 18 | 6 | 1 | 16.7 | 0 | 0.0 | 1 | 16.7 | 0.0 | 1.000 | 0.0 | 1.000 |

**Table S35.** Changes of incidence rates of identifying depression in the typically developing cohort after primary psychological healthcare, “-” indicates no applicable data are to be shown here. * < .025, ** <.01, *** <.001 (Bonferroni correction: α/2= 0.05/2 = 0.025).

| Sex | Age (years) | Total sample size of this cohort | Number of identifying depression at this follow-up (Oct 30, 2022, T1) | Incidence rates, % | Number of identifying depression at this follow-up (May 21, 2023, T2) | Incidence rates, % | Number of identifying depression at this follow-up (Oct 29, 2023, T3) | Incidence rates, % | McNemar(T1-T2), α | P value (two-side,T1-T2) | McNemar(T1-T3), α | P value (two-side,T1-T3) |
| --- | --- | --- | --- | --- | --- | --- | --- | --- | --- | --- | --- | --- |
| Total | 6-8 | 6 | 0 | 0.0 | 0 | 0.0 | 0 | 0.0 | - | - | - | - |
| Total | 9-11 | 409 | 39 | 9.5 | 12 | 2.9 | 43 | 10.5 | 16.5 | <0.001^***^ | 0.1 | 0.720 |
| Total | 12-14 | 443 | 89 | 20.1 | 35 | 7.9 | 60 | 13.5 | 29.3 | <0.001^***^ | 7.9 | 0.005^**^ |
| Total | 15-17 | 301 | 84 | 27.9 | 39 | 13.0 | 46 | 15.3 | 27.3 | <0.001^***^ | 19.6 | <0.001^***^ |
| Total | 18 | 6 | 2 | 33.3 | 0 | 0.0 | 0 | 0.0 | 0.5 | 0.500 | 0.5 | 0.500 |
| Boy | 6-8 | 3 | 0 | 0.0 | 0 | 0.0 | 0 | 0.0 | - | - | - | - |
| Boy | 9-11 | 212 | 21 | 9.9 | 4 | 1.9 | 14 | 6.6 | 11.1 | 0.000^***^ | 1.1 | 0.296 |
| Boy | 12-14 | 218 | 27 | 12.4 | 9 | 4.1 | 17 | 7.8 | 9.0 | 0.003^***^ | 2.3 | 0.134 |
| Boy | 15-17 | 151 | 31 | 20.5 | 15 | 9.9 | 17 | 11.3 | 8.7 | 0.003^***^ | 6.5 | 0.011^*^ |
| Boy | 18 | 5 | 2 | 40.0 | 0 | 0.0 | 0 | 0.0 | 1.3 | 0.250 | 1.3 | 0.250 |
| Girl | 6-8 | 3 | 0 | 0.0 | 0 | 0.0 | 0 | 0.0 | - | - | - | - |
| Girl | 9-11 | 197 | 18 | 9.1 | 8 | 4.1 | 29 | 14.7 | 4.5 | 0.031 | 2.7 | 0.100 |
| Girl | 12-14 | 225 | 62 | 27.6 | 26 | 11.6 | 43 | 19.1 | 19.1 | <0.001^***^ | 5.1 | 0.023^*^ |
| Girl | 15-17 | 150 | 53 | 35.3 | 24 | 16.0 | 29 | 19.3 | 17.4 | <0.001^***^ | 12.0 | <0.001^***^ |
| Girl | 18 | 1 | 0 | 0.0 | 0 | 0.0 | 0 | 0.0 | - | - | - | - |
| **Table S36.** Changes of incidence rates of identifying depression in the de facto unattended children/adolescents cohort after primary psychological healthcare, “-” indicates no applicable data are to be shown here. * < .025, ** <.01, *** <.001. (Bonferroni correction: α/2= 0.05/2 = 0.025). | | | | | | | | | | | | |
| Sex | Age (years) | Total sample size of this cohort | Number of identifying depression at this follow-up (Oct 30, 2022, T1) | Incidence rates, % | Number of identifying depression at this follow-up (May 21, 2023, T2) | Incidence rates, % | Number of identifying depression at this follow-up (Oct 29, 2023, T3) | Incidence rates, % | McNemar(T1-T2), α | P value (two-side,T1-T2) | McNemar(T1-T3), α | P value (two-side,T1-T3) |
| Total | 6-8 | 0 | 0 | - | 0 | - | 0 | - | - | - | - | - |
| Total | 9-11 | 110 | 7 | 6.4 | 4 | 3.6 | 8 | 7.3 | 0.4 | 0.549 | 0.0 | 1.000 |
| Total | 12-14 | 153 | 29 | 19.0 | 18 | 11.8 | 20 | 13.1 | 3.2 | 0.072 | 2.8 | 0.093 |
| Total | 15-17 | 79 | 23 | 29.1 | 15 | 19.0 | 15 | 19.0 | 2.2 | 0.134 | 2.5 | 0.115 |
| Total | 18 | 0 | 0 | - | 0 | - | 0 | - | - | - | - | - |
| Boy | 6-8 | 0 | 0 | - | 0 | - | 0 | - | - | - | - | - |
| Boy | 9-11 | 60 | 3 | 5.0 | 2 | 3.3 | 4 | 6.7 | 0.0 | 1.000 | 0.0 | 1.000 |
| Boy | 12-14 | 88 | 13 | 14.8 | 6 | 6.8 | 7 | 8.0 | 2.8 | 0.092 | 3.1 | 0.070 |
| Boy | 15-17 | 44 | 10 | 22.7 | 9 | 20.5 | 5 | 11.4 | 0.0 | 1.000 | 1.8 | 0.180 |
| Boy | 18 | 0 | 0 | - | 0 | - | 0 | - | - | - | - | - |
| Girl | 6-8 | 0 | 0 | - | 0 | - | 0 | - | - | - | - | - |
| Girl | 9-11 | 50 | 4 | 8.0 | 2 | 4.0 | 4 | 8.0 | 0.2 | 0.688 | 0.0 | 1.000 |
| Girl | 12-14 | 65 | 16 | 24.6 | 12 | 18.5 | 13 | 20.0 | 0.5 | 0.481 | 0.3 | 0.607 |
| Girl | 15-17 | 35 | 13 | 37.1 | 6 | 17.1 | 10 | 28.6 | 2.8 | 0.092 | 0.4 | 0.549 |
| Girl | 18 | 0 | 0 | - | 0 | - | 0 | - | - | - | - | - |

**Table S37.** Changes of incidence rates of identifying depression in the orphan cohort after primary psychological healthcare, “-” indicates no applicable data are to be shown here. * < .025, ** <.01, *** <.001. (Bonferroni correction: α/2= 0.05/2 = 0.025).

| Sex | Age (years) | Total sample size of this cohort | Number of identifying depression at this follow-up (Oct 30, 2022, T1) | Incidence rates, % | Number of identifying depression at this follow-up (May 21, 2023, T2) | Incidence rates, % | Number of identifying depression at this follow-up (Oct 29, 2023, T3) | Incidence rates, % | McNemar(T1-T2), α | P value (two-side,T1-T2) | McNemar(T1-T3), α | P value (two-side,T1-T3) |
| --- | --- | --- | --- | --- | --- | --- | --- | --- | --- | --- | --- | --- |
| Total | 6-8 | 36 | 1 | 2.8 | 0 | 0.0 | 2 | 5.6 | 0.0 | 1.000 | 0.0 | 1.000 |
| Total | 9-11 | 5116 | 250 | 4.9 | 107 | 2.1 | 372 | 7.3 | 64.4 | <0.001^***^ | 28.9 | <0.001^***^ |
| Total | 12-14 | 3178 | 430 | 13.5 | 245 | 7.7 | 355 | 11.2 | 71.9 | <0.001^***^ | 10.6 | 0.001^**^ |
| Total | 15-17 | 1005 | 296 | 29.5 | 95 | 9.5 | 169 | 16.8 | 152.1 | <0.001^***^ | 58.2 | <0.001^***^ |
| Total | 18 | 16 | 5 | 31.3 | 0 | 0.0 | 2 | 12.5 | 3.2 | 0.063 | 0.8 | 0.375 |
| Boy | 6-8 | 11 | 0 | 0.0 | 0 | 0.0 | 0 | 0.0 | - | - | - | - |
| Boy | 9-11 | 2772 | 127 | 4.6 | 53 | 1.9 | 163 | 5.9 | 34.2 | <0.001^***^ | 5.0 | 0.026 |
| Boy | 12-14 | 1732 | 175 | 10.1 | 85 | 4.9 | 131 | 7.6 | 40.4 | <0.001^***^ | 8.7 | 0.003^**^ |
| Boy | 15-17 | 518 | 137 | 26.4 | 45 | 8.7 | 67 | 12.9 | 69.0 | <0.001^***^ | 37.2 | <0.001^***^ |
| Boy | 18 | 10 | 4 | 40.0 | 0 | 0.0 | 1 | 10.0 | 2.3 | 0.125 | 1.3 | 0.250 |
| Girl | 6-8 | 25 | 1 | 4.0 | 0 | 0.0 | 2 | 8.0 | 0.0 | 1.000 | 0.0 | 1.000 |
| Girl | 9-11 | 2344 | 123 | 5.2 | 54 | 2.3 | 209 | 8.9 | 29.5 | <0.001^***^ | 27.8 | <0.001^***^ |
| Girl | 12-14 | 1446 | 255 | 17.6 | 160 | 11.1 | 224 | 15.5 | 32.1 | <0.001^***^ | 3.0 | 0.086 |
| Girl | 15-17 | 487 | 159 | 32.6 | 50 | 10.3 | 102 | 20.9 | 81.6 | <0.001^***^ | 21.6 | <0.001^***^ |
| Girl | 18 | 6 | 1 | 16.7 | 0 | 0.0 | 1 | 16.7 | 0.0 | 1.000 | 0.0 | 1.000 |

**Table S38.** Changes of incidence rates of identifying depression in the children/adolescents in especially difficult circumstance (CEDC) cohort after primary psychological healthcare, “-” indicates no applicable data are to be shown here. * < .025, ** <.01, *** <.001. (Bonferroni correction: α/2= 0.05/2 = 0.025).

| Sex | | Age (years) | | Total sample size of this cohort | | Number of identifying depression at this follow-up (Oct 30, 2022, T1) | | Incidence rates, % | | Number of identifying depression at this follow-up (May 21, 2023, T2) | | Incidence rates, % | | Number of identifying depression at this follow-up (Oct 29, 2023, T3) | | Incidence rates, % | | McNemar(T1-T2), α | | P value (two-side,T1-T2) | | McNemar(T1-T3), α | | P value (two-side,T1-T3) | |
| --- | --- | --- | --- | --- | --- | --- | --- | --- | --- | --- | --- | --- | --- | --- | --- | --- | --- | --- | --- | --- | --- | --- | --- | --- | --- |
| Total | | 6-8 | | 216 | | 6 | | 2.8 | | 5 | | 2.3 | | 8 | | 3.7 | | 0.0 | | 1.000 | | 0.1 | | 0.774 | |
| Total | | 9-11 | | 37847 | | 1772 | | 4.7 | | 886 | | 2.3 | | 2478 | | 6.5 | | 371.2 | | <0.001^***^ | | 145.8 | | <0.001^***^ | |
| Total | | 12-14 | | 36413 | | 4926 | | 13.5 | | 2634 | | 7.0 | | 3735 | | 10.3 | | 1008.6 | | <0.001^***^ | | 253.8 | | <0.001^***^ | |
| Total | | 15-17 | | 17488 | | 3571 | | 20.4 | | 1535 | | 8.8 | | 1844 | | 10.5 | | 1169.2 | | <0.001^***^ | | 885.8 | | <0.001^***^ | |
| Total | | 18 | | 327 | | 72 | | 22.0 | | 22 | | 6.7 | | 36 | | 11.0 | | 30.0 | | <0.001^***^ | | 17.5 | | <0.001^***^ | |
| Boy | | 6-8 | | 84 | | 0 | | 0.0 | | 1 | | 1.2 | | 0 | | 0.0 | | 0.0 | | 1.000 | | - | | - | |
| Boy | | 9-11 | | 19248 | | 693 | | 3.6 | | 286 | | 1.5 | | 895 | | 4.6 | | 200.3 | | <0.001^***^ | | 30.4 | | <0.001^***^ | |
| Boy | | 12-14 | | 18451 | | 1666 | | 9.0 | | 877 | | 4.8 | | 1176 | | 6.4 | | 321.6 | | <0.001^***^ | | 118.1 | | <0.001^***^ | |
| Boy | | 15-17 | | 9040 | | 1456 | | 16.1 | | 648 | | 7.2 | | 669 | | 7.4 | | 428.5 | | <0.001^***^ | | 407.2 | | <0.001^***^ | |
| Boy | | 18 | | 211 | | 41 | | 19.4 | | 10 | | 4.7 | | 11 | | 5.2 | | 20.0 | | <0.001^***^ | | 20.0 | | <0.001^***^ | |
| Girl | | 6-8 | | 132 | | 6 | | 4.5 | | 4 | | 3.0 | | 8 | | 6.1 | | 0.1 | | 0.754 | | 0.1 | | 0.774 | |
| Girl | | 9-11 | | 18599 | | 1079 | | 5.8 | | 600 | | 3.2 | | 1583 | | 8.5 | | 177.5 | | <0.001^***^ | | 121.6 | | <0.001^***^ | |
| Girl | | 12-14 | | 17962 | | 3260 | | 18.1 | | 1757 | | 9.8 | | 2559 | | 14.2 | | 689.3 | | <0.001^***^ | | 137.8 | | <0.001^***^ | |
| Girl | | 15-17 | | 8448 | | 2115 | | 25.0 | | 887 | | 10.5 | | 1175 | | 13.9 | | 744.6 | | <0.001^***^ | | 477.6 | | <0.001^***^ | |
| Girl | | 18 | | 116 | | 31 | | 26.7 | | 12 | | 10.3 | | 25 | | 21.6 | | 9.3 | | 0.002^**^ | | 0.9 | | 0.345 | |
| **Table S39.** Changes of incidence rates of identifying depression in the “left-behind” children/adolescent cohort after primary psychological healthcare, “-” indicates no applicable data are to be shown here. * < .025, ** <.01, *** <.001. (Bonferroni correction: α/2= 0.05/2 = 0.025). | | | | | | | | | | | | | | | | | | | | | | | | | |
| Sex | | Age (years) | | Total sample size of this cohort | | Number of identifying depression at this follow-up (Oct 30, 2022, T1) | | Incidence rates, % | | Number of identifying depression at this follow-up (May 21, 2023, T2) | | Incidence rates, % | | Number of identifying depression at this follow-up (Oct 29, 2023, T3) | | Incidence rates, % | | McNemar(T1-T2), α | | P value (two-side,T1-T2) | | McNemar(T1-T3), α | | P value (two-side,T1-T3) | |
| Total | | 6-8 | | 46 | | 1 | | 2.2 | | 2 | | 4.3 | | 1 | | 2.2 | | 0.0 | | 1.000 | | 0.0 | | 1.000 | |
| Total | | 9-11 | | 9290 | | 606 | | 6.5 | | 306 | | 3.3 | | 848 | | 9.1 | | 125.6 | | <0.001^***^ | | 50.8 | | <0.001^***^ | |
| Total | | 12-14 | | 9943 | | 1801 | | 18.1 | | 904 | | 9.1 | | 1392 | | 14.0 | | 448.3 | | <0.001^***^ | | 86.3 | | <0.001^***^ | |
| Total | | 15-17 | | 4489 | | 1119 | | 24.9 | | 463 | | 10.3 | | 575 | | 12.8 | | 404.7 | | <0.001^***^ | | 295.4 | | <0.001^***^ | |
| Total | | 18 | | 76 | | 23 | | 30.3 | | 4 | | 5.3 | | 8 | | 10.5 | | 13.0 | | 0.000^***^ | | 10.3 | | 0.001^***^ | |
| Boy | | 6-8 | | 16 | | 0 | | 0.0 | | 1 | | 6.3 | | 0 | | 0.0 | | 0.0 | | 1.000 | | - | | - | |
| Boy | | 9-11 | | 4680 | | 244 | | 5.2 | | 93 | | 2.0 | | 294 | | 6.3 | | 80.6 | | <0.001^***^ | | 5.2 | | 0.022^*^ | |
| Boy | | 12-14 | | 4756 | | 581 | | 12.2 | | 286 | | 6.0 | | 426 | | 9.0 | | 132.8 | | <0.001^***^ | | 34.7 | | <0.001^***^ | |
| Boy | | 15-17 | | 2287 | | 458 | | 20.0 | | 188 | | 8.2 | | 201 | | 8.8 | | 160.1 | | <0.001^***^ | | 150.7 | | <0.001^***^ | |
| Boy | | 18 | | 47 | | 12 | | 25.5 | | 1 | | 2.1 | | 1 | | 2.1 | | 7.7 | | 0.003^**^ | | 7.7 | | 0.003^**^ | |
| Girl | | 6-8 | | 30 | | 1 | | 3.3 | | 1 | | 3.3 | | 1 | | 3.3 | | 0.0 | | 1.000 | | 0.0 | | 1.000 | |
| Girl | | 9-11 | | 4610 | | 362 | | 7.9 | | 213 | | 4.6 | | 554 | | 12.0 | | 50.6 | | <0.001^***^ | | 53.3 | | <0.001^***^ | |
| Girl | | 12-14 | | 5187 | | 1220 | | 23.5 | | 618 | | 11.9 | | 966 | | 18.6 | | 316.8 | | <0.001^***^ | | 51.4 | | <0.001^***^ | |
| Girl | | 15-17 | | 2202 | | 661 | | 30.0 | | 275 | | 12.5 | | 374 | | 17.0 | | 243.8 | | <0.001^***^ | | 145.3 | | <0.001^***^ | |
| Girl | | 18 | | 29 | | 11 | | 37.9 | | 3 | | 10.3 | | 7 | | 24.1 | | 4.1 | | 0.039 | | 1.5 | | 0.219 | |
| **Table S40.** Changes of incidence rates of identifying depression in the “single-parenting” children/adolescent cohort after primary psychological healthcare, “-” indicates no applicable data are to be shown here. * < .025, ** <.01, *** <.001. (Bonferroni correction: α/2= 0.05/2 = 0.025). | | | | | | | | | | | | | | | | | | | | | | | | | |

|  | **Typically developing cohort**  **(n = 30,524)** | **De facto unattended cohort**  **(n =1,769)** | **Orphan cohort**  **(n = 518)** | **Children in especially difficult circumstance cohort**  **(n = 13,550)** | **“Left-behind” cohort**  **(n = 134,153)** | **“Single-parent” cohort**  **(n = 34,927)** |
| --- | --- | --- | --- | --- | --- | --- |
| **Age, years** | 11 (10 - 13) | 13 (11 - 15) | 13 (11 - 15) | 11 (10 - 14) | 13 (11 - 15) | 13 (11 - 14) |
| **Sex** |  |  |  |  |  |  |
| Female | 14, 848 (48.64%) | 880 (49.75%) | 223 (43.05%) | 6, 259 (46.19%) | 65, 863 (49.10%) | 17, 758 (50.84%) |
| Male | 15, 676 (51.36%) | 889 (50.25%) | 295 (56.95%) | 7, 291 (53.81%) | 68, 290 (50.90%) | 17, 169 (49.16%) |
| **Living Areas** |  |  |  |  |  |  |
| Urban | 19, 107 (62.60%) | 547 (30.92%) | 129 (24.90%) | 4, 048(29.87%) | 50, 952 (37.98%) | 14, 138(40.48%) |
| Rural | 11, 417 (37.40%) | 1, 222 (69.08%) | 389 (75.10%) | 9, 502 (70.13%) | 83, 201 (62.02%) | 20, 789(59.52%) |
| **No. of offspring** |  |  |  |  |  |  |
| One | 4, 466(14.63%) | 435 (24.59%) | 222 (42.86%) | 2, 426 (17.90%) | 24, 555 (18.30%) | 12, 070 (34.56%) |
| More than one | 26, 281 (85.37%) | 1, 334 (75.41%) | 296 (57.14%) | 11, 124 (82.10%) | 109, 598(81.70%) | 22, 857(65.44%) |
| **Family economic status** |  |  |  |  |  |  |
| Poor | 1 (0%) | - | - | 13, 458 (100%) | 9, 070 (6.76%) | 3, 359(9.62%) |
| Middle | 23, 092 (75.65%) | - | - | 0 (0%) | 42, 329 (31.55%) | 9, 878 (28.28%) |
| Upper-middle | 7, 277 (23.84%) | - | - | 0 (0%) | 9, 819(7.32%) | 2, 242 (6.42%) |
| Rich | 154 (0.50%) | - | - | 0 (0%) | 137 (0.1%) | 45 (0.13%) |
| Unwilling to answer | 0 (0%) | - | - | 2 (0%) | 72, 798(54.26%) | 19, 403(55.55%) |
| **Family subjective ES** |  |  |  |  |  |  |
| Always satisfied | 2, 955 (9.68%) | - | - | 1, 482(10.94%) | 73, 005 (54.42%) | 19, 686(56.36%) |
| Sometime satisfied | 226 (0.74%) | - | - | 20(0.15%) | 4, 557 (3.4 %) | 902 (2.58%) |
| Unsatisfied | 61 (0.20%) | - | - | 3(0.02%) | 788 (0.59%) | 225 (0.64%) |
| Unwilling to answer | 27, 282 (89.38%) | - | - | 12, 045 (88.89%) | 55, 803 (41.60%) | 14, 114(40.41%) |

**Table S41.** Sociodemographic characteristics of the population that was successfully followed at the May 21, 2023. Data are n/N or median (IQR). Data were extracted from CPHG group across 596 sites, which covered the almost all the areas of Nanchong city (Sichuan, China). Questions relating to family status were omitted for all the children/adolescents living outside family (i.e., de facto unattended and orphan cohorts). Family economic statuses are categorized by national criterion that made by National Bureau of Statistics in the China, with family income per year (FIY) < 60, 000 CNY for poor, and with 60, 000 ≤ FIY < 150, 000 for middle economic level, and with 150, 000 ≤ FIY < 30, 0000 for upper-middle economic level, and with FIY ≥ 300, 000 for rich income level. ES = economic status. Both parametric and non-parametric estimates have been conducted, and showed no statistically significant differences between this follow-up sample and the baseline sample for these sociodemographic characteristics

|  | **Mild Symptom** | | | | **Moderate Symptom** | | | | **Severe Symptom** | | | |
| --- | --- | --- | --- | --- | --- | --- | --- | --- | --- | --- | --- | --- |
|  | **Boy, *n* of identifying depression** | **Incidence rates, % (95% CI)** | **Girl, *n* of identifying depression** | **Incidence rates, % (95% CI)** | **Boy, *n* of identifying depression** | **Incidence rates, % (95% CI)** | **Girl, *n* of identifying depression** | **Incidence rates, % (95% CI)** | **Boy, *n* of identifying depression** | **Incidence rates, % (95% CI)** | **Girl, *n* of identifying depression** | **Incidence rates, % (95% CI)** |
| **6-8** | 1 | 2.1 (-2.0 - 6.1) | — | — | — | — | — | — | 1 | 2.1 (-2.0 - 6.1) | — | — |
| **9-11** | 97 | 1.1 (0.9 - 1.3) | 142 | 1.7 (1.4 - 2.0) | 29 | 0.3 (0.2 - 0.5) | 53 | 0.6 (0.5 - 0.8) | 13 | 0.2 (0.1 - 0.2) | 12 | 0.1 (0.1 - 0.2) |
| **12-14** | 162 | 3.2 (2.7 - 3.7) | 326 | 7.1 (63 - 7.8) | 38 | 0.8 (0.5 - 1.0) | 73 | 1.6 (1.2 - 1.9) | 20 | 0.4 (0.2 - 0.6) | 8 | 0.2 (0.1 - 0.3) |
| **15-17** | 102 | 5.7 (4.6 - 6.8) | 143 | 7.7 (6.5 - 8.9) | 15 | 0.8 (0.4 - 1.3) | 27 | 1.4 ( 0.9 - 2.0) | 7 | 0.4 (0.1 - 0.7) | 3 | 0.2 (-0.0 - 0.3) |
| **18** | 9 | 8.5 (3.2 - 13.8) | 5 | 6.3 (1.0 - 11.7) | 4 | 3.8 (0.1 - 7.4) | 1 | 1.3 (-1.2 - 3.7) | 1 | 0.9 (-0.9 - 2.8) | — | — |

**Table S42.** Sex-specific Incidence rates of identifying depression symptom (at different level) in typically developing cohort at the half-year follow-up investigation, full names for abbreviations can be found at the supplemental results 1, “-” indicates no applicable data are to be shown here

|  | **Mild Symptom** | | | | **Moderate Symptom** | | | | **Severe Symptom** | | | |
| --- | --- | --- | --- | --- | --- | --- | --- | --- | --- | --- | --- | --- |
|  | **Boy, *n* of identifying depression** | **Incidence rates, % (95% CI)** | **Girl, *n* of identifying depression** | **Incidence rates, % (95% CI)** | **Boy, *n* of identifying depression** | **Incidence rates, % (95% CI)** | **Girl, *n* of identifying depression** | **Incidence rates, % (95% CI)** | **Boy, *n* of identifying depression** | **Incidence rates, % (95% CI)** | **Girl, *n* of identifying depression** | **Incidence rates, % (95% CI)** |
| **6-8** | — | — | — | — | — | — | — | — | — | — | — | — |
| **9-11** | 3 | 1.1 (-0.1 - 0.3) | 5 | 1.9 (0.3 - 3.6) | 2 | 0.7 (-0.3 - 1.7) | 7 | 2.7 (0.7 - 4.7) | 2 | 0.7 (-0.3 - 1.7) | — | — |
| **12-14** | 8 | 2.7 ( 0.8 - 4.5) | 28 | 8.8 (5.7 - 11.8) | 4 | 1.3 (0.0 - 2.6) | 4 | 1.2 (0.0 - 2.5) | 5 | 1.7 (0.2 - 3.1) | 1 | 0.3 (-0.3 - 0.9) |
| **15-17** | 19 | 6.8 (3.8 - 9.7) | 32 | 11.3 (7.6 - 15.0) | 8 | 2.8 (0.9 - 4.8) | 4 | 1.4 (0.0 - 2.8) | 3 | 1.1 (-0.1 - 2.3) | 2 | 0.7 (-0.3 - 1.7) |
| **18** | — | — | 3 | 20.07 (-0.2 - 40.2) | — | — | — | — | — | — | — | — |

**Table S43.** Sex-specific Incidence rates of identifying depression symptom (at different level) in the de facto unattended children/adolescents cohort at the half-year follow-up investigation, full names for abbreviations can be found at the supplemental results 1, “-” indicates no applicable data are to be shown here

|  | **Mild Symptom** | | | | **Moderate Symptom** | | | | **Severe Symptom** | | | |
| --- | --- | --- | --- | --- | --- | --- | --- | --- | --- | --- | --- | --- |
|  | **Boy, *n* of identifying depression** | **Incidence rates, % (95% CI)** | **Girl, *n* of identifying depression** | **Incidence rates, % (95% CI)** | **Boy, *n* of identifying depression** | **Incidence rates, % (95% CI)** | **Girl, *n* of identifying depression** | **Incidence rates, % (95% CI)** | **Boy, *n* of identifying depression** | **Incidence rates, % (95% CI)** | **Girl, *n* of identifying depression** | **Incidence rates, % (95% CI)** |
| **6-8** | — | — | — | — | — | — | — | — | — | — | — | — |
| **9-11** | 3 | 3.7 (0.4 - 7.7) | 1 | 1.6 (-1.5 - 4.7) | — | — | 1 | 1.6 (-1.5 - 4.7) | — | — | — | — |
| **12-14** | 4 | 3.5 (0.1 - 6.8) | 13 | 13.5 (6.7 - 20.4) | 1 | 0.9 (-0.8 - 2.6) | 3 | 3.1 (-0.4 - 6.6) | 1 | 0.9 (-0.8 - 2.6) | 3 | 3.1 (-0.4 - 6.6) |
| **15-17** | 11 | 12.8 (5.7 - 19.8) | 7 | 11.5 (3.5 - 19.5) | 2 | 2.3 (-0.9 - 5.5) | 4 | 6.6 (0.3 - 12.8) | 2 | 2.3 (-0.9 - 5.5) | — | — |
| **18** | — | — | 2 | 66.7 (13.3 - 120.0) | — | — | — | — | — | — | — | — |

**Table S44.** Sex-specific Incidence rates of identifying depression symptom (at different level) in the orphan cohort at the half-year follow-up investigation, full names for abbreviations can be found at the supplemental results 1, “-” indicates no applicable data are to be shown here

|  | **Mild Symptom** | | | | **Moderate Symptom** | | | | **Severe Symptom** | | | |
| --- | --- | --- | --- | --- | --- | --- | --- | --- | --- | --- | --- | --- |
|  | **Boy, *n* of identifying depression** | **Incidence rates, % (95% CI)** | **Girl, *n* of identifying depression** | **Incidence rates, % (95% CI)** | **Boy, *n* of identifying depression** | **Incidence rates, % (95% CI)** | **Girl, *n* of identifying depression** | **Incidence rates, % (95% CI)** | **Boy, *n* of identifying depression** | **Incidence rates, % (95% CI)** | **Girl, *n* of identifying depression** | **Incidence rates, % (95% CI)** |
| **6-8** | — | — | — | — | — | — | — | — | — | — | — | — |
| **9-11** | 48 | 1.3(0.9 - 1.7) | 46 | 1.5(1.1 - 1.9) | 14 | 0.4(0.2 - 0.6) | 21 | 0.7(0.4 - 1.0) | 6 | 0.2(0.0 - 0.3) | 2 | 0.1(-0.0 - 0.2) |
| **12-14** | 104 | 4.2(3.4 - 5.0) | 177 | 8.7(7.5 - 9.9) | 16 | 0.6(0.3 - 1.0) | 34 | 1.7(1.1 - 2.2) | 8 | 0.3(0.1 - 0.5) | 8 | 0.4(0.1 - 0.7) |
| **15-17** | 64 | 6.3(4.8 - 7.8) | 120 | 11.3(9.4 - 13.2) | 13 | 1.3(0.6 - 2.0) | 13 | 1.2(0.6 - 1.9) | 7 | 0.7(0.2 - 1.2) | 3 | 0.3(-0.0 - 0.6) |
| **18** | 6 | 7.9(1.8 - 14.0) | 5 | 8.6(1.4 - 15.8) | — | — | 1 | 1.7(-1.6 - 5.1) | 1 | 1.3(-1.2 - 3.9) | — | — |

**Table S45.** Sex-specific Incidence rates of identifying depression symptom (at different level) in the children/adolescents in especially difficult circumstance (CEDC) cohort at the half-year follow-up investigation, full names for abbreviations can be found at the supplemental results 1, “-” indicates no applicable data are to be shown here

|  | **Mild Symptom** | | | | **Moderate Symptom** | | | | **Severe Symptom** | | | |
| --- | --- | --- | --- | --- | --- | --- | --- | --- | --- | --- | --- | --- |
|  | **Boy, *n* of identifying depression** | **Incidence rates, % (95% CI)** | **Girl, *n* of identifying depression** | **Incidence rates, % (95% CI)** | **Boy, *n* of identifying depression** | **Incidence rates, % (95% CI)** | **Girl, *n* of identifying depression** | **Incidence rates, % (95% CI)** | **Boy, *n* of identifying depression** | **Incidence rates, % (95% CI)** | **Girl, *n* of identifying depression** | **Incidence rates, % (95% CI)** |
| **6-8** | — | — | 3 | 1.7 (-0.2 - 3.7) | 1 | 0.1 (-0.9 - 2.9) | 1 | 0.6 (-0.5 - 0.01.7) | 1 | 1.0 (-0.9 - 2.9) | — | — |
| **9-11** | 250 | 1.0 (0.9 - 1.1) | 540 | 2.2 (2.1 - 2.5) | 100 | 0.4 (0.3 - 0.5) | 211 | 0.9 (0.8 - 1.0) | 27 | 0.1 (0.1 - 0.1) | 39 | 0.2 (0.1 - 0.2) |
| **12-14** | 978 | 3.8 (3.5 - 4.0) | 2014 | 8.0 (7.7 - 8.3) | 221 | 0.8 (0.7 - 1.0) | 395 | 1.6 (1.4 - 1.7) | 92 | 0.4 (0.3 - 0.4) | 99 | 0.4 (0.3 - 0.5) |
| **15-17** | 905 | 5.6 (5.3 - 6.0) | 1430 | 9.1 (8.6 - 9.5) | 169 | 1.1 (0.9 - 1.2) | 185 | 1.2 (1.0 - 1.3) | 54 | 0.3 (0.2 - 0.4) | 39 | 0.2 (0.2 - 0.3) |
| **18** | 73 | 6.0 (4.7 - 7.3) | 78 | 8.1 (6.4 - 9.9) | 9 | 0.7 (0.3 - 1.2) | 10 | 1.0 (0.4 - 1.7) | 5 | 0.4 (0.1 - 0.8) | 1 | 0.1 (-0.1 - 0.3) |

**Table S46.** Sex-specific Incidence rates of identifying depression symptom (at different level) in the “left-behind” children/adolescent cohort at the half-year follow-up investigation, full names for abbreviations can be found at the supplemental results 1, “-” indicates no applicable data are to be shown here

|  | **Mild Symptom** | | | | **Moderate Symptom** | | | | **Severe Symptom** | | | |
| --- | --- | --- | --- | --- | --- | --- | --- | --- | --- | --- | --- | --- |
|  | **Boy, *n* of identifying depression** | **Incidence rates, % (95% CI)** | **Girl, *n* of identifying depression** | **Incidence rates, % (95% CI)** | **Boy, *n* of identifying depression** | **Incidence rates, % (95% CI)** | **Girl, *n* of identifying depression** | **Incidence rates, % (95% CI)** | **Boy, *n* of identifying depression** | **Incidence rates, % (95% CI)** | **Girl, *n* of identifying depression** | **Incidence rates, % (95% CI)** |
| **6-8** | — | — | — | — | — | — | 1 | 2.8 (-2.6 - 8.1) | 1 | 5.3 (-4.8 - 15.3) | — | — |
| **9-11** | 76 | 1.2 (1.0 - 1.5) | 190 | 3.1 (2.7 - 3.6) | 35 | 0.6 (0.4 - 0.8) | 83 | 1.4 (1.1 - 1.7) | 10 | 0.2 (0.1 - 0.3) | 18 | 0.3 (0.2 -0. 4) |
| **12-14** | 315 | 4.6 (4.1 - 5.1) | 698 | 9.6 (8.9 - 10.2) | 81 | 1.5 (0.9 - 1.4) | 136 | 1.9 (1.6 - 2.2) | 39 | 0.6 (0.4 - 0.8) | 41 | 0.6 (0.4 - 0.7) |
| **15-17** | 248 | 6.3 (5.5 - 7.0) | 435 | 10.6 (9.6 - 11.5) | 53 | 1.3 (1.0 - 1.7) | 60 | 1.5 (1.1 - 1.8) | 16 | 0.4 (0.2 - 0.6) | 15 | 0.4 (0.2 - 0.5) |
| **18** | 27 | 9.5 (6.1 - 13.0) | 18 | 7.3 (4.0 - 10.5) | 1 | 0.4 (-0.3 - 1.0) | 3 | 1.2 (-0.2 - 2.6) | 1 | 0.4 (-0.3 - 1.0) | 1 | 0.4 (-0.4 - 1.2) |

**Table S47.** Sex-specific Incidence rates of identifying depression symptom (at different level) in the “single-parenting” children/adolescent cohort at the half-year follow-up investigation, full names for abbreviations can be found at the supplemental results 1, “-” indicates no applicable data are to be shown here

1. **Results of noninferiority tests**

We further used noninferiority tests to examine whether such primary psychological healthcare could be workable to these underprivileged children/adolescents as noninferior as to typically developing cohorts. Given a little evidence to indicate the optimal noninferior boundary, we iterated all the candidates of noninferior boundaries ranged from 5% to 10%, with step for 1%. Results showed that the effects of primary psychological healthcare on underprivileged cohorts were significantly noninferior to typically developing ones (all p _corrected_ < 0.0001). These findings have been illustrated in the Figure S1.


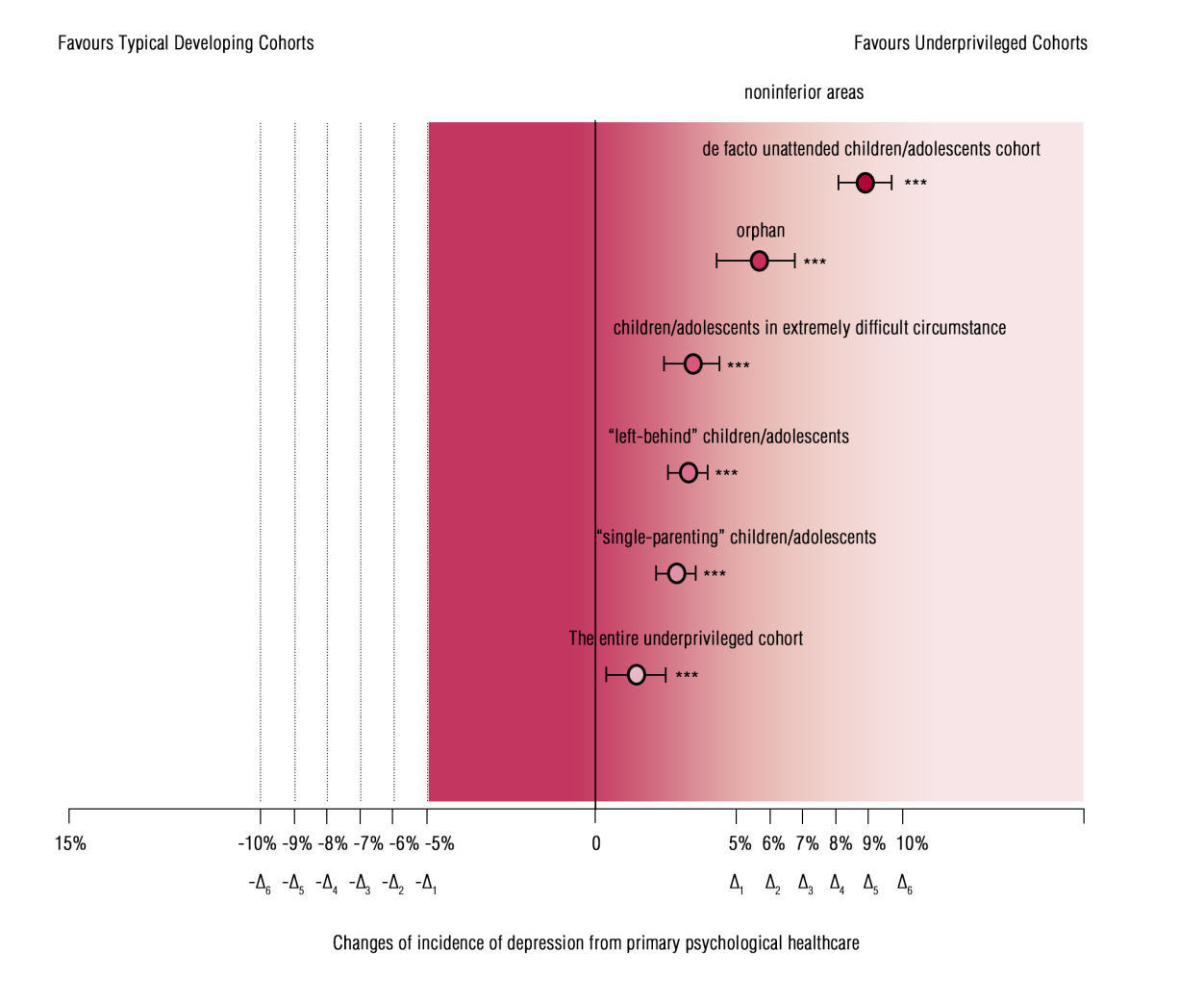


**Figure S1.** Results of non-inferiority tests for all the cohorts. *** p < .0001 at Holm-Bonferrini correction estimated by the z tests.

1. **Results of Cochran-Armitage tests**

We estimated the linear trends of incidence rates of identifying depression across three follow-ups for the CFPS observational control group (that is outside from this primary psychological healthcare system) and the samples in the current study by using Cochran-Armitage tests. We found the statistical uptrend for incidence of depression across T1 (14.4%, 2016), T2 (15.9%, 2018) and T3 (24.5%, 2020) in this control group (z = 5.0, p for trend < .0001). On the other hand, we found the statistically significant downtrend on incidence of depression across T1 (11.1%, Oct, 2022), T2 (5.5%; May, 2023) and T3 (9.1%; Oct, 2023) in the present sample that was involved into this primary psychological healthcare system (z = -19.7, p _for trend_ < .0001).

**8. The difference on depression trajectory between children/adolescents with and without the two-round early psychological interventions.**

Linear mixed models (LMM) were conducted to analyze the difference on depression trajectory between those with and without the two-round early psychological interventions. The dependent variable refers to the CES-D score. LMM for outcome includes fixed effects for children/adolescents with or without the two-round early psychological interventions, different follow-up periods, as well as the interaction effects between these variables, adjusted for age and sex. A random effect for clustering of children/adolescents within schools was accounted to capture variability between groups. Results showed that the interaction effects between those with or without the two-round early psychological interventions and different follow-up periods were significant. These findings have been illustrated in the Table S48 and Figure S2.

| Predictors | Estimates (95% CI) | p |
| --- | --- | --- |
| (intercept) | -1.63 (-1.68, -1.57) | <0.001 |
| Follow-up |  |  |
| Baseline | Reference |  |
| Half-year follow-up | 1.42 (1.36, 1.47) | <0.001 |
| One-year follow-up | 6.10 (5.95, 6.26) | <0.001 |
| Group |  |  |
| Outside^a^ | Reference |  |
| Included^b^ | 22.8 (22.78, 22.82) | <0.001 |
| Sex |  |  |
| Girl | Reference |  |
| Boy | -1.35 (-1.36, -1.33) | <0.001 |
| Age | 0.58 (0.36, 0.80) | <0.001 |
| Half-year follow-up : Included | -7.94 (-8.16, -7.72) | <0.001 |
| One-year follow-up : Included | -13.34 (-13.39, -13.28) | <0.001 |
| Random effects |  |  |
| σ^2^ | 59.51 |  |
| τ_00_ school | 1.74 |  |
| N_school_ | 563 |  |
| Observations | 438,882 |  |

**Table S48.** The practical effects of implementing two-round early psychological interventions on CES-D scores. Linear mixed models were used for the analysis. a. Outside refers to children/adolescents without the two-round early psychological interventions. b. Included refers to children/adolescents without the two-round early psychological interventions.


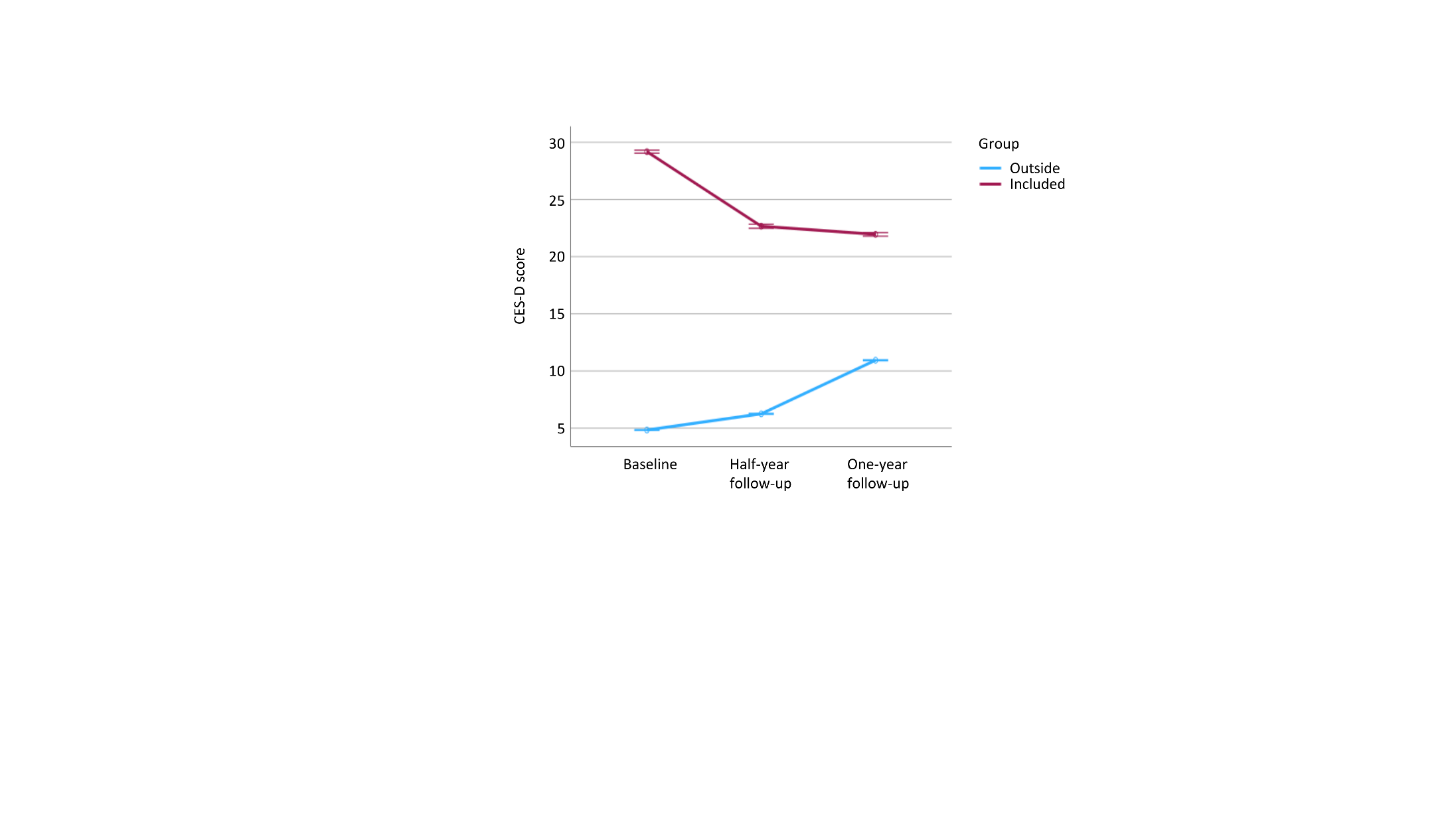


**Figure S2.** The trajectory of CES-D scores of children/adolescents with or without two-round early psychological interventions. Outside refers to individuals without two-round early psychological interventions; Included refers to individuals with two-round early psychological interventions.

**9. Public budget statement**

As legal statement required, we limited to disclose the budget statement that permitted by accounting firms, to favor the understandings of the estimates for costs. Please see as follow:

| **Source of Funding** | |
| --- | --- |
| 1.Project Funding Application | 2 million |
| 2.Own Funds |  |
| 3.Other Funding Support | 1.5 million (Nanchong Civil Affairs) |
| **The purpose of this funding application** | |
| 1. Operating Expenses | 2 million（100%） |
| 1. Labor Costs | 700 thousand（35%） |
| A. Supervision Fee | 10,0000 RMB (5%) is primarily allocated for evaluation, training, post-assessment, inviting experts for project process consultation, and expert supervision expenses. |
| B. Social Worker Fee | 60,000 RMB (3%) is allocated to provide subsidies for social workers participating in the project. |
| C. Volunteer Allowance | 60,000 RMB（3%）is allocated to provide subsidies for the volunteers participating in the project. |
| D. Indirect Costs | 480,000 RMB（24%）is allocated for performance-based expenses of personnel involved in the project. |
| 1. Project Activity Expenditures | 1.3 million（65%） |
| A. Adolescent Psychological General Assessment | 360,000 RMB (18%) is primarily allocated for the bi-annual psychological assessment of children and adolescents in Nanchong City. |
| B. Training for Full-time and Part-time Psychology Teachers | 240,000 RMB (12%) is primarily allocated for the training and professional development of full-time and part-time mental health teachers in the entire city. |
| C. Post-assessment Services | 440,000 RMB (22%) is primarily allocated for psychological interviews, counseling referrals, and follow-up for disadvantaged children and post-assessment. |
| D. Popularization of Mental Health Knowledge | 140,000 RMB (7%) is primarily allocated for the writing of popular science scripts, expert fees, recording costs, and campus popular science activities. |
| E. Project Publicity | 120,000 RMB (6%) is primarily allocated for project promotion and publicity. |
| 1. Others |  |
| **The use of own and other project funds** | |
| The municipal matching fund is 1.5 million yuan. The assessment costs for minors in the entire city, excluding disadvantaged children (73.34%), as well as indirect expenses of 400,000 yuan (26.66%). | |

**10. STROBE statement**

STROBE Statement—Checklist of items that should be included in reports of ***cohort studies***

|  | Item No | Recommendation | Page |
| --- | --- | --- | --- |
| **Title and abstract** | 1 | (*a*) Indicate the study’s design with a commonly used term in the title or the abstract | 1 |
|  |  | (*b*) Provide in the abstract an informative and balanced summary of what was done and what was found | 3 |
| Introduction | | |  |
| Background/rationale | 2 | Explain the scientific background and rationale for the investigation being reported | 6 |
| Objectives | 3 | State specific objectives, including any prespecified hypotheses | 7 |
| Methods | | |  |
| Study design | 4 | Present key elements of study design early in the paper | 7 |
| Setting | 5 | Describe the setting, locations, and relevant dates, including periods of recruitment, exposure, follow-up, and data collection | 7-8 |
| Participants | 6 | (*a*) Give the eligibility criteria, and the sources and methods of selection of participants. Describe methods of follow-up | 7-8 |
|  |  | (*b*) For matched studies, give matching criteria and number of exposed and unexposed | N.A. |
| Variables | 7 | Clearly define all outcomes, exposures, predictors, potential confounders, and effect modifiers. Give diagnostic criteria, if applicable | 9 |
| Data sources/ measurement | 8* | For each variable of interest, give sources of data and details of methods of assessment (measurement). Describe comparability of assessment methods if there is more than one group | 8-9 |
| Bias | 9 | Describe any efforts to address potential sources of bias | 9-10 |
| Study size | 10 | Explain how the study size was arrived at | N.A. |
| Quantitative variables | 11 | Explain how quantitative variables were handled in the analyses. If applicable, describe which groupings were chosen and why | 9 |
| Statistical methods | 12 | (*a*) Describe all statistical methods, including those used to control for confounding | 9 |
|  |  | (*b*) Describe any methods used to examine subgroups and interactions | 9 |
|  |  | (*c*) Explain how missing data were addressed | N.A. |
|  |  | (*d*) If applicable, explain how loss to follow-up was addressed | N.A. |
|  |  | (*e*) Describe any sensitivity analyses | 9 |
| Results | | |  |
| Participants | 13* | (a) Report numbers of individuals at each stage of study—eg numbers potentially eligible, examined for eligibility, confirmed eligible, included in the study, completing follow-up, and analysed | 10 |
|  |  | (b) Give reasons for non-participation at each stage | 10 |
|  |  | (c) Consider use of a flow diagram | N.A. |
| Descriptive data | 14* | (a) Give characteristics of study participants (eg demographic, clinical, social) and information on exposures and potential confounders | 10 and table 1 |
|  |  | (b) Indicate number of participants with missing data for each variable of interest | N.A. |
|  |  | (c) Summarise follow-up time (eg, average and total amount) | 10 |
| Outcome data | 15* | Report numbers of outcome events or summary measures over time | 10-11 |
| Main results | 16 | (*a*) Give unadjusted estimates and, if applicable, confounder-adjusted estimates and their precision (eg, 95% confidence interval). Make clear which confounders were adjusted for and why they were included | 10-11 |
|  |  | (*b*) Report category boundaries when continuous variables were categorized | N.A. |
|  |  | (*c*) If relevant, consider translating estimates of relative risk into absolute risk for a meaningful time period | N.A. |
| Other analyses | 17 | Report other analyses done—eg analyses of subgroups and interactions, and sensitivity analyses | 10-11 |
| Discussion | | |  |
| Key results | 18 | Summarise key results with reference to study objectives | 11 |
| Limitations | 19 | Discuss limitations of the study, taking into account sources of potential bias or imprecision. Discuss both direction and magnitude of any potential bias | 12 |
| Interpretation | 20 | Give a cautious overall interpretation of results considering objectives, limitations, multiplicity of analyses, results from similar studies, and other relevant evidence | 11-12 |
| Generalisability | 21 | Discuss the generalisability (external validity) of the study results | 12 |
| Other information | | |  |
| Funding | 22 | Give the source of funding and the role of the funders for the present study and, if applicable, for the original study on which the present article is based | 16 |

*Give information separately for exposed and unexposed groups.

**Note:** An Explanation and Elaboration article discusses each checklist item and gives methodological background and published examples of transparent reporting. The STROBE checklist is best used in conjunction with this article (freely available on the Web sites of PLoS Medicine at http://www.plosmedicine.org/, Annals of Internal Medicine at http://www.annals.org/, and Epidemiology at http://www.epidem.com/). Information on the STROBE Initiative is available at http://www.strobe-statement.org.

**References**

1 Ministry of Civil Affairs of the People's Republic of China, Ministry of Public Security of the People's Republic of China, Ministry of Finance of the People's Republic of China. Notice on Further Improving the Relevant Work of de facto unattended children. 2020-12-24. <https://www.gov.cn/zhengce/zhengceku/2021-01/26/content_5582578.htm>.

2 General Office of the State Council of the People's Republic of China. State Council Office on Strengthening Orphan Protection Work Opinions. 2010-11-16. <https://www.gov.cn/gongbao/content/2010/content_1754115.htm>.

3 Ministry of Civil Affairs of the People's Republic of China. The order of Ministry of Civil Affairs of the People's Republic of China. 2010-10-22. <https://www.gov.cn/gongbao/content/2011/content_1808597.htm>.

4 State Council of the People's Republic of China. State Council's Opinion on Strengthening the Care and Protection of Left-behind Children in Rural Areas. 2016-02-14. <https://www.gov.cn/zhengce/content/2016-02/14/content_5041066.htm>.

5 Burghes L. What happens to the children of single parent families? *Bmj.* 1994; **308**(6937): 1114-5.

6 Zhang J, Sun W, Kong Y, Wang C. Reliability and validity of the Center for Epidemiological Studies Depression Scale in 2 special adult samples from rural China. *Compr Psychiatry.* 2012; **53**(8): 1243-51.

7 Dunstan DA, Scott N. Clarification of the cut-off score for Zung's self-rating depression scale. *BMC Psychiatry.* 2019; **19**(1): 177.

8 Pineles BL, Harris AD, Goodman KE. Adverse Maternal and Delivery Outcomes in Children and Very Young (Age ≤13 Years) US Adolescents Compared With Older Adolescents and Adults. *Jama.* 2022; **328**(17): 1766-8.

9 Rubenstein E, Hartley S, Bishop L. Epidemiology of Dementia and Alzheimer Disease in Individuals With Down Syndrome. *JAMA Neurol.* 2020; **77**(2): 262-4.

10 McNemar Q. Note on the sampling error of the difference between correlated proportions or percentages. *Psychometrika.* 1947; **12**: 153-7.

11 Edwards AL. Note on the correction for continuity in testing the significance of the difference between correlated proportions. *Psychometrika.* 1948; **13**(3): 185-7.

12 Ranganathan P, Pramesh CS, Aggarwal R. Common pitfalls in statistical analysis: Absolute risk reduction, relative risk reduction, and number needed to treat. *Perspect Clin Res.* 2016; **7**(1): 51-3.

13 Schechtman E. Odds ratio, relative risk, absolute risk reduction, and the number needed to treat--which of these should we use? *Value Health.* 2002; **5**(5): 431-6.

14 Chow S-C, Wang H, Shao J. Sample Size Calculations in Clinical Research. New York: Chapman and Hall/CRC; 2007.
