# Supplementary material for: Implementation of Primary Psychological Healthcare Policy to Address the Risk of Depression in Underprivileged Children and Adolescents, in the Entire Lower-Middle-Economic-Status City of China: An Observational, Multicenter, and Single-Arm Cohort Study
Source: Depress Anxiety. 2025 Sep 11;2025:5572365. doi: 10.1155/da/5572365 (PMC12446603; doi:10.1155/da/5572365)
Supplement: Supporting Information 3 — The technical book of the Psychological Health Guard for Children and Adolescents Project of China (CPHG). [file 5572365.f3.docx]

Project Work Manual for the Psychological Health Guard for Children and Adolescents Project of China (CPHG)

(Hospital Sector Work Framework)

To thoroughly implement General Secretary Xi Jinping's important instructions on the protection of minors, in early June 2022, in response to prominent issues such as the psychological health, myopia, dental caries, and scoliosis of minors in Sichuan Province, Sichuan Province proposed for the first time to carry out a pilot project for the "Bright Eyes and White Teeth, Righteous Heart and Upright Body" health project. It specifically outlined that Nanchong City would carry out a pilot project focusing on the psychological health of minors under the "Righteous Heart" health project. In accordance with the project requirements, the Nanchong City Minor Protection Center, the Nanchong City Civil Affairs Bureau, and the Nanchong City Mental Health Hospital jointly formulated the "Nanchong City 'Righteous Heart' Health Project Implementation Plan" (Nanwei Office [2022] No. 9). In accordance with the requirements of the plan, combining on-site investigations by the project team, expert discussions, and trial schemes in various work processes, this work manual was developed.

**1. Project Objective**

Establish the "575" service model for promoting the mental health of minors, comprehensively enhancing the mental health level of minors.

5-party linkage: Construct a mental health service model that connects “institutions + schools + families + communities + charitable organizations" in five aspects.

7 major actions: Carry out “seven major actions” including site construction, health education, environment creation, health promotion, health care, capacity building, and service system improvement.

5 project goals: Implement institutionalization through multiple-party linkage, standardize work processes, digitize work throughout, make health decisions precise, and diversify fundraising channels, among other "five aspects."

**2. Project Team**

**2.1 Expert Team**

14 medical guidance experts are responsible for program approval, quality control, and academic research. They come from 8 universities or research institutions including West China School of Public Health at Sichuan University, Peking University, and the Institute of Psychology at the Chinese Academy of Sciences.

There are 19 members in the project guidance expert group (Nanchong City), responsible for program implementation, standard development, impact assessment, and teacher training. These experts come from institutions such as the National Health Commission's Institute of Science and Technology, China West Normal University, North Sichuan Medical College, Southwest Petroleum University, and public mental health institutions.

**2.2 Medical Technology Team**

Responsible for psychological health screening, post-examination medical services, and teacher training.

A total of 17 clinical psychologists and psychiatrists were selected from institutions such as Nanchong Mental Hospital, Nanchong Mental Health Center, and the affiliated hospital of North Sichuan Medical College. They are responsible for psychological health screening, post-examination medical services, and teacher training.

**2.3 On-site work team**

Engaged in community rehabilitation management, home visits, initial interviews, psychological counseling, and offline popular science activities. A total of 10 mental health social organizations are involved. With the Nanchong Mental Health Association as the core, following the principle of one institution per district, the "Evaluation Standards for the Capacity of Social Organizations to Participate in Mental Health Services for Minors" and the "Conditions for Social Organizations to Participate in Services for Minors in Nanchong" are used for pre-audit.

**3. On-site work team**

A total of 10 working groups are established, including: 1 project management group, 5 core working groups for building psychological health assessments, post-medical services, site construction, training, and mental health popularization; 2 coordinating groups for secretarial and logistical support, as well as 2 innovative support groups for publicity and academic support.


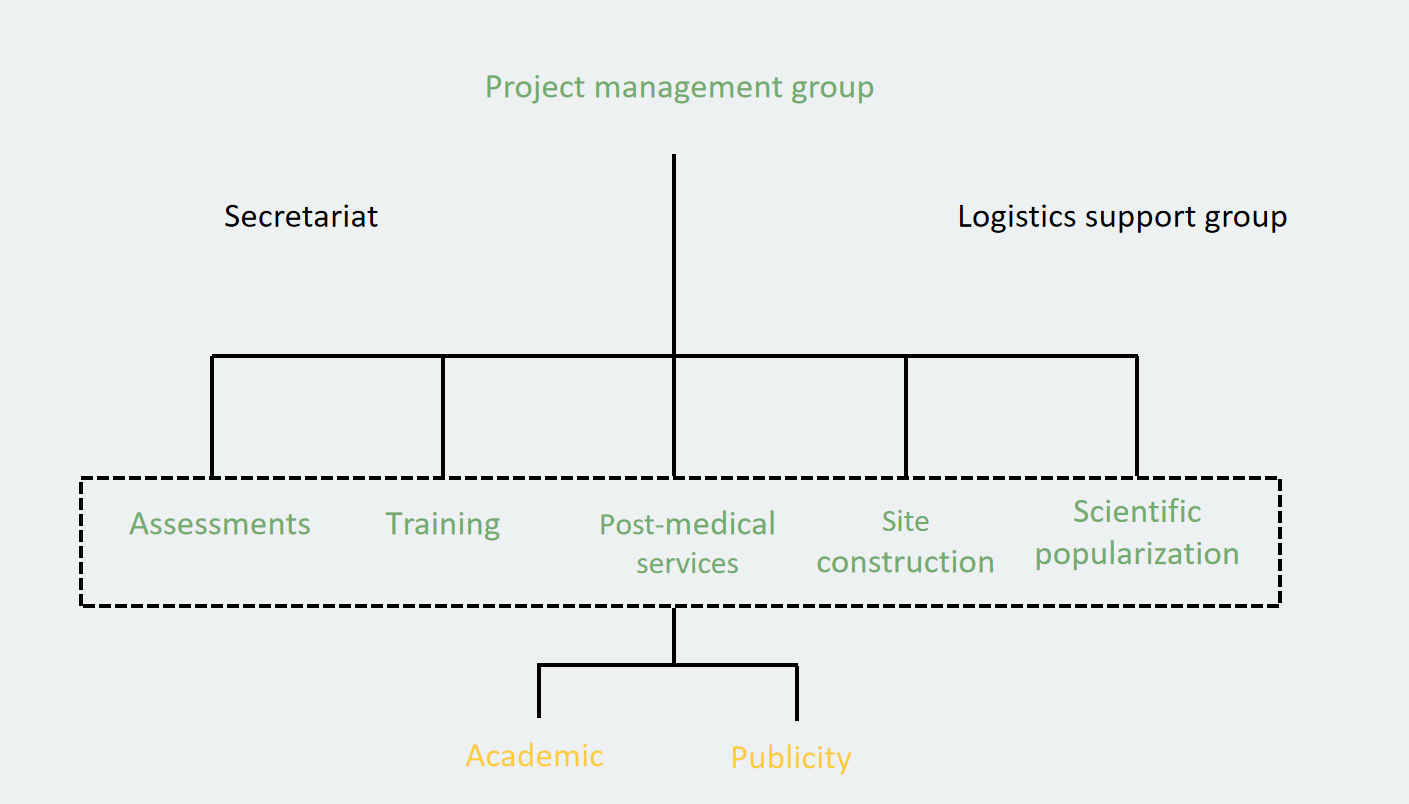


**4. Specific components**

**4.1 “2+2” psychological healthcare services**

The “2+2” psychological healthcare pattern

**4.1.1 Project objectives**

Master the psychological health status of minors and identify individuals with psychological health problems, establish a data platform for children and adolescents' psychological health in Nanchong City, as well as a model for predicting and warning of psychological crises.

**4.1.2 Project Framework**

The first-round screening: Screening students with depressive tendencies;

The Second-round screening: Among people with depressive tendencies, further assess the severity of depression based on evaluation results;

Structured initial interview: For students whose assessment indicates moderate to severe depression, or those identified as at-risk based on teacher observations, structured initial interviews should be conducted by the school's mental health counselor;

Discriminative interview: For students identified by initial interviews as needing further discrimination, parents or guardians are informed by mental health teachers or class teachers, and with their informed consent, a psychiatrist from a public psychiatric hospital is dispatched to the school for a second discriminative interview.


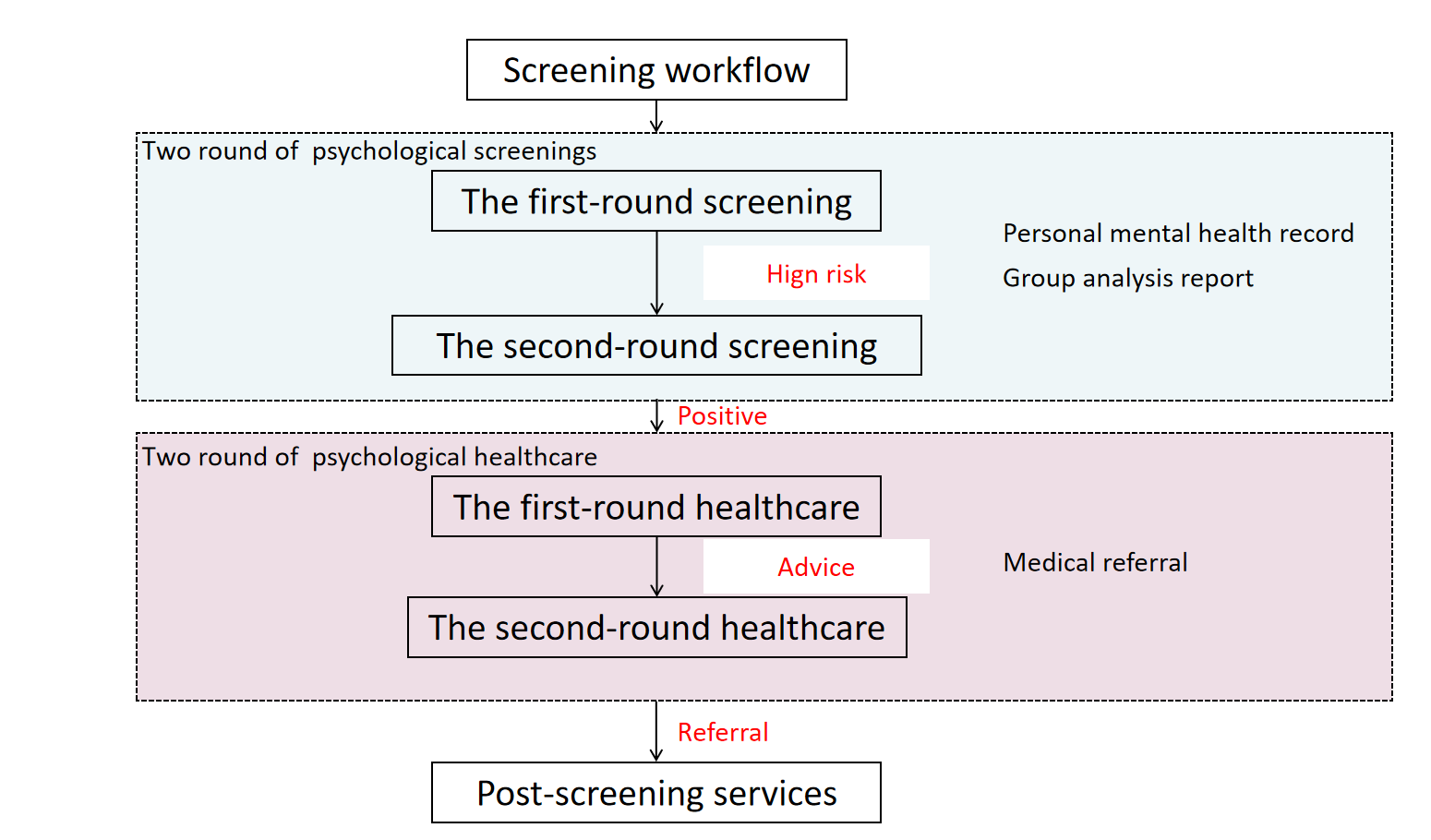


**4.1.3** The evaluation scales

| **Center for Epidemiological Studies-Depression Scale (CES-D)** |
| --- |
| My appetite was poor |
| I could not shark of the blues despite much supports from family and friends |
| I had trouble keeping my mind on what I was doing |
| I felt depressed |
| My sleep was restless |
| I felt sad |
| I could not get going |
| Nothing made me happy |
| I felt like a bad person than other ones |
| I lost interests in my life |
| I talk much less than usual |
| I had ever cried |
| I felt fear |
| I felt bad evaluations to me from other persons |
| I felt alone |
| I was tired to do anything all the time |
| I feel hopeless |
| I fail to go well for my life |
| I had a lot of trouble in many non-sense things |
| I felt no one like me |
| **Self-rating Depression Scale** |
| I feel down-hearted and blue |
| I have crying spells, or feel like it |
| Morning is when I feel the worst |
| I have trouble sleeping at night |
| I eat as not much as I used to |
| I notice that I am losing weight |
| I cannot enjoy when I talk with ones of the opposite sex |
| I have trouble with constipation |
| My heart beats faster than usual |
| I get tired for no reason |
| I find myself restless and can’t keep still |
| I find it hard to do the things I used to |
| My mind is as not clear as it used to be |
| My life is pretty bad |
| I feel hopeful about the future |
| I find it hard to make decisions |
| I am more irritable than usual |
| I lost to enjoy the things I used to |
| I feel that I am not useful and needed |
| I feel that others would be better off if I were dead |

**4.1.4 Pre-test**

Select 2 middle schools and 2 high schools for pre-testing, analyze the reliability and validity, acceptability, and based on the pre-test results, establish the formal evaluation workflow.

**4.1.5 Assessment system**

The "Three-level Comprehensive Mental Health Service Platform for Minors" developed by the National Health Commission's Institute of Science and Technology covers important areas such as psychological health assessment, psychological health record management, psychological crisis prediction and early warning, supervision of high-risk individuals, referral treatment green channel, psychological health education platform, and collaborative data and information sharing. It supports different assessment modes both online and offline.

**4.1.6 Assessment Work Arrangement**

Classes are conducted in two rounds per year, with teachers and students participating simultaneously. Special education schools and welfare institutions organize separate activities. In remote rural schools where left-behind children are more concentrated, offline paper-based questionnaire tests are piloted.

First Semester: Starting from the third week after the beginning of the school year, lasting for 2 weeks;

Second Semester: Starting from the fourth week after the beginning of the school year, lasting for 3 weeks (including the National Day holiday).

**4.1.7 Information Reporting**

At three levels of school, county, and city, write analysis reports by analyzing data. At the same time, at the levels of junior high school and high school, establish predictive and early warning models for mental health issues among minors.

**4.2 Medical services**

End-to-end closed-loop management.

**4.2.1 Work Object**

After the differential interview by clinical physicians, a list of students who need to receive medical services is proposed. The list will be sent back to the mental health teacher, who will inform the parents. After obtaining the parents' informed consent, the children with illnesses will be referred to the hospital for outpatient or inpatient treatment.

**4.2.2 Work objectives**

Promote the recovery of the child through comprehensive services such as psychological intervention, medication and non-pharmacological therapy, and inpatient treatment.

**4.2.3 Workflow**

Based on the patient's condition, doctors make medical recommendations such as close observation, non-drug therapy, drug therapy, or hospitalization, and carry out related medical services.

After treatment, rehabilitated children are transferred to their homes or communities to continue receiving community rehabilitation management. Community mental health specialized services are provided by social psychological service organizations that have been qualified and confirmed by the project team. The organization designates a person to take over the management work, while also completing community registration, conducting home visits, psychological counseling, and other work.


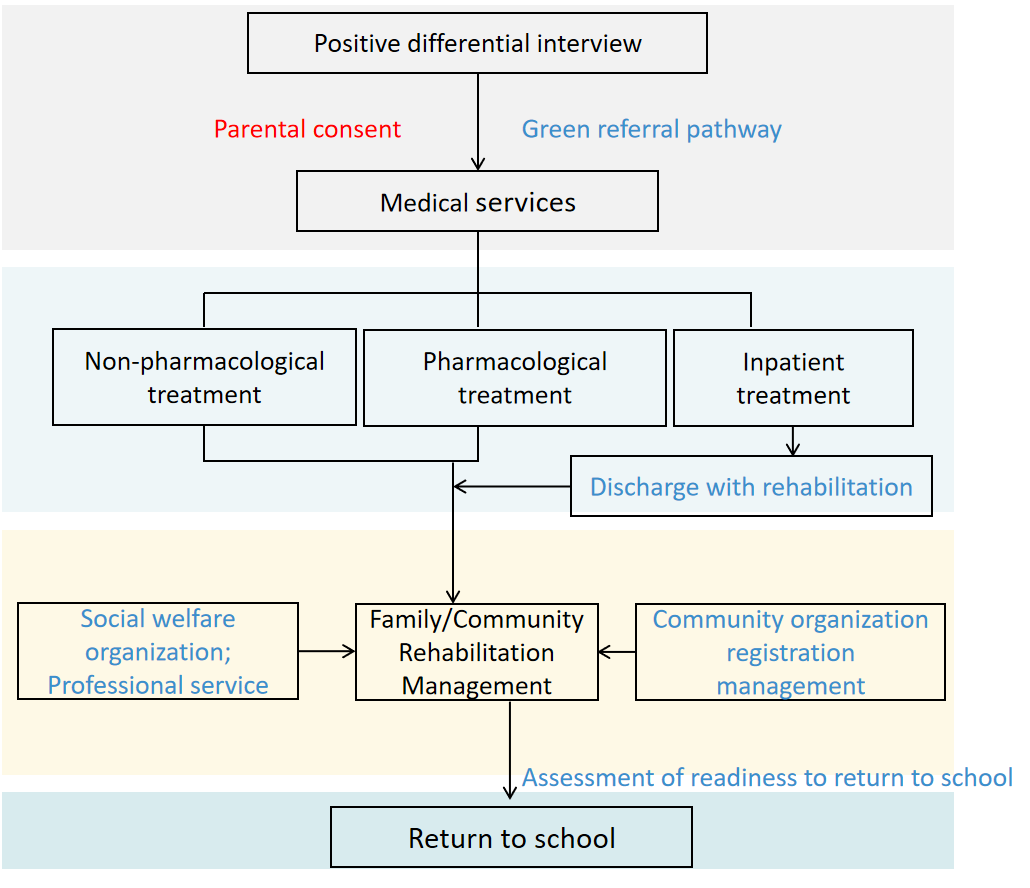


**4.2.4 Assessment of readiness to return to school**

Reentry Capability Assessment: The assessment work is carried out by clinical doctors, and the results are respectively provided to the school and parents.

Involvement of social welfare organizations in the assessment index system for the mental health service capabilities of minors: Through expert discussions, expert inquiries, and statistical analysis, the index system is established for the qualification review and access management of social organizations participating in services for minors.

**4.2.5 Screening of Social Organizations**

From the qualified social organizations, select local service-oriented social organizations based on the ratio of 1 per county/district, and establish the Social Public Welfare Organization Mental Health Service Work Alliance relying on the "Nanchong Mental Health Association", conducting 4 rounds of training and assessment for the core members, unifying and standardizing the service process.

**4.3 Care and support for Underprivileged Children/Adolescents**

Services that combine individual and group approaches

**4.3.1 Definition**

Children who are in survival, development, and safety difficulties due to their own and family reasons, and need the care and assistance of the government and society, including de facto unattended children/adolescents, “left-behind” children/adolescents, “single-parent” children/adolescents, children/adolescents in especially difficult circumstance (CEDC), and orphan.

**4.3.2 Work objectives**

Identify and address the mental health issues faced by vulnerable children at the family, community, and societal levels, enhance the mental health service capabilities of grassroots organizations, social welfare organizations, and community volunteers, and create a conducive living environment for the physical and mental well-being of vulnerable children.

**4.3.3 Work Object**

At the institutional level:

- Nanchong Special Education School, Nanchong First and Second Welfare Institutes

- In each county, select 3 mountain schools where left-behind children are more concentrated

At the individual level:

- In the early stage of psychological health screening, identified disadvantaged children based on basic information, as well as a list provided by homeroom teachers during school surveys. All lists are recorded in a work log and shared with the school's psychological counseling teachers.

**4.4 Mental health knowledge training**

Hierarchical training classification

**4.4.1 Trainees**

Administrative leadership, head teacher, subject teacher, and full-time or part-time mental health teacher.

**4.4.2 Training objectives**

Through categorized training, enhance the risk awareness of administrative leaders, the cognitive level of mental health among primary and secondary school teachers, the teaching abilities of mental health educators, and intervention skills for psychological issues, promoting a deep integration of mental health education with daily teaching activities.

**4.4.3 Overall Work Framework**


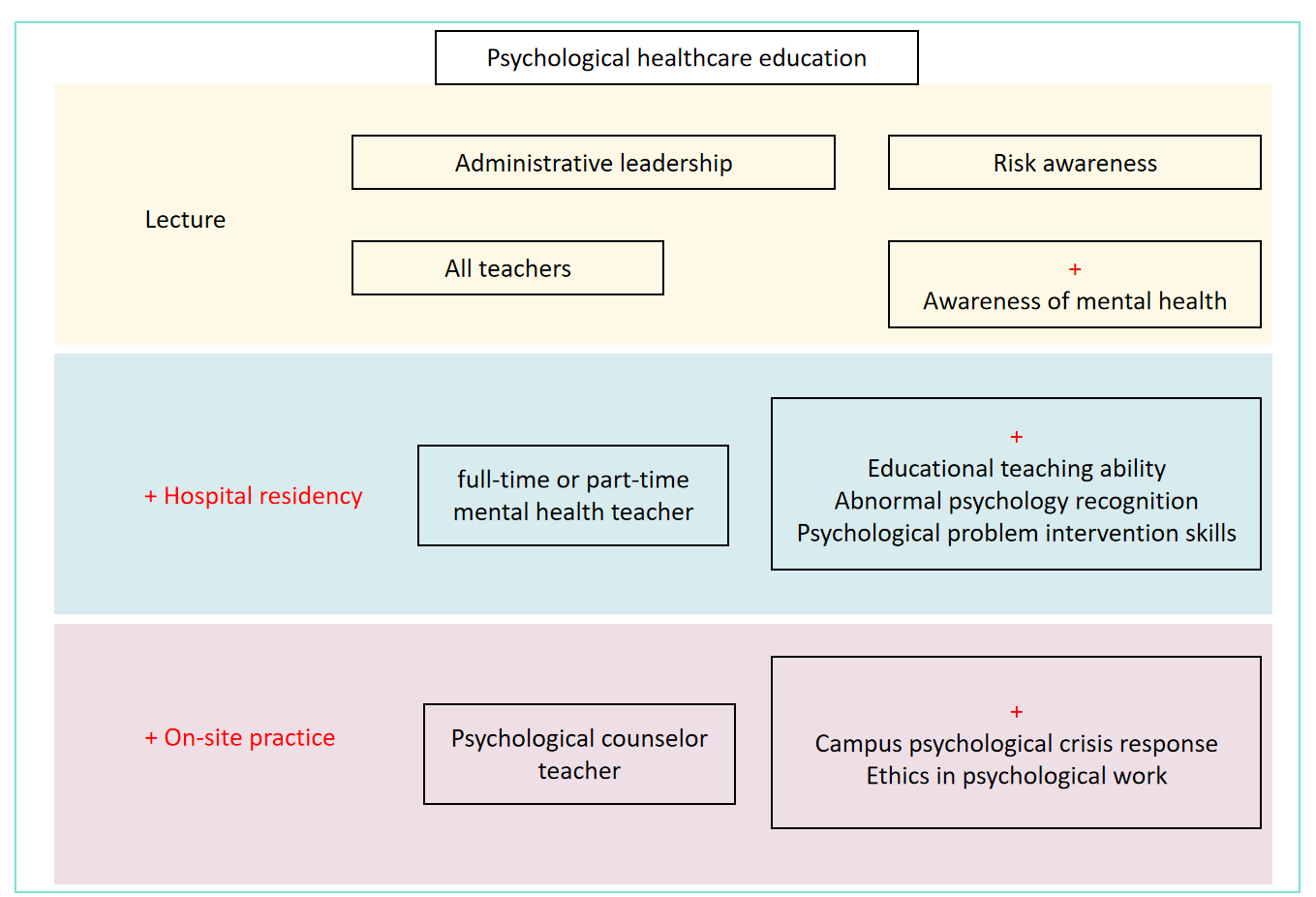


**4.4.4 Training methods**

Expert lectures: organized by county, combining online and offline methods.

Classroom demonstration teaching: organized by schools, using invitation system, with experts providing on-site training.

Hospital training: specialized training courses, open for registration, with 6 sessions per year, each session accommodating 10 teachers.

Intervention skill assistance: for 18 demonstration schools, training provided on-site by clinical psychologists.

**4.4.5 The training content (a total of 40 class hours)**

School Administrative Leadership (2 hours): School Mental Health Education Workflow.

Subject Teachers (12 hours): Developmental Psychology of Children and Adolescents, Positive Psychology, Interpersonal Communication and Emotional Management.

Head Teachers (16 hours): In addition to the subject teachers, include: Identification and Initial Handling of Common Psychological Issues in Students, Psychological Assistance Approaches, Parent-Child Relationships and Family Mental Health Education.

Full-time or part-time Mental Health Teacher (40 hours): In addition to the head teacher foundation, include: Introduction to School Mental Health Education, Psychological Assessment Techniques and Common Psychological Intervention Techniques, Campus Crisis Response System and Process Construction.

**4.4.6 Professional Development for full-time and part-time mental health teacher**

**4.4.6.1 Target population**

Full-time or part-time mental health teacher

**4.4.6.2 Schedule**

Every holiday period lasts for 7 days, with a total of 6 periods held each year.

**4.4.6.3 Educational objectives**

By attending further training in the clinical psychology department of the hospital under full-time teachers, one can master various professional techniques and skills in psychological counseling, correctly use psychological measurement tools, analyze and apply measurement results, provide guidance to students with psychological problems, accurately identify psychological disorders, and make timely referrals.

**4.4.6.4The content of further study (44 class hours)**

Basic skills in psychological counseling, intervention in psychological crises, identification and treatment of severe mental disorders, discriminative interviewing techniques, design and practice of group counseling, issues related to returning to school, ethics and laws, group counseling and individual case supervision.

**4.4.7 Assessment and Evaluation**

The assessment and evaluation of mental health education training adopt a combination of theory and practice, with assessment content set according to different levels of training. Grades are divided into excellent (not exceeding 20%), pass, and fail. Excellent and pass level mental health specialist and part-time teachers can receive a qualification certificate issued by the training center. After homeroom teachers and other teachers pass the training, their qualifications will be certified by the organizing unit.

**4.4.7.1 Assessment for full-time or part-time mental health teacher**

Theoretical Assessment (30% of the total score): The training center will prepare examination papers and answers based on the training content for a knowledge-based assessment. The assessment will cover basic theories and professional knowledge learned during the training.

Skills Assessment (30%): Conducted by the training center and course instructors.

Assessment content: (1) Scenario simulation: such as special topic guidance or lectures (segments); simulating organizing educational research activities; individual student counseling on specific issues; designing and conducting a mental health education class; case studies, etc. (2) On-site analysis: a segment of a class, a counseling case segment, a group counseling segment, a training or educational research activity, a typical teaching example, etc.

Practical Assessment (30%): Comprehensive evaluation by the training center, accounting for 30% of the total score. Assessment content: a professional teacher conducting a mental health education class or submitting a recording of a mental health education class.

Performance in Training (10%): Assessed by the training center, accounting for 10% of the total score. This includes attendance; active participation in training activities; compliance with training discipline and requirements, completion of assignments; outstanding self-study; strong simulation training ability, and active cooperation with course instructors.

**4.4.7.2 Assessment for head teacher**

Theoretical assessment (50%)

The training center will prepare the test papers and answers uniformly according to the training content to conduct knowledge-based assessments, accounting for 50% of the total score. The assessment content includes the basic theories and professional knowledge learned during the training.

Practical assessment (40%)

Comprehensively assessed by the training center. Assessment content: the case presented in the previous class meeting or the integration of mental health knowledge into educational activities.

Performance in training (10%)

Assessment records will be maintained by the organizing unit. This includes attendance; active participation in training activities; adherence to training discipline and requirements, completion of assignments; outstanding self-study; strong simulation training abilities, and active cooperation with teaching staff.

**4.4.7.3 Training on mental health education for all teachers**

The training center prepares exam papers and answers based on the training content for knowledge assessments, while the organizing unit is responsible for maintaining daily assessment records.

**4.5 Position Construction**

Incremental goal-oriented improvement plan

**4.5.1 The goal of building a school counseling center**

In the 9 counties (cities, districts) across the city, following a ratio of 2 schools per county (district), a preliminary plan has been made to establish 18 demonstration stations (points) for the mental health of minors.

**4.5.2 Construction and Evaluation**

**Recommendation for the Construction of School Counseling Rooms in Nanchong City**: Based on the guidelines and norms for the construction of primary and secondary school counseling rooms at the national and provincial levels, combined with the current situation of primary and secondary school counseling room construction in Nanchong City, a preliminary draft of the construction plan is discussed and formulated. After multiple discussions by internal project experts and commissioned external experts, the preliminary construction plan is repeatedly revised and finalized, and then submitted to the Civil Affairs Bureau and the Education and Sports Bureau of the city for review and approval.

**Evaluation Index System for the Service Capacity of School Counseling Rooms in Nanchong City**: Through expert discussions, expert inquiries, and practical testing, the evaluation index system for the service capacity of counseling rooms in primary and secondary schools is preliminarily established for the purpose of evaluating and providing improvement suggestions.

**4.5.3 Promotion of Demonstration Site Construction**

By determining phased goals through city-wide surveys, conducting on-site investigations of proposed construction sites reported from various areas to understand the current situation and needs, and establishing construction plans as follows:

Phase One: Drafting construction plans for counseling rooms.

Phase Two: Construction according to the drafted plans at each site。

Phase Three: Establishing counseling room evaluation standards to guide construction at each site.

Phase Four: Evaluation and acceptance, issuing certifications to sites that meet the evaluation standards.

Phase Five: Establishing stable connections with each site, providing professional support, and establishing a long-term mechanism for sustainable development.

**4.5.4 Trial of demonstration school listing system**

According to the "Service Capability Evaluation Index System of Psychological Counseling Rooms in Primary and Secondary Schools in Nanchong City," schools that have passed self-evaluation, initial evaluation, and re-evaluation will be listed by the "Zhengxin" project team.

Self-evaluation: Demonstration schools compare themselves to the "Evaluation Criteria for the Construction of Psychological Counseling Rooms in Primary and Secondary Schools in Nanchong City," and if they score above 60 points, they can apply for on-site initial evaluation by the field team.

Initial evaluation: After receiving the application, the field team organizes experts to visit the school, evaluate and score through on-site observation, data review, interviews, and other methods.

Re-evaluation: The field team reports the initial evaluation results to the Office of the Municipal Leading Group for the Protection of Minors. After receiving three or more initial evaluation results, the office organizes personnel for batch re-evaluation.

Awarding: Demonstration schools that pass the re-evaluation with a score of 60 points or above are awarded the title of "Guo Cheng You Xin, Nanchong Zhengxin Health Project Demonstration Site."

**4.5.5 Linkage Mechanism Construction**

Establish a service relationship between the school counseling room and the mental health hospital, with the mental health hospital providing technical guidance to the counseling room.

**Mechanism**: Establish online and offline green channels between schools/communities/institutions and specialized mental health hospitals.

**Target**: Individuals or groups identified by the school's psychological counseling room assessment as needing upgraded intervention and treatment.

**Channels**: Mental health platforms, hotlines, internet hospitals.

**Trigger Process**: Appearance of warning signals - report to the school psychologist - inform parents if initial intervention is ineffective - seek help through the green channel - provide support.

**4.6 Mental health knowledge dissemination**

Integration of Online and Offline Popular Science Work Plan

**4.6.1 Work Objectives**

Through continuous, systematic, and professional popularization of mental health knowledge, promote the audience to fully understand the psychological characteristics and major mental health issues of minors at different stages, gradually master the basic skills of prevention and response.

**4.6.2 Target Audience**

parents and youth-related social volunteers, care and support for underprivileged children/adolescents.

**4.6.3 Popular Science Principles**

**Scientific**: The content is correct, does not involve academic or viewpoint controversies, contains no factual, expression, or judgment errors, and is based on reliable scientific evidence.

**Public Welfare**: Does not include any commercial information, does not promote information that contradicts the output and goals of health education.

**Applicability**: Comprehensive coverage of hot topics in mental health issues of public concern, content and dissemination forms are in line with the cultural level and acceptance capacity of the target audience.

**Respect**: Avoid biased information related to policies, ethnicity, gender, religion, culture, age, or race.

**Originality**: Avoid distortion, tampering with others' creations, plagiarism, or theft of others' works, and do not belong to adaptations, translations, annotations, or compilations of others' existing works.

**4.6.4 Online Popular Science Workflow**

**4.6.4.1 Evaluate Audience Needs**

Based on data collected during the previous work processes such as psychological health assessments of minors, teacher training, post-service for patients and children in distress, conduct statistical analysis. Combine specialized surveys, clinical work experience, and expert discussions to determine the cognitive abilities, cognitive levels, cognitive shortcomings, and main points of interest of the target audience, and establish the framework for the work content.

**4.6.4.2 Determine Communication Formats**

Communication Platforms: TikTok, WeChat Official Accounts, WeChat Video Accounts

Priority Order: Videos, comics, animations, popular science articles, and audio

Frequency of Communication: 2 works per week

Timing of Communication: Disseminate knowledge related to specific occasions such as new student enrollment, college entrance exams, major holidays, etc., in a targeted manner.

**4.6.4.3 Creation of Popular Science Works**

Overall Concept: Easy to understand, diverse formats, appealing to the audience

Work Team: Includes creative team, review team, publicity team, and management team

Content Writing: Elaborate on the theme by explaining why a certain phenomenon occurs, why things are done in a certain way, or specific instructions on how to do something.

**4.6.4.4 Work Review and Publication**

Adopt a three-level review system:

Initial review: Review the writing norms, focusing on (1) whether the information sources and evidence are cited, (2) whether the writing logic is clear, and if the content is relevant, (3) whether the article is original and eliminate articles with low innovation.

Professional review: Ensure the professionalism of popular science content to prevent social confusion, public panic, or mental and physical health damage caused by insufficiently scientific or ambiguous information expression.

Normative review: Ensure no conflicts with laws and regulations, social norms, ethical standards, or authoritative information to avoid negative public opinion.

**4.6.4.5 Dissemination Effect Evaluation**

Special surveys: Conduct individual and group interviews with parents, teachers, and volunteers once per quarter, and biannually conduct surveys to understand the main issues regarding the acceptability of content and dissemination methods, as well as the incentive effects on behavior change, serving as a basis for further optimization.

Online evaluation: Analyze the reading frequency, sharing frequency, completion rate, etc., of each work once per quarter to optimize dissemination channels and forms.

**4.6.5 Offline Popular Science Workflow**

**4.6.5.1 Establishing Popular Science Work Team**

Volunteer Team: Comprised of university students and social welfare organizations, responsible for the implementation of offline activities.

Planning Team: Consisting of frontline psychological teachers and psychiatrists, responsible for designing activity plans for different themes.

Expert Team: Comprised of experts in mental health education from universities and influential experts from social welfare organizations, responsible for ensuring the quality of activity plans.

Management Team: Selected from key members of the "Zhengxin" Health Project in Nanchong and the main members of the Nanchong Science Popularization Base, responsible for comprehensive management and service throughout the entire process of implementing offline activities.

**4.6.5.2 Popular Science Activities in Schools**

Utilize large-scale mental health-related festivals such as the "525 Mental Health Festival" or the "125 Mental Health Awareness Month" to conduct life education, interpersonal harmony, neuroscience, and other suitable popular science activities for different stages in primary, middle, and high schools. Use various forms such as carnivals, popular science knowledge Q&A sessions, and lectures to cultivate students' awareness of mental health and enhance their ability to maintain mental health, creating a better campus mental health atmosphere.

**4.6.5.3 Popular Science Activities in Communities**

Utilize major mental health-related holidays such as "World Mental Health Day" or "World Sleep Day" to spread popular science knowledge in areas where people gather in villages and urban communities using bulletin boards, LED screens, printed materials, and other methods to enhance citizens' mental health literacy, creating a social environment conducive to the physical and mental health development of minors.

**4.6.5.4 Activity Evaluation**

Special Surveys: Randomly select one remote rural school, one school in urban-rural fringe areas, and one urban school each year for personal interviews or group interviews with students, parents, teachers, volunteers, and school administrators. Conduct a questionnaire survey after the implementation of each new activity plan to understand the main issues existing in terms of the attractiveness, acceptability, and the effects on cognitive and attitude changes of the activity content, serving as a basis for further optimization.

**5. Project guarantee**

**5.1 Secretary Group Work**

Issue sorting feedback, upload and issue information

**5.1.1 Work Objectives**

Establish a comprehensive information platform, comprehensive coordination platform, comprehensive service platform to facilitate the flow of data and information among various groups, and promote the collaborative work of each working group.

**5.1.2 Work Content**

Build the "Pure Heart" Health Project information database, comprehensively collect, analyze, and provide feedback on project operations, regularly prepare work briefings, and provide feedback to various work groups and related departments.

Strengthen the coordination and linkage between the project management team and various work groups, establish a mechanism for issue feedback and resolution, and facilitate the flow of project operations.

Enhance coordination and connectivity, strengthen the forward-looking and anticipatory nature of work, provide high-quality and efficient services and decision-making basis for the management team, and provide information, data, and coordination services for each working group.

**5.2 Logistics Support Team Work**

**5.2.1 Work Objectives**

To improve the various rules and regulations of the logistics support team, enhance the service and support awareness of logistics support personnel, implement logistics management measures, strengthen project activity support and safety management, continuously improve project hardware and software construction, and ensure the steady and efficient operation of the "Healthy Heart" project.

**5.2.2 Job Responsibilities**

Assist the "Positive Heart" Health Project Team in formulating logistics support plans and safety management plans;

Assist the "Positive Heart" Health Project Team in internal management, providing timely and effective logistics support services for other work groups to carry out on-site activities;

Responsible for the management of office space and equipment for the "Positive Heart" Health Project, as well as activity safety management work;

Responsible for ensuring the various activities and meetings of the "Positive Heart" Health Project;

Complete relevant tasks assigned by the "Positive Heart" Health Project Team.

**6. Project Promotion**

**6.1 Work Objectives**

**6.1.1 Innovate the promotion mode to support the mental health of minors.**

Grasp the laws and characteristics of internet communication in the new era, empower technology to unleash the effectiveness of positive online promotion, and launch a series of key columns, quality reports, popular science comics, etc., around the themes of "positive mindset" and the "seven major actions". The promotional content should be able to integrate into the "youth group" and the "post-wave circle", telling stories in the "youth language" and "adolescent language", to "keep up" and "not be absent", and to generate empathy with the audience, guiding the healthy and robust growth of young people mentally.

**6.1.2 Tell a good story of "Zhengxin" and create a unique business card for Nanchong with heart.**

In-depth exploration of the connotation of the "Zhengxin" health project, profound refinement of classic cases, the improvement of content quality and innovative expression should complement each other, forming theme stories that resonate with the core values of Nanchong's development. Through official and self-media dissemination, embed "Zhengxin" into the urban brand image of Nanchong.

**6.1.3 Make a summary of the experience and form a replicable publicity model.**

Nanchong, as a pilot city for the "Zhengxin" Health Project, should scientifically formulate a plan, adhere to the idea of trying out, improving, and perfecting simultaneously, and do a good job of summarizing beneficial experiences and practices in publicity work to form a promotable publicity model.

**6.2 Forms of Publicity**

**6.2.1 Deeply cultivate traditional media to address profound societal topics.**

Organize special interviews, popularize knowledge on mental health, showcase project effectiveness, and report news in newspapers or on television, continuously follow up and report in depth.

**6.2.2 Leverage emerging media to attract social attention through traffic.**

Utilize the momentum of the new media matrix ("two microblogs and one short video platform"), regularly release event videos and reports on new media platforms, promote on major media websites, foster a positive interaction between traditional radio programs and new media, attract more audiences, expand influence, and spread the care and warmth of the municipal party committee and government for the mental health of minors even further.

6.2.3 Engage with community schools and various parties to proactively speak out. Move the publicity front line forward, collaborate with community schools as a whole, resonate on the same frequency, interact well with students, teachers, and parents, proactively share positive stories about the mental health of minors in Nanchong, and speak up for vulnerable groups.

6.2.4 Organize a series of activities to unite the forces of positive public welfare. Carefully plan public fundraising, charity assistance actions, and actively gather various charitable resources through cultural and sports activities, leverage public welfare forces, and promote the development of mental health among minors.

**“正心”健康工程项目工作手册**

**（医院版块工作框架）**

为深入贯彻习近平总书记关于未成年人保护工作的重要指示批示精神，2022年6月初，针对四川省未成年人心理健康、近视、龋齿、脊柱侧弯等突出问题，四川省首次提出开展“明眸皓齿、正心立身”健康工程试点工作，其中明确了南充市围绕未成年心理健康问题试点开展“正心”健康工程。根据项目要求，南充市未成年人保护中心、南充市民政局、南充市身心医院，共同制定了《南充市“正心”健康工程实施方案》（南未领办【2022】9号）。根据方案要求，结合项目组现场调研、专家研讨和各工作环节方案预试，我们制定了本工作手册。

**1. 项目目标**

建立未成年人心理健康促进工作的“575”服务模式，全面提升未成年人心理健康水平。

575：

**5方联动**：构建“机构+学校+家庭+社区+慈善力量”五方衔接的心理健康服务模式；

**7大行动**：开展阵地建设、健康教育、环境营造、健康促进、健康关爱、能力提升、服务体系完善等“七大行动”；

**5项目标**：多方联动制度化、工作流程标准化、工作全程信息化、卫生决策精准化、筹资渠道多元化等“五项”。

**2. 项目团队**

**2.1专家团队**

医疗指导专家14名，负责研究方案审定、质量监控和学术研究。分别来自四川大学华西公共卫生学院、北京大学、中国科学院心理研究所等8所大学或研究机构；

项目指导专家组（南充市）19名，负责实施方案编制、标准研制、效果测评、教师培训。专家分别来自国家卫健委科学技术研究所、西华师范大学、川北医学院、西南石油大学等机构和公立精神卫生机构。

**2.2医疗技术团队**

负责心理健康筛查、检后医学服务和教师进修培训。

共筛选17名临床心理、精神医师医师，分别来自南充市身心医院、南充市精神卫生中心、川北医学院附属医院等，负责心理健康问题筛查、检后医学服务和教师进修培训等；

**2.3 现场工作团队**

参与社区康复管理、家庭访视、初始访谈、心理咨询和线下科普活动等工作。

共10家心理健康社会组织。以南充市精神卫生协会为核心，按照每区县1家机构的原则，采用《社会组织参与未成年人心理健康服务能力评价标准》和《南充市社会组织参与未成年人服务注入条件》进行事前审核。

**3. 工作框架**

共设定10个工作小组，包括：项目管理组1个，组建心理健康测评、检后医学服务、阵地建设、培训和心理健康科普等5个核心工作组；秘书和后勤保障两个协调组，以及宣传和学术两个创新支持组。


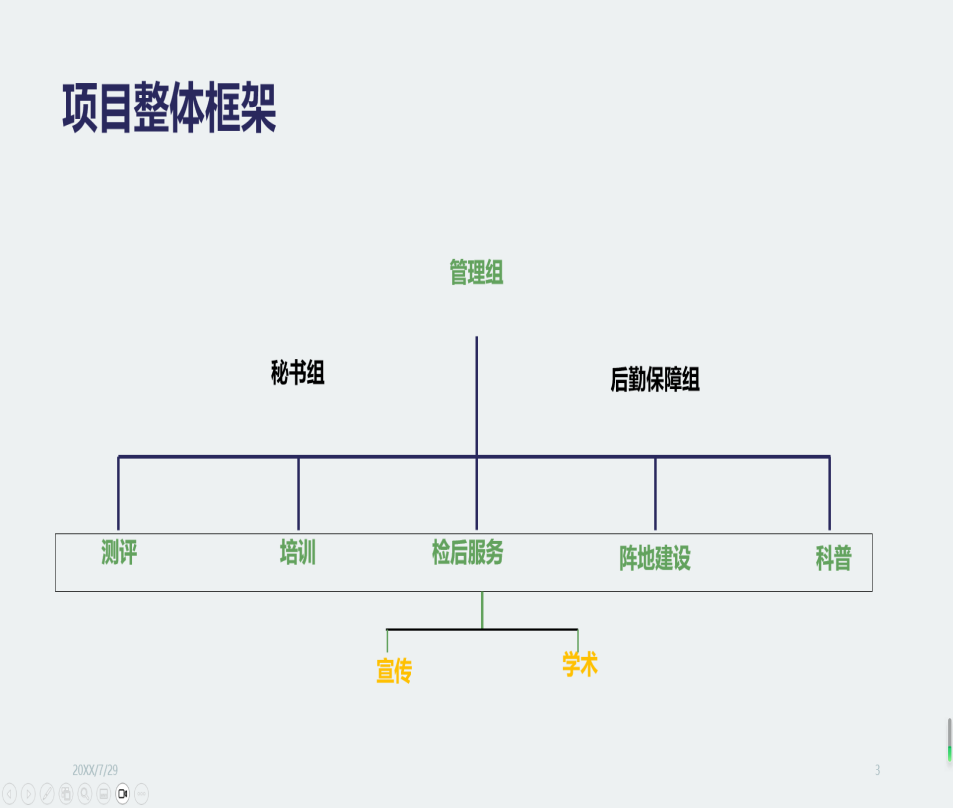


**4. 重点任务**

**4.1 “2+2”心理健康服务**

“2 + 2”筛查模式

**4.1.1工作目标**

掌握未成年人心理健康状况并识别存在心理健康问题的个体，建立南充市儿童青少年心理健康数据平台，以及心理危机预测预警模型。

**4.1.2 工作框架**

初筛：筛查具有抑郁倾向性的学生；

复测：在有抑郁倾向的人群中，进一步通过测评结果判断抑郁等级；

结构化初始访谈：对测评提示中重度抑郁的学生、或班主任观察建议的异常学生，由学校心理健康教师开展结构化初始访谈；

鉴别性访谈：初始访谈后经心理教师建议需要进一步鉴别的学生，由心理教师或班主任知会家长或监护人，在获得知情同意情况下，由公立精神专科医院派遣精神科医生入校开展第二次鉴别性访谈。


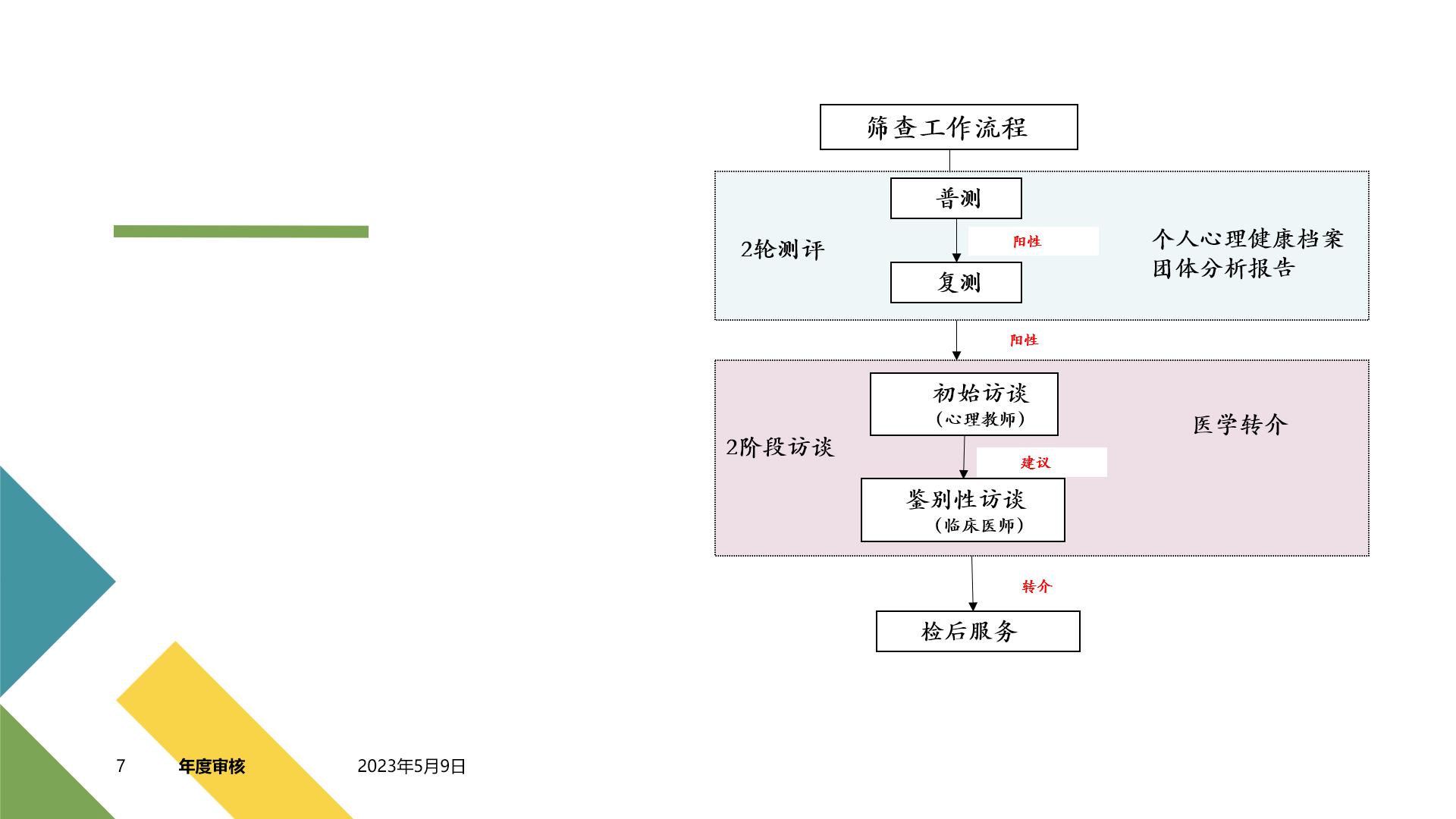


**4.1.3测评量表**

流调中心抑郁量表(CES-D)、抑郁自评量表(SDS)

**4.1.4预测试**

选择初中和高中各2所学校进行预测试，分析信效度、可接受性，根据预测试结果，建立正式测评工作流程。

**4.1.5测评系统**

采用国家卫生健康委科学技术研究所研发的《三级未成年人心理健康综合服务平台》，平台涵盖了心理健康测评、心理健康档案管理、心理危机预测预警、高危个体监管、转介治疗绿色通道、心理健康科普阵地、数据信息共建共享等重要板块。支持线上、线下不同评估模式。

**4.1.6 测评工作安排**

2轮/年，师生同步。特殊教育学校、福利院单独组织。在留守儿童较为集中的边远农村学校，试点实施线下纸质问卷测试。

上学期：开学第三周开始，共2周；

下学期：开学第4周开始，持续3周（含国庆节）。

**4.1.7信息呈报**

分学校、县区和全市三个层面，通过分析数据，撰写分析报告。

同时，分初中、高中两个个层次，建立未成年人心理健康问题的预测、预警模型。

**4.2医学服务**

全流程闭环管理

**4.2.1 工作对象**

经临床医师鉴别性访谈之后，提出建议需要接受医学服务的学生名单，将名单反馈至心理健康教师，由教师知会家长，经家长知情同意后，将病患儿童转至医院接受门诊或住院治疗。

**4.2.2工作目标**

通过心理干预、药物和非药物治疗、住院治疗等综合服务手段，促进患儿康复。

**4.2.3 工作流程**

根据患者情况，医生做出密切观察、非药物治疗、药物治疗或住院治疗等医学建议，并开展相关医学服务工作。

经治疗后，康复儿童转至家庭或社区继续接受社区康复管理。社区心理健康专业化服务工作由通过资格审查且经项目组确认的社会心理服务组织负责。该组织指定专人接手管理工作，同时完成社区备案，开展家庭访视、心理咨询等工作。


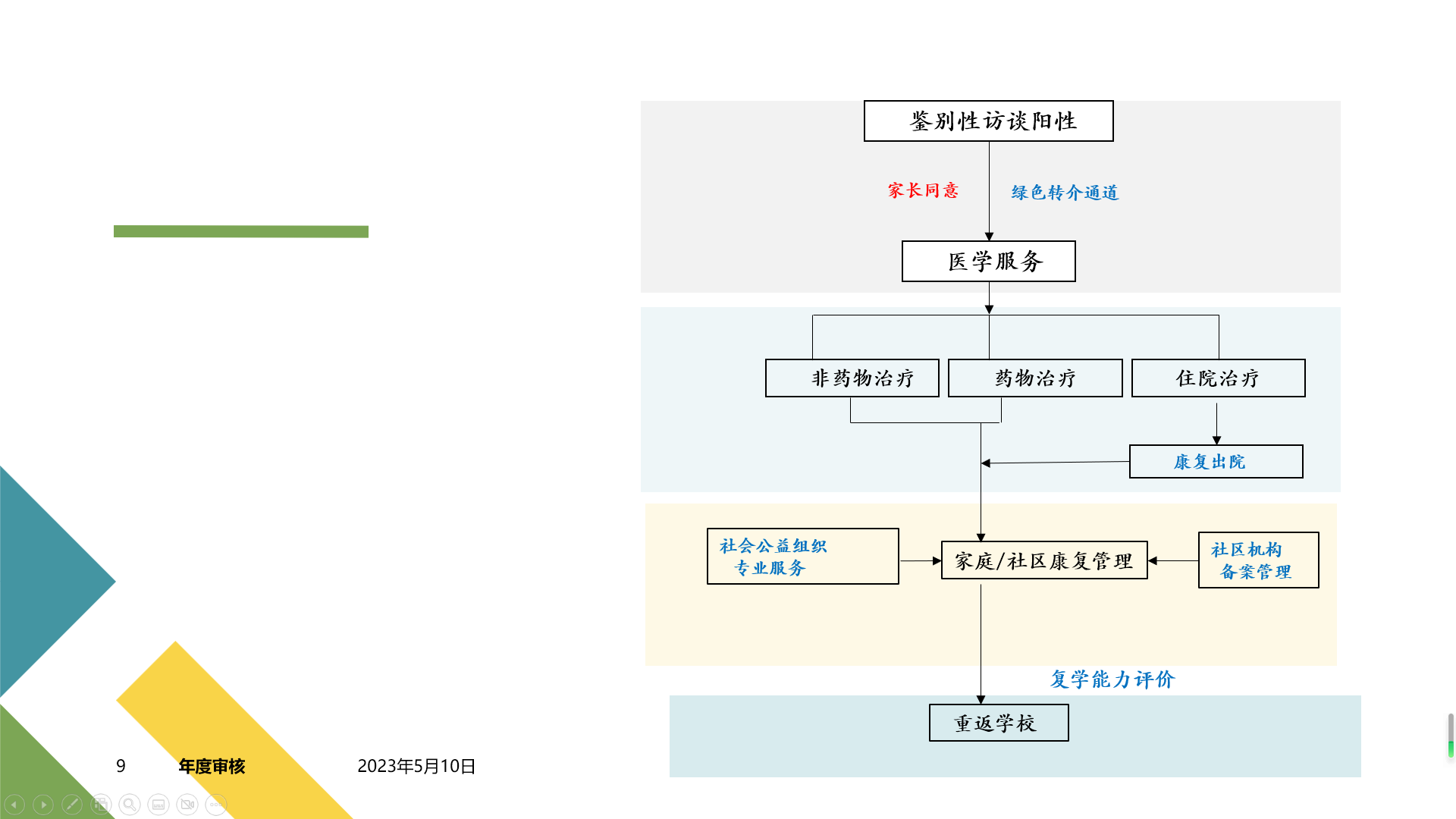


**4.2.4能力评估**

**复学能力评价：** 评估工作由临床医生开展，结果分别反馈至学校和家长。

**社会公益组织参与未成年人心理健康服务能力评价指标体系：**经过专家研讨、专家函询和统计分析，建立该指标体系，用于对社会组织参与未成年人服务的资格审查和准入管理。

**4.2.5 社会组织筛选**

从通过资格审查的社会组织中，初步按照1个/县区选择地方化服务的社会组织，并依托《南充市心理卫生协会》建立社会公益组织心理健康服务工作联盟，对骨干成员开展4轮培训和考核，统一和规范服务流程。

**4.3困境儿童关爱**

个体和群体相结合的服务

**4.3.1困境儿童定义**

因自身和家庭原因而陷入生存、发展和安全困境，需要政府和社会予以关心帮助的儿童。包括家庭贫困儿童、自身残疾或身体出现严重疾病的儿童、家庭监护缺失或监护不当的儿童（含留守儿童）、遭受虐待、意外伤害、不法侵害等导致人身安全受到威胁或侵害的儿童、孤儿及流浪儿童等5个类别。

**4.3.2 工作目标**

识别并处理困境儿童存在的心理健康问题，从家庭、社区和社会层面，提升基层组织、社会公益组织和社区志愿者心理健康服务能力，营造有利于困境儿童身心健康的生活环境。

**4.3.3工作对象**

机构层面：

南充市特殊教育学校、南充市第一、二福利院

每个县区，选择3所留守儿童较为集中的山区学校

个体层面：

前期心理健康普测中，根据基础信息所识别的困境儿童，以及在学校调研，由班主任老师所提供的名单。所有名单均建立工作台账，与学校心理咨询室教师共享。

**4.4培训工作体系**

分类分层次培训

**4.4.1 培训对象**

学校行政领导、班主任、心理健康专兼职教师、学科教师

**4.4.2 培训目标**

通过分类化培训，提升行政领导风险意识、中小学教师心理健康认知水平、心理健康教师教育教学能力和心理问题干预技能，促进心理健康教育与日常教学活动的深度融合。

**4.4.3整体工作框架**


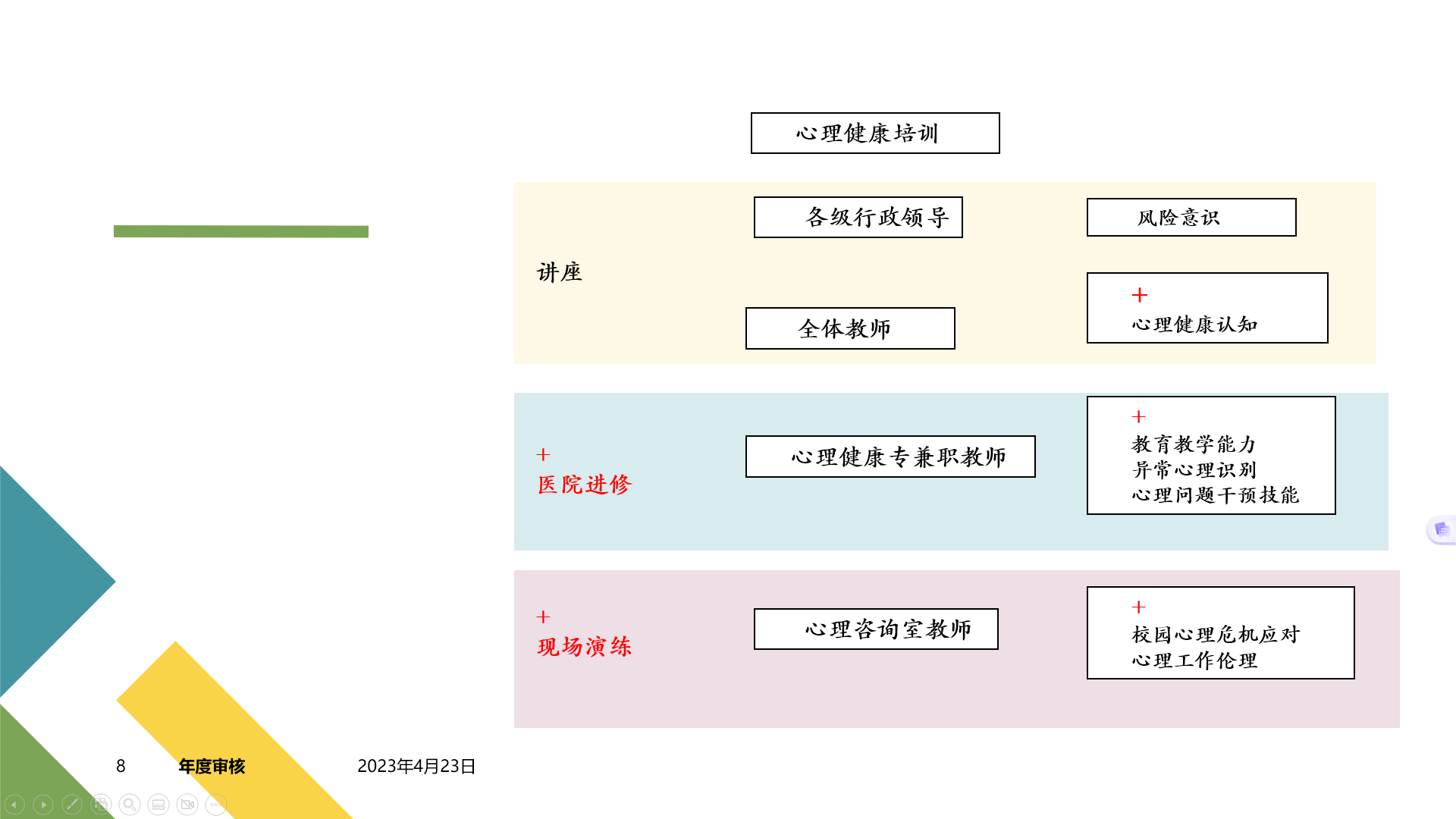


**4.4.4培训方式**

专家讲座：以县区为单位组织，线上线下相结合；

课堂示范教学：以学校为单位组织，采用邀请方式，由专家入校培训；

医院进修：专项培训班，采用报名制，每年6期，每期10名教师。

干预技能辅助：针对18所示范学校，由临床心理医师入校培训。

**4.4.5 培训内容（总共40课时）**

学校行政领导（2学时）：学校心理健康教育工作流程

学科教师（12学时）：儿童青少年发展心理学、积极心理学、人际沟通与情绪管理。

班主任（16学时）：在学科教师基础上，增加：

学生常见心理问题识别与初步处理、心理援助途径、亲子关系与家庭心理健康教育

心理健康专兼职教师（40学时）：在班主任教师基础上，增加：

学校心理健康教育概论、心理测评技术与心理干预常用技术、校园危机应对体系及流程构建。

**4.4.6心理健康教育专职教师进修**

**4.4.**6.1进修对象

各中小学校专职（或兼职）从事心理健康教育教学工作的教师。

**4.4.6.2 时间安排**

每年假期，每期7天，每年共开展6期。

**4.4.6.3进修目标**

通过专职教师到医院临床心理科进修，熟练掌握多种心理咨询的专业技术和技巧，并能够正确使用心理测量工具，分析和运用测量结果，对有心理问题的学生进行辅导，准确识别心理疾病并及时转介。

**4.4.6.4进修内容（44学时）**

心理咨询基本技巧、心理危机干预、严重精神障碍的识别与处理、鉴别性访谈技术、团体辅导的设计与实践、复学相关问题，伦理与法律、团体辅导与个案督导

**4.4.7考核与评价**

心理健康教育培训的考核与评价采用理论与实践相结合的方式，根据不同层次的培训设定考核内容。成绩分为优秀（不超过20%）、及格、不及格三个等级。优秀和及格的心理健康专兼职教师可获得培训中心颁发的资格证书，班主任和其他教师培训合格后，由主办单位进行资格认定。

**4.4.7.1心理健康教育专兼职教师考核**

（1）理论考核（总成绩的30%）：由培训中心根据培训内容统一编制试卷和答案，进行知识类考核，考核内容包括培训中所学到的基本理论和专业知识。

（2）技能考核（30%）：由培训中心和讲课教师负责考核。

考核内容：（1）情境模拟：如专题辅导或讲座（片断）；模拟组织教研活动；个别问题学生辅导；模拟设计并上一堂心理健康教育课；案例分析等。（2）现场评析：一节课片断、一个咨询案例片段、一个团体辅导片断、一次培训活动或教研活动、一个典型教师的教例等。

（3）实践考核（30%）

由培训中心综合评定，占总成绩的30%。考核内容：专业教师上一节心理健康教育课或提交一盘心理健康教育录像课。

（4）培训中的表现（10%）

由培训中心负责进行考核记录，占总成绩的10%。包括出勤情况；主动参与培训活动；遵守培训纪律和各项要求，完成各科作业情况；自学突出；模拟训练能力强，积极配合讲课教师。

**4.4.7.2 班主任心理健康教育培训**

（1）理论考核（50%）

由培训中心根据培训内容统一编制试卷和答案，进行知识类考核，本分数占总成绩的50%。考核内容包括在培训中所学到的基本理论和专业知识。

（2）实践考核（40%）

由培训中心综合评定。考核内容：上一节班会课，或在教育教学中融入心理健康知识的案例。

（3）培训中的表现（10%）

由主办单位负责进行考核记录。包括出勤情况；主动参与培训活动；遵守培训纪律和各项要求，完成各科作业情况；自学突出；模拟训练能力强，积极配合讲课教师。

**4.4.7.3全体教师心理健康教育培训**

由培训中心根据培训内容统一编制试卷和答案，进行知识类考核，主办单位负责进行日常考核记录。

**4.5阵地建设**

阶段性目标导向式的提升计划

**4.5.1学校心理辅导室建设目标**

在全市9个县（市、区），按照2校/县（区），初步建设18个未成年人心理健康工作示范站(点)。

**4.5.2 建设与评价**

**南充市学校心理咨询室建设推荐方案：**根据国、省中小学心理辅导室建设指南和规范，结合南充市中小学心理辅导室建设现状，研讨拟定阵地建设方案初稿，经项目内部专家、委托外部专家多方论证，反复修改初步完成建设方案，提交市民政局、市教体局审核发文。

**南充市学校心理辅导室服务能力评价指标体系：**通过专家研讨、专家函询和实践测试，初步建立该指标体系，用于对中小学校咨询室服务能力的评价和改进建议。

**4.5.3 推进示范站点建设**

通过全市调研确定阶段性目标，对各地上报的拟建站点进行现场调查，掌握现状及需求，确立建设规划：

第一阶段：拟定心理辅导室建设方案；

第二阶段：各站点对照方案建设；

第三阶段：制定心理辅导室评估标准，指导各站点建设；

第四阶段：评估验收，对达到评估标准的站点授牌；

第五阶段：与各站点建立稳定链接，提供专业支持，为可持续发展建立长效机制。

**4.5.4 试行示范学校挂牌制度**

依据《南充市中小学校心理辅导室服务能力评价指标体系》，对通过自评、初评和复评的学校，由“正心”项目组挂牌。

自评：示范学校对照《南充市中小学校心理辅导室建设评估标准》自评，得分在60分以上再申请阵地组现场初评。

初评：阵地组接到申请后，组织专家到场地，通过现场观察、资料查阅、访谈等方式进行评价打分。

复评：阵地组将初评结果向市未成年人保护工作领导小组办公室汇报，办公室在收到3个及以上初评结果后，组织人员进行批量复评。

授牌：对通过复评，分数在60分及以上的示范学校授予“果诚佑心 南充市正心健康工程示范点”。

**4.5.5 联动机制建设**

由学校咨询室和身心医院建立服务关系，由身心医院对咨询室开展技术指导。

（1）机制：建立学校/社区/机构与精神心理专科医院之间的线上线下绿色通道。

（2）对象：经各学校心理辅导室评估，需要升级干预和处理的个体或群体。

（3）途径：心理健康平台，热线、互联网医院。

（4）触发流程：预警信号出现-报告心理老师-初步干预无效后-通知家长知情-绿色通道求援-支援。

**4.6科普工作**

线上线下相结合的科普工作规划

**4.6.1 工作目标**

通过持续、系统性和专业化的心理健康知识普及，促进受众充分认识未成年人在不同阶段的心理健康特点和主要心理健康问题，逐步掌握预防和应对的基本技能。

**4.6.2 科普对象**

学生家长、教师、未成年人工作相关人员或志愿者

**4.6.3 科普原则**

（1）科学：内容正确，不涉及学术或观点争议，没有事实、表述和评判上的错误，有可靠的科学证据。

（2）公益：不包含任何商业信息，不宣传与健康教育产出和目标相抵触的信息。

（3）适用：全面覆盖公众关注的热点心理健康问题，内容与传播形式与目标人群文化水平与接受能力相符合。

（4）尊重：避免出现在政策、民族、性别、宗教、文化、年龄或种族等方面产生偏见的信息。

（5）原创：避免出现歪曲、篡改他人创作或抄袭、剽窃他人创作而产生的作品，亦不属于改编、翻译、注释、整理他人已有创作而产生的作品。

**4.6.4线上科普工作流程**

**4.6.4.1评估受众需求**

根据前期未成年人心理健康测评、教师培训、病患和困境儿童检后服务等工作过程中采集的数据，开展统计分析，结合专项调研、临床工作经验和专家研讨，分别确定目标人群认知能力、认知水平、认知短板和主要兴趣点，确定工作内容框架。

**4.6.4.2 确定传播形式**

传播载体：抖音、微信公众号、微信视频号

优先顺序：视频、漫画、动画、科普文章和音频

传播频度：2个作品/周

传播时机：根据新生入校、中高考、主要节日等时机，针对性地传播与这些时机相关的知识。

**4.6.4.3科普作品创作**

总体思路：通俗易懂、形式多样、人群喜闻乐见

工作团队：包括创作团队、审核团队、宣传团队和管理团队

内容编写：围绕主题阐述为什么会呈现这种现象、为什么这样做，或具体怎么做等

**4.6.4.4 作品审核与发布**

采用三级审核制度：

初审：对行文规范进行审核，审查重点包括，（1）是否注明信息出处和证据来源，（2）行文逻辑是否清晰，文章内容是否切题。（3）文章是否原创，筛除创新性低的文章。

专业审核：确保科普内容的专业性，不会因信息表达不够科学准确或有歧义，引起社会混乱和公众恐慌或对公众造成身心健康伤害。

规范性审核：确保与法律法规、社会规范、伦理道德、权威信息冲突，避免引发负面社会舆论。

**4.6.4.5 传播效果评价**

**专项调研：**每季度1次，对家长、教师和志愿者的个人访谈和小组访谈，每半年1次，开展问卷调查，从作品内容和传播形式的可接受性、行为改变的激励效果等方面，了解存在的主要问题，作为进一步优化的依据。

**网络评价**：每个季度一次，对每个作品的阅读次数、分享次数、阅读完成率等进行分析，以优化传播途径与形式。

**4.6.5 线下科普工作流程**

**4.6.5.1 创建科普工作团队**

**志愿团队：**由高校学生和社会公益组织组成，负责线下活动的实施。

**策划团队：**由一线心理教师、精神科医生组成，完成对不同主题下的活动方案的设计。

**专家团队：**由高校心理健康教育专家、社会公益组织中有影响力的专家组成，对活动方案的质量进行把控。

**管理团队：**从南充“正心”健康工程、南充市科普基地主要成员筛选组成，主要职责是为线下活动实施的全过程进行全方位的管理和服务

**4.6.5.2 科普活动进校园**

利用“525心理健康节”或“125心理健康活动月”等大型的心理健康相关节日，在大中小学开展生命教育、人际和谐、脑科学等适宜不同阶段的科普活动，采用游园会、科普知识答疑、科普讲座等多种形式培养学生的心理健康意识、提升学生心理健康维护能力，营造更好的校园心理健康氛围。

**4.6.5.3科普活动进社区**

利用“世界精神卫生日”或者“世界睡眠日”等大型的精神卫生相关节日，在村社和城镇社区的人群聚居处，利用公告栏、LED屏幕、纸质资料等多种方式传播科普知识，提升公民的心理健康素养，营造适宜于未成年人身心健康发展的社会环境。

**4.6.5.4 活动效果评价**

专项调研：每年随机选取校园活动中偏远农村学校、城乡结合部学校、城市学校各1个，对学生、家长、教师、志愿者、学校管理者进行个人访谈或小组访谈，对每个新的活动方案实施后进行问卷调查，从活动内容的趣味性、可接受性，以及对认知和态度改变的效果等方面，了解存在的主要问题，作为进一步优化的依据。

**5.项目保障**

**5.1秘书组工作**

问题梳理反馈，上传下达信息

**5.1.1 工作目标**

建立综合信息平台、综合协调平台、综合服务平台，畅通数据和信息在各小组的流动，推动各工作小组的工作协同。

**5.1.2工作内容**

（1）构建“正心”健康工程信息资料库，全方位收集、分析、反馈项目运行情况，定期编制工作简报，反馈各工作组及相关部门。

（2）强化项目管理组与各工作组间的协调联动，建立问题反馈及解决机制，打通项目运行“经脉”及“枢纽”。

（3）加强协调衔接，增强工作的超前性和预见性，为管理组提供优质高效的服务和决策依据，为各工作组提供信息、资料及协调服务。

**5.2后勤保障组工作**

**5.2.1工作目标**

健全后勤保障组各项规章制度，增强后勤保障人员的服务和保障意识，落实后勤管理措施，强化项目活动保障及安全管理，不断提升项目工作硬件、软件建设，保障“正心”健康工程项目稳步、高效运行。

**5.2.2 工作内容**

1. 协助“正心”健康工程项目组制定后勤保障方案和安全管理方案；
2. 协助“正心”健康工程项目组进行内务管理，为其他工作组开展现场活动提供及时有效的后勤保障服务；
3. 负责“正心”健康工程项目办公场地、设备的管理和活动安全管理工作；
4. 负责“正心”健康工程项目各类活动、会议的保障工作；

5、完成“正心”健康工程项目组交办的相关工作。

**6.项目宣传**

**6.1 工作目标**

**6.1.1**创新宣传模式，助力未成年人心理健康。把握新时代互联网传播规律和特点，以技术赋能释放网上正面宣传效能，围绕“正心”“七大行动”推出一批重点栏目、精品报道、科普漫画等，宣传内容要能融进“青年群”“后浪圈”讲好“青年话”“少年语”，“不落伍”“不缺席”，要能与受众产生共情，引导青少年心理健康茁壮成长。

**6.1.**2讲好正心故事，用心打造南充特色名片。深度挖掘“正心”健康工程内涵，深刻提炼经典案例，内容提质与表达创新要相得益彰，形成契合南充发展核心价值的主题故事，通过官媒、自媒体传播，将“正心”嵌入南充城市品牌形象。

**6.**1.3做好经验总结，形成可复制宣传模式。南充作为“正心”健康工程试点城市，要科学制定方案，坚持边试点、边改进、边完善的思路，在宣传工作中做好有益经验、做法的提炼总结，形成可推广宣传模式。

**6.2 宣传形式**

6.2.1深耕传统媒体，形成深度社会话题。在报纸或电视台组织专题采访、心理健康知识科普、项目成效展示、新闻报道，持续追踪，深入报道。

6.2.2借力新兴媒体，用流量引社会关注。借势新媒体矩阵（“两微一抖”），定期在新媒体上发布活动视频和报道，在大型媒体网站上推播，形成传统电台节目和新媒体的良性互动，吸引更多受众，扩大影响力，把市委、市政府对未成年心理健康的关心和温暖传得更远。

6.2.3走进社区学校，多方联动主动发声。宣传阵地前移，与社区学校整体联动、同频共振，与学生、老师、家长良好互动，主动讲好南充未成年人心理健康故事，为弱势群体发声。

6.2.4组织系列活动，凝聚正心公益力量。精心策划公益募捐、爱心资助行动、文体活动积极汇集各类慈善资源，发挥公益力量，推动未成年人心理健康事业发展。
